# A KAM Approach to the Inviscid Limit for the 2D Navier–Stokes Equations

**DOI:** 10.1007/s00023-023-01408-9

**Published:** 2024-02-05

**Authors:** Luca Franzoi, Riccardo Montalto

**Affiliations:** Dipartimento di Matematica “Federigo Enriques”, Università degli Studi di Milano, Via Cesare Saldini 50, 20133 Milan, Italy

**Keywords:** 35Q30, 35Q31, 37K55, 76M45

## Abstract

In this paper, we investigate the inviscid limit $\nu \rightarrow 0$ for time-quasi-periodic solutions of the incompressible Navier–Stokes equations on the two-dimensional torus ${\mathbb {T}}^2$, with a small time-quasi-periodic external force. More precisely, we construct solutions of the forced Navier–Stokes equation, bifurcating from a given time quasi-periodic solution of the incompressible Euler equations and admitting vanishing viscosity limit to the latter, uniformly for all times and independently of the size of the external perturbation. Our proof is based on the construction of an approximate solution, up to an error of order $O(\nu ^2)$ and on a fixed point argument starting with this new approximate solution. A fundamental step is to prove the invertibility of the linearized Navier–Stokes operator at a quasi-periodic solution of the Euler equation, with smallness conditions and estimates which are uniform with respect to the viscosity parameter. To the best of our knowledge, this is the first positive result for the inviscid limit problem that is global and uniform in time and it is the first KAM result in the framework of the singular limit problems.

## Introduction

In this paper, we consider the two-dimensional Navier–Stokes equations for an incompressible fluid on the two-dimensional torus ${\mathbb {T}}^2$, ${\mathbb {T}}:= {\mathbb {R}}/ 2 \pi {\mathbb {Z}}$, with a small time quasi-periodic forcing term

1.1 $$\begin{aligned} {\left\{ \begin{array}{ll} \partial _t U + U \cdot \nabla U - \nu \Delta U + \nabla p = \varepsilon f(\omega t, x) \\ {\,\text{ div }\,}U = 0 \end{array}\right. } \end{aligned}$$

where $\varepsilon \in (0, 1)$ is a small parameter, $\omega \in {\mathbb {R}}^d$ is a Diophantine *d*-dimensional vector, $\nu > 0$ is the viscosity parameter, the external force *f* belongs to ${{\mathcal {C}}}^q({\mathbb {T}}^d \times {\mathbb {T}}^2, {\mathbb {R}}^2)$ for some integer $q > 0$ large enough, $U = (U_1, U_2): {\mathbb {R}}\times {\mathbb {T}}^2 \rightarrow {\mathbb {R}}^2$ is the velocity field, and $p: {\mathbb {R}}\times {\mathbb {T}}^2 \rightarrow {\mathbb {R}}$ is the pressure. The main purpose of this paper is to investigate the inviscid limit of the Navier–Stokes equation from the perspective of the KAM (Kolmogorov–Arnold–Moser) theory for PDEs, which in a broad sense is the theory of the existence and the stability of periodic, quasi-periodic and almost periodic solutions for Partial Differential Equations. For the forced Euler equations, namely (1.1) with $\nu =0$, quasi-periodic solutions have been constructed in Baldi and Montalto [4]. We give now the informal statement of our result.

### Informal Theorem

Let $(U_{e}(t,x),p_e(t,x))= (\breve{U}_{e}(\varphi ,x),\breve{p}_{e}(\varphi ,x))|_{\varphi =\omega t}$, $\varphi \in {\mathbb {T}}^d$, be a quasi-periodic solution of (1.1) with $\nu =0$ constructed in [4]. Then, there exists a quasi-periodic solution $(U_{\nu }(t,x),p_{\nu }(t,x))= (\breve{U}_{\nu }(\varphi ,x),\breve{p}_{\nu }(\varphi ,x))|_{\varphi =\omega t}$ of (1.1) bifurcating from the Euler solution $(U_{e}(t,x),p_e(t,x))$ with respect to the viscosity parameter $\nu \rightarrow 0$, with rate of convergence $O(\nu )$ uniform in $\varepsilon \ll 1$ and uniform for all times $t\in {\mathbb {R}}$.

As a consequence of our result, we obtain families of initial data for which the corresponding global quasi-periodic solutions of the Navier–Stokes equations converge to the ones of the Euler equation with a rate of convergence $O(\nu )$, uniformly in time. The main difficulty is that this is a *singular perturbation problem*, namely there is a small parameter in front of the highest order derivative. To the best of our knowledge, this is both the first result in which one exhibits non-trivial, time-dependent solutions of the Navier–Stokes equations converging globally and uniformly in time to the ones of the Euler equation in the vanishing viscosity limit $\nu \rightarrow 0$ and the first KAM result in the context of singular limit problems for PDEs.

The zero-viscosity limit of the incompressible Navier–Stokes equations in bounded domains is one of the most challenging problems in Fluid Mechanics. The first results for smooth initial data ($H^s$ with $s \gg 0$ large enough) have been proved by Kato [33, 34], Swann [43], Constantin [15] and Masmoudi [38] in the Euclidean domain ${\mathbb {R}}^n$ or in the periodic box ${\mathbb {T}}^n$, $n=2,3$. For instance, it is proved in [38] that, if the initial velocity field $u_0 \in H^s({\mathbb {T}}^n)$, $s > n/2 + 1$, then the corresponding solutions $u_\nu (t, x)$ of Navier–Stokes and *u*(*t*, *x*) of Euler, defined on $[0, T] \times {\mathbb {T}}^n$, satisfy

$$\begin{aligned} \begin{aligned}&\Vert u_\nu - u \Vert _{L^\infty ([0, T], H^s)} \rightarrow 0 \quad \text {as} \quad \nu \rightarrow 0 \\&\text {and for} \quad s' < s \quad \quad \Vert u_\nu (t) - u(t) \Vert _{H^{s'}} \lesssim (\nu t)^{\frac{s - s'}{2}}, \quad \forall t \in [0, T]. \end{aligned} \end{aligned}$$

It is immediate to notice that the latter estimate holds only on finite time intervals and it is not uniform in time, with the estimate of the difference $u_\nu (t) - u(t)$ eventually diverging as $t \rightarrow + \infty$. For $n=2$, this kind of result has been proved in low regularity by Chemin [13] and Seis [42], with rates of convergence in $L^2$. We also mention similar results for non-smooth vorticity. In particular, the inviscid limit of Navier Stokes equation has been addressed in the case of vortex patches in Constantin and Wu [18, 19], Abidi and Danchin [1] and Masmoudi [38], with *low* Besov type regularity in space. In this results one typically gets a bound only in $L^2$ of the form

$$\begin{aligned} \Vert u_\nu (t) - u(t) \Vert _{L^2} \lesssim (\nu t)^\alpha \quad \text {for some} \quad \alpha > 0. \end{aligned}$$

In the case of non-smooth vorticity, the inviscid limit has been investigated by using a Lagrangian stochastic approach in Constantin, Drivas and Elgindi [16], with initial vorticity $\omega _0 \in L^\infty ({\mathbb {T}}^2)$, and in Ciampa, Crippa and Spirito [14], where the initial vorticity $\omega _0 \in L^p({\mathbb {T}}^2)$, $p \in (1, + \infty )$. When the domain has an actual boundary, the zero-viscosity limit is closely related to the validity of the Prandtl equation for the formation of boundary layers. For completeness of the exposition, we mention the work of Sammartino and Caflisch [40, 41] and recent results by Maekawa [37], Constantin, Kukavica and Vicol [17] and Gérard-Varet, Lacave, Nguyen and Rousset [25], with references therein. The inviscid limit has been also investigated in other physical models for complex fluids, see for instance [12] for the 2D incompressible viscoelasticity system.

Our approach is different and it is based on KAM (Kolmogorov–Arnold–Moser) and Normal Form methods for Partial Differential Equations. This fields started from the Nineties, with the pioneering papers of Bourgain [11], Craig and Wayne [20], Kuksin [35], Wayne [44]. We refer to the recent review article [6] for a complete list of references on this topic. In the last years, new techniques have been developed in order to study periodic and quasi-periodic solutions for PDEs arising from fluid dynamics. For the two dimensional water waves equations, we mention Iooss, Plotnikov and Toland [30] for periodic standing waves, [2, 10] for quasi-periodic standing waves and [7, 8, 24] for quasi-periodic traveling wave solutions.

We also recall that the challenging problem of constructing quasi-periodic solutions for the three-dimensional water waves equations is still open. Partial results have been obtained by Iooss and Plotnikov, who proved existence of symmetric and asymmetric *diamond waves* (bi-periodic waves stationary in a moving frame) in [31, 32]. Very recently, KAM techniques have been successfully applied also for the contour dynamics of vortex patches in active scalar equations. The existence of time quasi-periodic solutions have been proved in Berti, Hassainia and Masmoudi [9] for vortex patches of the Euler equations close to Kirchhoff ellipses, in Hmidi and Roulley [29] for the quasi-geostrophic shallow-water equations, in Hassainia, Hmidi and Masmoudi [27] and Gómes-Serrano, Ionescu and Park [26] for generalized surface quasi-geostrophic equations, and in Hassainia and Roulley [28] for vortex patches of the Euler equations close to Rankine vortices in the unit disk. All the aforementioned results concern two-dimensional problems. The quasi-periodic solutions for the 3D Euler equations with time quasi-periodic external force have been constructed in [4] and also extended in [39] for the Navier–Stokes equations in arbitrary dimension, *without* dealing with the zero-viscosity limit. The result of the present paper closes also the gap between these two works in dimension two.

### Main Result

We now state precisely our main result. We look for time-quasi-periodic solutions of (1.1), oscillating with time frequency $\omega$. In particular, we look for solutions which are small perturbations of constant velocity fields $\zeta \in {\mathbb {R}}^2$, namely solutions of the form

$$\begin{aligned} U (t, x ) = \zeta + u (\varphi , x)|_{\varphi = \omega t} \quad \text {with} \quad {\,\text{ div }\,}u = 0, \end{aligned}$$

where the new unknown velocity field $u: {\mathbb {T}}^d \times {\mathbb {T}}^2 \rightarrow {\mathbb {R}}^2$ is a function of $(\varphi ,x)\in {\mathbb {T}}^d\times {\mathbb {T}}^2$. Plugging this ansatz into the equation, one is led to solve

1.2 $$\begin{aligned} {\left\{ \begin{array}{ll} \omega \cdot \partial _\varphi u + \zeta \cdot \nabla u + u \cdot \nabla u - \nu \Delta u + \nabla p = \varepsilon f(\varphi , x) \\ {\,\text{ div }\,}u = 0, \end{array}\right. } \end{aligned}$$

with $p: {\mathbb {T}}^d \times {\mathbb {T}}^2 \rightarrow {\mathbb {R}}$ and $\omega \cdot \partial _\varphi := \sum _{i = 1}^d \omega _i \partial _{\varphi _i}$. According to [4], we shall assume that the forcing term *f* is odd with respect to $(\varphi , x)$, that is

1.3 $$\begin{aligned} f(\varphi , x) = - f(- \varphi , - x), \quad \forall (\varphi , x) \in {\mathbb {T}}^d \times {\mathbb {T}}^2. \end{aligned}$$

It is convenient to work in the well known *vorticity formulation*. We define the scalar vorticity $v(\varphi , x)$ as

$$\begin{aligned} v:= \nabla \times u:= \partial _{x_1} u_2 - \partial _{x_2} u_1 . \end{aligned}$$

Hence, rescaling the variable $v \mapsto \sqrt{\varepsilon } v$ and the small parameter $\varepsilon \mapsto \varepsilon ^2$, Eq. (1.2) is equivalent to

1.4 $$\begin{aligned} {\left\{ \begin{array}{ll} \omega \cdot \partial _\varphi v + \zeta \cdot \nabla v - \nu \Delta v + \varepsilon \Big ( u \cdot \nabla v - F(\varphi , x) \Big ) = 0, \quad F:= \nabla \times f, \\ u = \nabla _\bot \big [(- \Delta )^{- 1} v \big ], \quad \nabla _\bot := (\partial _{x_2}, - \partial _{x_1}), \end{array}\right. } \end{aligned}$$

and $(-\Delta )^{-1}$ is the inverse of the Laplacian, namely the Fourier multiplier with symbol $|\xi |^{-2}$ for $\xi \in {\mathbb {Z}}^2$, $\xi \ne 0$, Since $\int _{{\mathbb {T}}^2} v(\cdot , x)\, \textrm{d} x$ is a prime integral, we shall restrict to the space of zero average in *x*. Then, the pressure is recovered, once the velocity field is known, by the formula $p = \Delta ^{- 1} \big [ \varepsilon {\textrm{div}} f(\omega t, x) - {\,\text{ div }\,}( u \cdot \nabla u ) \big ]$.

For any real $s \ge 0$, we consider the Sobolev spaces $H^s = H^s({\mathbb {T}}^{d + 2})$ of real scalar and vector-valued functions of $(\varphi ,x)$, defined in (2.1), and the Sobolev space of functions with zero space average, defined by

$$\begin{aligned} H^s_0:= \Big \{ u \in H^s: \int _{{\mathbb {T}}^{2}} u(\varphi , x)\, \textrm{d} x= 0 \Big \}. \end{aligned}$$

Furthermore, we introduce the subspaces of $L^2$ of the even and odd functions in $(\varphi ,x)$, respectively:

1.5 $$\begin{aligned} \begin{aligned} X&:= \Big \{ v \in L^2({\mathbb {T}}^{d + 2}): v(\varphi , x) = v(- \varphi , - x) \Big \} \\ Y&:= \Big \{ v \in L^2({\mathbb {T}}^{d + 2}): v(\varphi , x) = - v(- \varphi , - x) \Big \}. \end{aligned} \end{aligned}$$

We first state the result concerning the existence of quasi-periodic solutions of the Euler equation $(\nu = 0)$ for most values of the parameters $(\omega ,\zeta )$ in a fixed bounded open set $\Omega \subset {\mathbb {R}}^d\times {\mathbb {R}}^2$, proved in [4]. The statement is slightly modified for the purposes of this paper.

#### Theorem 1.1

(BaldiMontalto [4]). There exists ${{\overline{S}}}:= {{\overline{S}}}(d) > 0$ such that, for any $S \ge {{\overline{S}}}(d)$, there exists $q:= q(S) > 0$ such that, for every forcing term $f \in {{\mathcal {C}}}^q({\mathbb {T}}^d \times {\mathbb {T}}^2, {\mathbb {R}}^2)$ satisfying (1.3), there exist $\varepsilon _0:= \varepsilon _0(f, S, d) \in (0, 1)$ and $C:= C(f, S, d) > 0$ such that, for every $\varepsilon \in (0, \varepsilon _0)$, the following holds. There exist a ${{\mathcal {C}}}^1$ map

$$\begin{aligned} \begin{aligned}&{\mathbb {R}}^{d + 2} \rightarrow H^S_0({\mathbb {T}}^{d + 2}) \cap Y, \quad \lambda = (\omega , \zeta ) \mapsto v_e(\cdot ; \lambda ), \end{aligned} \end{aligned}$$

and a Borel set $\Omega _\varepsilon \subset \Omega$ of asymptotically full Lebesgue measure, i.e., $\lim _{\varepsilon \rightarrow 0} |\Omega {\setminus } \Omega _\varepsilon | = 0$, such that, for any $\lambda = (\omega , \zeta ) \in \Omega _\varepsilon$, the function $v_e(\cdot ; \lambda )$ is a quasi-periodic solution of the Euler equation

$$\begin{aligned} \omega \cdot \partial _\varphi v_e + \zeta \cdot \nabla v_e + \varepsilon \Big ( u_e \cdot \nabla v_e - F \Big ) = 0, \quad u_e = \nabla _\bot (- \Delta )^{- 1} v_e. \end{aligned}$$

Moreover, there exists a constant $\mathtt a:= \mathtt a(d) \in (0, 1)$ such that $\sup _{\lambda \in {\mathbb {R}}^{d + 2}} \Vert v(\cdot ; \lambda ) \Vert _S \le C \varepsilon ^{\mathtt a}$ and, for any $i = 1, \ldots , d + 2$, $\sup _{\lambda \in {\mathbb {R}}^{d + 2}} \Vert \partial _{\lambda _i} v(\cdot ; \lambda ) \Vert _S \le C \varepsilon ^{\mathtt a}$.

We now are ready to state the main result of this paper. Rougly speaking, we will prove that for any value of the viscosity parameter $\nu > 0$ and for $\varepsilon \ll 1$ small enough, *independent of the viscosity parameter*, the Navier–Stokes Eq. (1.4) admits a quasi-periodic solution $v_\nu (\varphi , x)$ for most of the parameters $\lambda = (\omega , \zeta )$ such that $\Vert v_\nu - v_e \Vert _S = O(\nu )$. This implies that $v_\nu (\omega t, x)$ converges strongly to $v_e(\omega t, x)$, uniformly in $(t, x) \in {\mathbb {R}}\times {\mathbb {T}}^2$, with a rate of convergence $\sup _{(t, x) \in {\mathbb {R}}\times {\mathbb {T}}^2}|v_\nu (\omega t, x) - v_e(\omega t, x)| \lesssim \nu$. To the best of our knowledge, this is the first case in which the inviscid limit is uniform in time for non-trivial, time-dependent solutions. We now give the precise statement of our main theorem.

#### Theorem 1.2

(Singular KAM for 2D Navier–Stokes in the inviscid limit). There exist ${{\overline{s}}}:= {{\overline{s}}}(d)$ and ${{\overline{\mu }}}:= {{\overline{\mu }}}(d) > 0$ such that, for any $s \ge {{\overline{s}}}(d)$, there exists $q:= q(s) > 0$ such that, for every forcing term $f \in {{\mathcal {C}}}^q({\mathbb {T}}^d \times {\mathbb {T}}^2, {\mathbb {R}}^2)$ satisfying (1.3), there exist $\varepsilon _0:= \varepsilon _0(f, s, d) \in (0, 1)$ and $C:= C(f, s, d) > 0$ such that, for every $\varepsilon \in (0, \varepsilon _0)$ and for any value of the viscosity parameter $\nu > 0$, the following holds. Let $v_e(\cdot ; \lambda ) \in H^{s + {{\overline{\mu }}}}_0({\mathbb {T}}^{d + 2}) \cap Y$, $\lambda \in \Omega _\varepsilon$ be the family of solutions of the Euler equation provided by Theorem 1.1. Then, there exists a Borel set ${{\mathcal {O}}}_\varepsilon \subseteq \Omega _\varepsilon$, satisfying $\lim _{\varepsilon \rightarrow 0} |{{\mathcal {O}}}_\varepsilon | = |\Omega |$ such that, for any $\lambda = (\omega , \zeta ) \in {{\mathcal {O}}}_\varepsilon$, there exists a unique quasi-periodic solution $v_\nu (\cdot ; \lambda ) \in H^s_0({\mathbb {T}}^{d + 2})$, $\lambda \in {{\mathcal {O}}}_\varepsilon$, of the Navier–Stokes equation

$$\begin{aligned}{} & {} \omega \cdot \partial _\varphi v_\nu + \zeta \cdot \nabla v_\nu \\{} & {} \quad - \nu \Delta v_\nu + \varepsilon \Big (u_\nu \cdot \nabla v_\nu - F(\varphi , x) \Big ) =0, \quad u_\nu = \nabla _\bot [(- \Delta )^{- 1} v_\nu \big ], \end{aligned}$$

satisfying the estimate

$$\begin{aligned} \sup _{\lambda \in {{\mathcal {O}}}_\varepsilon }\Vert v_\nu (\cdot ; \lambda ) - v_e(\cdot ; \lambda ) \Vert _s \lesssim _s \nu . \end{aligned}$$

As a consequence, for any value of the parameter $\lambda \in {{\mathcal {O}}}_\varepsilon$, the quasi-periodic solutions of the Navier Stokes equation $v_\nu$ converge to the ones of the Euler equation $v_e$ in $H^s_0({\mathbb {T}}^{d + 2})$ in the limit $\nu \rightarrow 0$.

From the latter theorem we shall deduce the following corollary which provides a family of quasi-periodic solutions of Navier–Stokes equation converging to solutions of the Euler equation with rate of convergence $O(\nu )$ and uniformly for all times. The result is a direct consequence of the Sobolev embeddings.

#### Corollary 1.3

(Uniform rate of convergence for the inviscid limit). Assume the same hypotheses of Theorem 1.2 and let $s \ge s_0$ large enough, $v_e \in H^{s + {{\overline{\mu }}}}_0({\mathbb {T}}^{d + 2})$, $v_\nu \in H^s_0({\mathbb {T}}^{d + 2})$ and, for $\omega \in {{\mathcal {O}}}_\varepsilon$, let $v_{\nu }^\omega (t, x):= v_\nu (\omega t, x)$, $v_{ e}^\omega (t, x):= v_e(\omega t, x)$ be defined for any $(t, x) \in {\mathbb {R}}\times {\mathbb {T}}^2$. Then, for any $\alpha \in {\mathbb {N}}$, $\beta \in {\mathbb {N}}^2$ with $|\alpha | + |\beta | \le s - (\lfloor \frac{d+2}{2}\rfloor +1)$, one has

$$\begin{aligned} \Vert \partial _t^\alpha \partial _x^\beta (v_{ \nu }^\omega - v_{ e}^\omega ) \Vert _{L^\infty ({\mathbb {R}}\times {\mathbb {T}}^2)} \lesssim _{\alpha , \beta } \nu . \end{aligned}$$

Let us make some remarks on the result.

1. *Vanishing viscosity solutions of the Cauchy problem.* The time quasi-periodic solutions in Theorem 1.2 are slight perturbations of constant velocity fields $\zeta \in {\mathbb {R}}^2$ with frequency vector $\omega \in {\mathbb {R}}^d$ induced by the perturbative forcing term $f(\omega t,x)$. Since they exist only for most values of the parameters $(\omega ,\zeta )$, we obtain equivalently that the Cauchy problem associated with (1.4) (and so of (1.1)) admits a subset of small amplitude initial data of relatively large measure, with elements evolving *for all time*, in a eventually larger but still bounded neighborhood in the Sobolev topology, and whose flows exhibit a uniform vanishing viscosity limit to solutions of the Cauchy problem for the Euler equations with same initial data.
2. *The role of the forcing term.* It is worth to note that the time quasi-periodic external forcing term $F(\omega t,x)$ in (1.1) is independent of the viscosity parameter $\nu >0$. Its presence ensures the existence of the time quasi-periodic Euler solution $v_e$ in Theorem 1.1, while the construction of the viscous correction $v_{\nu }-v_{e}$ does not rely explicitly on it: if one is able to exhibit time quasi-periodic solutions close to constant velocity fields for the free 2D Euler equation, namely (1.4) with $\nu =0$ and $F\equiv 0$, then the ones for the Navier–Stokes equation follow immediately by our scheme. To our knowledge, the only result of existence of time quasi-periodic flows for the free Euler equations on ${\mathbb {T}}^2$ is given by Crouseilles and Faou [21] (extended recently in the 3D case by Enciso, Peralta-Salas and Torres de Lizaur [22]), where the solutions are searched based on a prescribed stationary shear flow, locally constant around finitely many points, and propagate in time in the orthogonal direction to the shear flow. Due to the nature of their solutions, the non-resonant frequencies are prescribed as well and therefore there are no small divisors issues involved.

### Strategy and Main Ideas of the Proof

In order to prove Theorem 1.2, we have to construct a solution of the Navier–Stokes Eq. (1.4) which is a correction of order $O(\nu )$ of the solution $v_e$ of the Euler equation (provided in Theorem 1.1 of [4]). Roughly speaking, the difficult point is the following. There are two smallness parameters which are $\varepsilon$, the size of the Euler solution, and $\nu$, the size of the viscosity. If one tries to construct small solutions of the Navier–Stokes equation by using a standard fixed point argument, one immediately notes that a smallness condition of the form $\varepsilon \nu ^{- 1} \ll 1$ is needed and clearly this is not enough to pass to the inviscid limit as $\nu \rightarrow 0$. The key point is to have a smallness condition on $\varepsilon$ which is independent of $\nu$ in such a way that one can pass to the limit as the viscosity $\nu \rightarrow 0$. We can summarize the construction into three main steps:

1. Analysis of the linearized Navier–Stokes equation at the Euler solution and estimates for the inverse operators;
2. Construction of the first order approximation for the viscous solution up to errors of order $O(\nu ^2)$;
3. A fixed point argument around the approximated viscous solution leading to the desired full solution of the Navier–Stokes equation.

**Inversion of the linearized operator at the Euler solution.** The essential ingredient is to analyze the linearized Navier–Stokes operator at the Euler solution $u_e$, namely one has to linearize (1.4) at the Euler solution $u_e(\varphi , x)$. This leads to study a linear operator of the form

1.6 $$\begin{aligned} \begin{aligned} {{\mathcal {L}}}_\nu&: = {{\mathcal {L}}}_e - \nu \Delta , \quad , \\ {{\mathcal {L}}}_e&:= \omega \cdot \partial _\varphi + \big ( \zeta + \varepsilon a(\varphi , x)\big ) \cdot \nabla + \varepsilon {{\mathcal {R}}}, \quad a(\varphi , x):= u_e(\varphi , x), \\&\quad \text {where} \quad {{\mathcal {R}}}: h(\varphi , x) \mapsto \nabla _\bot (- \Delta )^{- 1} h(\varphi , x) \cdot \nabla u_e(\varphi , x) \end{aligned} \end{aligned}$$

is a pseudo-differential operator of order $- 1$. Note that the linear operator ${{\mathcal {L}}}_e$ is obtained by linearizing the Euler equation at the solution with vorticity $v_e(\varphi , x)$. If one tries to implement a naive approach by directly using Neumann series to invert the linear operator ${{\mathcal {L}}}_\nu$, one has to require that $\varepsilon \nu ^{- 1} \ll 1$, which is not enough to pass to the limit as $\nu \rightarrow 0$. To overcome this issue, we first implement the normal form procedure developed in [4, 5] to reduce the Euler operator ${\mathcal {L}}_{e}$ to a diagonal, constant coefficients one, generating an unbounded correction to the viscous term $-\nu \Delta$ of size $O(\varepsilon \nu )$. More precisely, for most values of the parameters $(\omega , \zeta )$ and for $\varepsilon \ll 1$ small enough and *independent of* $\nu$, we construct a bounded, invertible transformation $\Phi : H^s_0 \rightarrow H^s_0$ such that

1.7 $$\begin{aligned} {{\mathcal {L}}}_{\infty , \nu }:= \Phi ^{- 1} {{\mathcal {L}}}_\nu \Phi = {{\mathcal {D}}}_\infty - \nu \Delta + {{\mathcal {R}}}_{\infty ,\nu } \end{aligned}$$

where ${{\mathcal {D}}}_\infty$ and ${{\mathcal {R}}}_{\infty , \nu }$ have the following properties. ${{\mathcal {D}}}_\infty$ is a diagonal operator of the form

1.8 $$\begin{aligned} \begin{aligned} {{\mathcal {D}}}_\infty&:= {\textrm{diag}}_{(\ell , j) \in {\mathbb {Z}}^d \times ({\mathbb {Z}}^2 \setminus \{ 0 \})}\, \mu _\infty (\ell , j), \\ \mu _\infty (\ell , j)&:= {{\textrm{i}}}(\omega \cdot \ell + \zeta \cdot j + r_j^\infty ), \\&\quad \text {with }\quad |r_j^\infty | \lesssim \varepsilon |j|^{- 1} \quad \forall \, j \in {\mathbb {Z}}^2 \setminus \{ 0 \}. \end{aligned} \end{aligned}$$

The remainder term ${{\mathcal {R}}}_{\infty , \nu }$ is an unbounded operator of order two and it satisfies an estimate of the form

1.9 $$\begin{aligned} \Vert (- \Delta )^{- 1} {{\mathcal {R}}}_{\infty , \nu }\Vert _{{{\mathcal {B}}}(H^s)} \lesssim _s \varepsilon \nu \end{aligned}$$

where we denote by ${{\mathcal {B}}}(H^s)$, the space of bounded linear operators on $H^s$. The estimate (1.9) is the key ingredient to invert the operator ${{\mathcal {L}}}_{\infty , \nu }$ in (1.7) with a smallness condition on $\varepsilon$ uniform with respect to the viscosity parameter $\nu > 0$. It is also crucial to exploit the reversibility structure which is a consequence of the fact that the solutions $v_e(\varphi , x)$ of the Euler equation are odd with respect to $(\varphi , x)$. This ensures that, for any $\ell \in {\mathbb {Z}}^d$, $j \in {\mathbb {Z}}^2 {\setminus } \{ 0 \}$, the eigenvalues $\mu _\infty (\ell , j)$ of the diagonal operator ${{\mathcal {D}}}_\infty$ in (1.8) are purely imaginary (namely, the corrections $r_j^\infty$ are real). An important consequence is that the diagonal operator ${{\mathcal {D}}}_\infty - \nu \Delta$ is invertible and *gains two space derivatives* with an estimate for its inverse of order $O(\nu ^{- 1})$. Indeed, the eigenvalues of ${{\mathcal {D}}}_\infty - \nu \Delta$ are ${{\textrm{i}}}(\omega \cdot \ell + \zeta \cdot j + r_j^\infty ) + \nu |j|^2$, with $(\ell , j) \in {\mathbb {Z}}^d \times ({\mathbb {Z}}^2 {\setminus } \{ 0 \})$, and, since $\omega \cdot \ell + \zeta \cdot j + r_j^\infty$ is real, one gets a lower bound

1.10 $$\begin{aligned} |{{\textrm{i}}}(\omega \cdot \ell + \zeta \cdot j + r_j^\infty ) + \nu |j|^2| \ge \nu |j|^2, \end{aligned}$$

implying that ${{\mathcal {D}}}_\infty - \nu \Delta$ is invertible with inverse which gain two space derivatives, namely

$$\begin{aligned} \Vert ({{\mathcal {D}}}_\infty - \nu \Delta )^{- 1} (- \Delta )\Vert _{{{\mathcal {B}}}(H^s)} \lesssim \nu ^{- 1}. \end{aligned}$$

Thus, on one hand, $({{\mathcal {D}}}_\infty - \nu \Delta )^{- 1}$ gains two space derivatives, compensating the loss of two space derivatives of the remainder ${{\mathcal {R}}}_{\infty , \nu }$. On the other hand, the norm of $({{\mathcal {D}}}_\infty - \nu \Delta )^{- 1}$ explodes as $\nu ^{- 1}$ as $\nu \rightarrow 0$, but this is compensated by the fact that ${{\mathcal {R}}}_{\infty , \nu }$ is of order $O(\varepsilon \nu )$. Therefore, recalling (1.9), one gets a bound

$$\begin{aligned} \Vert ({{\mathcal {D}}}_\infty - \nu \Delta )^{- 1} {{\mathcal {R}}}_{\infty , \nu }\Vert _{{{\mathcal {B}}}(H^s)} \lesssim \nu ^{- 1} (\varepsilon \nu ) \lesssim \varepsilon . \end{aligned}$$

Hence, by Neumann series, for $\varepsilon \ll 1$ small enough and independent of $\nu$, the operator ${{\mathcal {L}}}_{\infty , \nu }$ is invertible and gains two space derivatives, with estimate $\Vert {{\mathcal {L}}}_{\infty , \nu }^{- 1} (- \Delta ) \Vert _{{{\mathcal {B}}}(H^s)} \lesssim _s \nu ^{- 1}$. By (1.7), we deduce that ${{\mathcal {L}}}_\nu$ is invertible as well and satisfies, for $\varepsilon \ll 1$ and for any $\nu > 0$,

1.11 $$\begin{aligned} \Vert {{\mathcal {L}}}_\nu ^{- 1} (- \Delta ) \Vert _{{{\mathcal {B}}}(H^s)} \lesssim _s \nu ^{- 1}. \end{aligned}$$

**First order approximation for the viscosity quasi-periodic solution and fixed point argument.** Once we have a good knowledge for properly inverting the operators ${\mathcal {L}}_{\nu }$ and ${\mathcal {L}}_{\varepsilon }$, we are ready to construct quasi-periodic solutions of the Navier–Stokes equation converging to the Euler solution $u_e$ as $\nu \rightarrow 0$. First, we define an approximate solution $v_{app} = v_e + \nu v_1$ which solves Eq. (1.4) up to order $O(\nu ^2)$. By making a formal expansion with respect to the viscosity parameter $\nu$, we ask $v_{e}$ to solve the equation at the zeroth order $O(\nu ^0)$, namely the Euler equation, whose existence is provided by Theorem 1.1, and $v_1$ to solve the linear equation at the first order $O(\nu )$, that is ${\mathcal {L}}_{e} v_1 = \Delta v_{e}$. This procedure leads to a loss of regularity due to the presence of * small divisors*, appearing in the inversion of the linearized Euler operator ${{\mathcal {L}}}_e$ in (1.6), which satisfies an estimate of the form $\Vert {{\mathcal {L}}}_e^{- 1} h \Vert _s \lesssim _s \Vert h \Vert _{s + \tau }$ for some $\tau \gg 0$ large enough. On the other hand, this is not a problem in our scheme since it appears only twice: first, in the construction of the quasi-periodic solution $v_e$, but it has already been dealt in Theorem 1.1; second, in the definition indeed of $v_1$. We overcome this issue by requiring $v_e$ to be sufficiently regular. The final step to prove Theorem 1.2 is to implement a fixed point argument for constructing solutions of the form $v = v_e + \nu v_1 + \psi$, where the quasi-periodic correction $\psi$ lies in the ball $\Vert \psi \Vert _s \le \nu$. It is crucial here that $v_e + \nu v_1$ is an approximate solution up to order $O(\nu ^2)$: indeed, the fixed point iteration asks to invert the linearized operator at the Euler solution ${\mathcal {L}}_{\nu }$, which has a bound of order $O(\nu ^{- 1})$ (recall (1.11)), and in this way the new term ends up to be of order $O(\nu )$ as desired. The good news here is that, at this stage, no small divisors are involved and, consequently, no losses of derivatives, which would have made the fixed point argument not applicable otherwise.

*2D vs. 3D.* It is worth concluding this introduction by making some comments on the 3D case, which is not covered by the method developed in this paper. In the present paper, we construct global in time quasi-periodic solutions for the *two dimensional* Navier–Stokes equations converging uniformly in time to global quasi-periodic solutions of the two-dimensional forced Euler equation. The three-dimensional case is much harder. The biggest obstacle is that the reversible structure is not enough to deduce that the spectrum of the linearized Euler operator after the KAM reducibility scheme is purely imaginary. Indeed, as in [4], the reduced Euler operator ${{{\mathcal {D}}}}_\infty$ is a $3 \times 3$ block diagonal operator ${{{\mathcal {D}}}}_\infty = {\textrm{diag}}_{j \in {\mathbb {Z}}^3 {\setminus } \{ 0 \}} D_\infty (j)$ where the $3 \times 3$ matrix $D_\infty (j)$ has the form $D_\infty (j) = {{\textrm{i}}}\zeta \cdot j {\textrm{Id}} + \varepsilon R_\infty (j)$ for $j \in {\mathbb {Z}}^3 \setminus \{ 0 \}$. This block matrix could have eigenvalues $\mu _1(j), \mu _2(j), \mu _3(j)$ of the form $\mu _i(j) = {{\textrm{i}}}\zeta \cdot j + \varepsilon r_i(j)$, $i = 1,2,3$, with real part different from zero, in particular with ${\textrm{Re}}(r_i(j)) \ne 0$ for some $i = 1,2,3$. This seems to be an obstruction to get a lower bound like (1.10) with a gain of two space derivatives, which holds uniformly in $\varepsilon$ and for any value of the viscosity parameter. More precisely, one gets a lower bound on the eigenvalues of the form

$$\begin{aligned} |{{\textrm{i}}}\omega \cdot \ell + \mu _i(j) + \nu |j|^2| \ge |\varepsilon \textrm{Re}(r_i(j)) + \nu |j|^2 |. \end{aligned}$$

It is therefore not clear how to bound the latter quantity by $C \nu |j|^2$ without linking $\varepsilon$ and $\nu$ which prevent to pass to the limit as $\nu \rightarrow 0$ (independently of $\varepsilon$).

**Outline of the paper.** The rest of this paper is organized as follows. In Sect. 2 we introduce the functional setting and some general lemmata that we will employ in the other sections. In Sect. 3 we formulate the nonlinear functional ${\mathcal {F}}_{\nu }$ in (3.1), whose zeroes correspond to quasi-periodic solutions of Eq. (1.4), together with the linearized operators that we have to study. In Sect. 4 we implement the normal form method on the linearized Euler and Navier–Stokes operators operator ${\mathcal {L}}_{\varepsilon }, {\mathcal {L}}_\nu$ in (1.6): first, we regularize to constant coefficients the highest and lower orders, in Sects. 4.1 and 4.2 up to sufficiently smoothing orders; then, in Sects. 4.3–4.4 we prove the full KAM reducibility scheme. We shall prove that the normal form transformations conjugate the linearized Navier–Stokes operator to a diagonal one plus a remainder which is unbounded of order two and has size $O(\varepsilon \nu )$. This normal form procedure is uniform w.r. to the viscosity parameter since it requires a smallness condition on $\varepsilon$ which is independent of the viscosity $\nu > 0$. Then, in Sect. 5 we show the invertibility of the operator ${\mathcal {L}}_{\nu }$ (and also ${\mathcal {L}}_e$) that will be used in Sect. 6 for the construction of the first order approximate solution and in Sect. 7 for the fixed point argument. Finally, the proof of Theorem 1.2 is provided in Sect. 8, together with the measure estimates proved in Sect. 8.1.

## Norms and Linear Operators

In this section we collect some general definitions and properties concerning norms and matrix representation of operators which are used in the whole paper.

**Notations.** In the whole paper, the notation $A \lesssim _{s, m} B$ means that $A \le C(s, m) B$ for some constant $C(s, m) > 0$ depending on the Sobolev index *s* and a generic constant *m*. We always omit to write the dependence on *d*, which is the number of frequencies, and $\tau$, which is the constant appearing in the non-resonance conditions (see for instance (4.32)). We often write $u = \textrm{even}(\varphi , x)$ if $u \in X$ and $u = {\textrm{odd}}(\varphi , x)$ if $u \in Y$ (recall (1.5)). For a given Banach space *Z*, we recall that ${\mathcal {B}}(Z)$ denotes the space of bounded operators from *Z* into itself.

### Function Spaces

Let $a: {\mathbb {T}}^d \times {\mathbb {T}}^2 \rightarrow {\mathbb {C}}$, $a = a(\varphi ,x)$, be a function. then, for $s \in {\mathbb {R}}$, its Sobolev norm $\Vert a \Vert _s$ is defined as

$$\begin{aligned} \Vert a \Vert _s^2:= \sum _{(\ell , j) \in {\mathbb {Z}}^d \times {\mathbb {Z}}^2} \langle \ell , j \rangle ^{2s} | {{\widehat{a}}}(\ell ,j) |^2, \quad \ \langle \ell , j \rangle := \max \{ 1, |\ell |, |j| \}, \end{aligned}$$

where ${{\widehat{a}}}(\ell ,j)$ (which are scalars, or vectors, or matrices) are the Fourier coefficients of $a(\varphi ,x)$, namely

$$\begin{aligned} {{\widehat{a}}}(\ell ,j):= \frac{1}{(2\pi )^{d+2}} \int _{{\mathbb {T}}^{d+2}} a(\varphi ,x) e^{- {{\textrm{i}}}(\ell \cdot \varphi + j \cdot x)} \, \textrm{d}\varphi \textrm{d}x. \end{aligned}$$

We denote, for $E = {\mathbb {C}}^n$ or ${\mathbb {R}}^n$,

2.1 $$\begin{aligned} \begin{aligned} H^s&:= H^s_{\varphi ,x}:= H^s({\mathbb {T}}^{d} \times {\mathbb {T}}^2) \\&:= H^s({\mathbb {T}}^{d} \times {\mathbb {T}}^2, E):= \{ u: {\mathbb {T}}^{d} \times {\mathbb {T}}^2 \rightarrow E, \ \Vert u \Vert _s < \infty \}, \end{aligned} \end{aligned}$$

In the paper we use Sobolev norms for (real or complex, scalar- or vector- or matrix-valued) functions $u( \varphi , x; \omega , \zeta )$, $(\varphi ,x) \in {\mathbb {T}}^d \times {\mathbb {T}}^2$, being Lipschitz continuous with respect to the parameters $\lambda :=(\omega ,\zeta ) \in {\mathbb {R}}^{d+2}$. We fix

2.2 $$\begin{aligned} s_0 \ge \lfloor \tfrac{d+2}{2}\rfloor + 2 \end{aligned}$$

once and for all, and define the weighted Sobolev norms in the following way.

#### Definition 2.1

(*Weighted Sobolev norms*). Let $\gamma \in (0,1]$, $\Lambda \subseteq {\mathbb {R}}^{d + 2}$ and $s \ge s_0$. Given a function $u: \Lambda \rightarrow H^s({\mathbb {T}}^d \times {\mathbb {T}}^2)$, $\lambda \mapsto u(\lambda ) = u(\varphi ,x; \lambda )$ that is Lipschitz continuous with respect to $\lambda$, we define its weighted Sobolev norm by

$$\begin{aligned} \Vert u \Vert _{s}^{\textrm{Lip}(\gamma )}:= \Vert u\Vert _{s}^{\sup } + \gamma \,\Vert u\Vert _{s-1}^{\textrm{lip}}, \end{aligned}$$

where

$$\begin{aligned} \Vert u\Vert _{s}^{\sup }:= \sup _{\lambda \in \Lambda } \Vert u(\lambda )\Vert _{s}, \quad \Vert u\Vert _{s}^{\textrm{lip}}:= \sup _{\lambda _1,\lambda _2\in \Lambda \atop \lambda _1\ne \lambda _2} \frac{\Vert u(\lambda _1)-u(\lambda _2)\Vert _{s}}{| \lambda _1-\lambda _2|}. \end{aligned}$$

For *u* independent of $(\varphi ,x)$, we simply denote by $| u |^{k_0,\gamma }:= | u|^{\sup } + \gamma \, | u|^{\textrm{lip}}$.

For any $N>0$, we define the smoothing operators (Fourier truncation)

2.3 $$\begin{aligned} (\Pi _N u)(\varphi ,x):= \sum _{\langle \ell ,j \rangle \le N} {{\widehat{u}}}(\ell , j) e^{{{\textrm{i}}}(\ell \cdot \varphi + j \cdot x)}, \qquad \Pi ^\perp _N:= {\textrm{Id}} - \Pi _N. \end{aligned}$$

#### Lemma 2.2

(Smoothing). The smoothing operators $\Pi _N, \Pi _N^\perp$ satisfy the smoothing estimates

2.4 $$\begin{aligned} \Vert \Pi _N u \Vert _{s}^{\textrm{Lip}(\gamma )}&\le N^a \Vert u \Vert _{s-a}^{\textrm{Lip}(\gamma )}\, , \quad 0 \le a \le s, \end{aligned}$$

2.5 $$\begin{aligned} \Vert \Pi _N^\bot u \Vert _{s}^{\textrm{Lip}(\gamma )}&\le N^{-a} \Vert u \Vert _{s + a}^{\textrm{Lip}(\gamma )}\, , \quad a \ge 0. \end{aligned}$$

#### Lemma 2.3

(Product). For all $s \ge s_0$,

2.6 $$\begin{aligned} \Vert uv \Vert _{s}^{\textrm{Lip}(\gamma )}&\lesssim _s C(s) \Vert u \Vert _{s}^{\textrm{Lip}(\gamma )} \Vert v \Vert _{s_0}^{\textrm{Lip}(\gamma )}+ C(s_0) \Vert u \Vert _{s_0}^{\textrm{Lip}(\gamma )} \Vert v \Vert _{s}^{\textrm{Lip}(\gamma )}\,. \end{aligned}$$

### Matrix Representation of Linear Operators

Let ${{\mathcal {R}}}: L^2({\mathbb {T}}^2) \rightarrow L^2({\mathbb {T}}^2)$ be a linear operator. Such an operator can be represented as

2.7 $$\begin{aligned} {{\mathcal {R}}} u (x):= \sum _{j, j' \in {\mathbb {Z}}^2} {{\mathcal {R}}}_j^{j'}{{\widehat{u}}}(j') e^{{{\textrm{i}}}j \cdot x}, \quad \text {for} \quad u (x) = \sum _{j \in {\mathbb {Z}}^2} {{\widehat{u}}}(j) e^{{{\textrm{i}}}j \cdot x}, \end{aligned}$$

where, for $j, j' \in {\mathbb {Z}}^2$, the matrix element ${{\mathcal {R}}}_j^{j'}$ is defined by

2.8 $$\begin{aligned} {{\mathcal {R}}}_j^{j'}:= \frac{1}{(2\pi )^2} \int _{{\mathbb {T}}^2} {{\mathcal {R}}}[e^{{{\textrm{i}}}j' \cdot x}] e^{- {{\textrm{i}}}j \cdot x}\, \textrm{d} x. \end{aligned}$$

We also consider smooth $\varphi$-dependent families of linear operators ${\mathbb {T}}^d \rightarrow {{\mathcal {B}}} (L^2({\mathbb {T}}^2))$, $\varphi \mapsto {{\mathcal {R}}}(\varphi )$, which we write in Fourier series with respect to $\varphi$ as

2.9 $$\begin{aligned} {{\mathcal {R}}}(\varphi ) = \sum _{\ell \in {\mathbb {Z}}^d} \widehat{{\mathcal {R}}}(\ell ) e^{{{\textrm{i}}}\ell \cdot \varphi }, \quad \widehat{{\mathcal {R}}}(\ell ):= \frac{1}{(2 \pi )^d} \int _{{\mathbb {T}}^d} {{\mathcal {R}}}(\varphi ) e^{- {{\textrm{i}}}\ell \cdot \varphi }\, \textrm{d} \varphi , \quad \ell \in {\mathbb {Z}}^d. \end{aligned}$$

According to (2.8), for any $\ell \in {\mathbb {Z}}^d$, the linear operator $\widehat{{\mathcal {R}}}(\ell ) \in {{\mathcal {B}}} (L^2({\mathbb {T}}^2))$ is identified with the matrix $(\widehat{{\mathcal {R}}}(\ell )_j^{j'})_{j, j' \in {\mathbb {Z}}^2}$. A map ${\mathbb {T}}^d \rightarrow {{\mathcal {B}}} (L^2({\mathbb {T}}^2))$, $\varphi \mapsto {{\mathcal {R}}}(\varphi )$ can be also regarded as a linear operator $L^2({\mathbb {T}}^{d + 2}) \rightarrow L^2({\mathbb {T}}^{d + 2})$ by

2.10 $$\begin{aligned} {{\mathcal {R}}} u(\varphi , x):= \sum _{{\begin{array}{c} \ell , \ell ' \in {\mathbb {Z}}^d \\ j, j' \in {\mathbb {Z}}^2 \end{array}}} \widehat{{\mathcal {R}}}(\ell - \ell ')_j^{j '} {{\widehat{u}}}(\ell ', j') e^{{{\textrm{i}}}(\ell \cdot \varphi + j \cdot x)}, \quad \forall u \in L^2({\mathbb {T}}^{d + 2}). \end{aligned}$$

If the operator ${{\mathcal {R}}}$ is invariant on the space of functions with zero average in *x*, we identify ${{\mathcal {R}}}$ with the matrix

$$\begin{aligned} \Big ( \widehat{{\mathcal {R}}}(\ell )_j^{j'} \Big )_{{\begin{array}{c} j, j' \in {\mathbb {Z}}^2 \setminus \{ 0 \} \\ \ell \in {\mathbb {Z}}^d \end{array}}} \end{aligned}$$

#### Definition 2.4

(*Diagonal operators*) Let ${{\mathcal {R}}}$ be a linear operator as in (2.7)–(2.10). We define ${{\mathcal {D}}}_{{\mathcal {R}}}$ as the operator defined by

$$\begin{aligned} {{\mathcal {D}}}_{{\mathcal {R}}}:= {\textrm{diag}}_{j \in {\mathbb {Z}}^2} \widehat{{\mathcal {R}}}_j^j(0), \quad ({\mathcal {D}}_{{\mathcal {R}}})_j^{j'}(\ell ):= {\left\{ \begin{array}{ll} \widehat{{\mathcal {R}}}_j^{j}(\ell ) &{} \quad j=j', \ \ell =0, \\ 0 &{} \quad \text {otherwise}. \end{array}\right. }. \end{aligned}$$

In particular, we say that ${\mathcal {R}}$ is a *diagonal operator* if ${\mathcal {R}}\equiv {\mathcal {D}}_{{\mathcal {R}}}$.

For the purpose of the Normal form method for the linearized operator in Sect. 4, it is convenient to introduce the following norms that take into account the order and the off-diagonal decay of the matrix elements representing any linear operator on $L^2({\mathbb {T}}^{d+2})$.

#### Definition 2.5

(*Matrix decay norm and the class* ${{\mathcal {O}}}{{\mathcal {P}}}{{\mathcal {M}}}^m_s$). Let $m \in {\mathbb {R}}$, $s \ge s_0$ and ${\mathcal {R}}$ be an operator represented by the matrix in (2.10). We say that ${{\mathcal {R}}}$ belongs to the class ${{\mathcal {O}}}{{\mathcal {P}}}{{\mathcal {M}}}^m_s$ if

2.11 $$\begin{aligned} | {\mathcal {R}}|_{m, s}:= \sup _{j' \in {\mathbb {Z}}^2} \left( \sum _{(\ell ,j) \in {\mathbb {Z}}^{d+2}} \langle \ell , j-j' \rangle ^{2s} | {{\widehat{{\mathcal {R}}}}}(\ell )_j^{j'}|^2 \right) ^{\frac{1}{2}} \langle j' \rangle ^{- m} < \infty \end{aligned}$$

If the operator ${\mathcal {R}}= {\mathcal {R}}(\lambda )$ is Lipschitz with respect to the parameter $\lambda \in \Lambda \subseteq {\mathbb {R}}^{\nu +2}$, we define

2.12 $$\begin{aligned} \begin{aligned} | {{\mathcal {R}}} |_{m, s}^{{\textrm{Lip}}(\gamma )}&:= |{{\mathcal {R}}}|_{m, s}^{\textrm{sup}} + \gamma |{{\mathcal {R}}}|^{\textrm{lip}}_{m, s - 1}, \\ |{{\mathcal {R}}}|_{m, s}^{\textrm{sup}}&:= \sup _{\lambda \in \Lambda } |{{\mathcal {R}}}(\lambda )|_{m, s}, \quad |{{\mathcal {R}}}|_{m, s - 1}^{\textrm{lip}}:= \sup _{{\begin{array}{c} \lambda _1, \lambda _2 \in \Lambda \\ \lambda _1 \ne \lambda _2 \end{array}}} \dfrac{|{{\mathcal {R}}}(\lambda _1) - {{\mathcal {R}}}(\lambda _2)|_{m, s - 1}}{|\lambda _1 - \lambda _2|} \end{aligned}\nonumber \\ \end{aligned}$$

Directly from the latter definition, it follows that

$$\begin{aligned} \begin{aligned} m&\le m' \Longrightarrow {{\mathcal {O}}}{{\mathcal {P}}}{{\mathcal {M}}}^m_s \subseteq {{\mathcal {O}}}{{\mathcal {P}}}{{\mathcal {M}}}^{m'}_s \quad \text {and} \quad |\cdot |_{m', s}^{{\textrm{Lip}}(\gamma )} \le |\cdot |_{m, s}^{{\textrm{Lip}}(\gamma )}, \\ s&\le s' \Longrightarrow {{\mathcal {O}}}{{\mathcal {P}}}{{\mathcal {M}}}^m_{s'} \subseteq {{\mathcal {O}}}{{\mathcal {P}}}{{\mathcal {M}}}^m_s \quad \text {and} \quad | \cdot |_{m, s}^{{\textrm{Lip}}(\gamma )} \le |\cdot |_{m, s'}^{{\textrm{Lip}}(\gamma )}. \end{aligned} \end{aligned}$$

We now state some standard properties of the decay norms that are needed for the reducibility scheme of Sect. 4.3. If $a \in H^s$, $s \ge s_0$, then the multiplication operator ${{\mathcal {M}}}_a: u \mapsto a u$ satisfies

2.13 $$\begin{aligned} {{\mathcal {M}}}_a \in {{\mathcal {O}}}{{\mathcal {P}}}{{\mathcal {M}}}_s^0 \quad \text {and} \quad |{{\mathcal {M}}}_a|_{0, s}^{{\textrm{Lip}}(\gamma )} \lesssim \Vert a \Vert _s^{{\textrm{Lip}}(\gamma )}. \end{aligned}$$

#### Lemma 2.6

(i) Let $s \ge s_0$ and ${{\mathcal {R}}} \in {{\mathcal {O}}}{{\mathcal {P}}}{{\mathcal {M}}}^0_s$. If $\Vert u \Vert _s^{{\textrm{Lip}}(\gamma )} < \infty$, then
  $$\begin{aligned} \Vert {{\mathcal {R}}} u \Vert _s^{{\textrm{Lip}}(\gamma )} \lesssim _{s} |{{\mathcal {R}}}|_{0, s}^{{\textrm{Lip}}(\gamma )} \Vert u \Vert _s^{{\textrm{Lip}}(\gamma )}. \end{aligned}$$
(ii) Let $s \ge s_0$, $m, m' \in {\mathbb {R}}$, and let ${{\mathcal {R}}} \in {{\mathcal {O}}}{{\mathcal {P}}}{{\mathcal {M}}}^m_s$, ${{\mathcal {Q}}} \in {{\mathcal {O}}}{{\mathcal {P}}}{{\mathcal {M}}}^{m'}_{s + |m|}$. Then ${{\mathcal {R}}} {{\mathcal {Q}}} \in {{\mathcal {O}}}{{\mathcal {P}}}{{\mathcal {M}}}^{m + m'}_s$ and
  $$\begin{aligned} |{{\mathcal {R}}}{{\mathcal {Q}}}|_{m + m', s}^{{\textrm{Lip}}(\gamma )} \lesssim _{s, m} |{{\mathcal {R}}}|_{m, s}^{{\textrm{Lip}}(\gamma )} |{{\mathcal {Q}}}|_{m', s_0 + |m|}^{{\textrm{Lip}}(\gamma )} + |{{\mathcal {R}}}|_{m, s_0}^{{\textrm{Lip}}(\gamma )} |{{\mathcal {Q}}}|_{m', s + |m|}^{{\textrm{Lip}}(\gamma )}\ . \end{aligned}$$
(iii) Let $s \ge s_0$ and ${{\mathcal {R}}} \in {{\mathcal {O}}}{{\mathcal {P}}}{{\mathcal {M}}}^0_s$. Then, for any integer $n \ge 1$, ${{\mathcal {R}}}^n \in {{\mathcal {O}}}{{\mathcal {P}}}{{\mathcal {M}}}^0_s$ and there exist constants $C(s_0),C(s) > 0$, independent of *n*, such that
  $$\begin{aligned} \begin{aligned} |{{\mathcal {R}}}^n|_{0, s_0}^{{\textrm{Lip}}(\gamma )}&\le C(s_0)^{n - 1} \big (|{{\mathcal {R}}}|_{0, s_0}^{{\textrm{Lip}}(\gamma )}\big )^{n}, \\ |{{\mathcal {R}}}^n|_{0, s}^{{\textrm{Lip}}(\gamma )}&\le n\,C(s)^{n - 1} \big (C(s_0)|{{\mathcal {R}}}|_{0, s_0}^{{\textrm{Lip}}(\gamma )}\big )^{n - 1} |{{\mathcal {R}}}|_{0, s}^{{\textrm{Lip}}(\gamma )}; \end{aligned} \end{aligned}$$
(iv) Let $s \ge s_0$, $m \ge 0$ and ${{\mathcal {R}}} \in {{\mathcal {O}}}{{\mathcal {P}}}{{\mathcal {M}}}^{- m}_s$. Then there exists $\delta (s) \in (0, 1)$ small enough such that, if $|{{\mathcal {R}}}|_{- m, s_0}^{{\textrm{Lip}}(\gamma )} \le \delta (s)$, then the map $\Phi = {\textrm{Id}} + {{\mathcal {R}}}$ is invertible and the inverse satisfies the estimate
  $$\begin{aligned} |\Phi ^{- 1} - {\textrm{Id}}|_{- m, s}^{{\textrm{Lip}}(\gamma )} \lesssim _{s, m} |{{\mathcal {R}}}|_{- m, s}^{{\textrm{Lip}}(\gamma )}. \end{aligned}$$
(v) Let $s \ge s_0$, $m \in {\mathbb {R}}$ and ${{\mathcal {R}}} \in {{\mathcal {O}}}{{\mathcal {P}}}{{\mathcal {M}}}^m_s$. Let ${{\mathcal {D}}}_{{\mathcal {R}}}$ be the diagonal operator as in Definition 2.4. Then ${{\mathcal {D}}}_{{\mathcal {R}}} \in {{\mathcal {O}}}{{\mathcal {P}}}{{\mathcal {M}}}^m_s$ and $|{{\mathcal {D}}}_{{\mathcal {R}}}|_{m, s}^{{\textrm{Lip}}(\gamma )} \lesssim |{{\mathcal {R}}}|_{m, s}^{{\textrm{Lip}}(\gamma )}$. As a consequence,
  $$\begin{aligned} | \widehat{{\mathcal {R}}}_j^j(0) |^{{\textrm{Lip}}(\gamma )} \lesssim \langle j \rangle ^m|{{\mathcal {R}}}|_{s_0}^{{\textrm{Lip}}(\gamma )}. \end{aligned}$$

#### Proof

(i), (ii) The proofs of the first two items use similar arguments. We only prove item (ii). We start by assuming that both ${\mathcal {R}}$ and ${\mathcal {Q}}$ do not depend on the parameter $\lambda$. The matrix elements for the composition operator ${\mathcal {R}}{\mathcal {Q}}$ follow the rule

$$\begin{aligned} \widehat{{\mathcal {R}}{\mathcal {Q}}}(\ell )_j^{j'} = \sum _{(k, i) \in {\mathbb {Z}}^{d + 2}} {\widehat{{\mathcal {R}}}}(\ell - k)_{j}^{i} {\widehat{{\mathcal {Q}}}}(k)_{i}^{j'}. \end{aligned}$$

Using that $\langle \ell , j - j' \rangle ^s \lesssim _s \langle \ell - k, j - i \rangle ^s + \langle k, i - j' \rangle ^s$, one gets

2.14 $$\begin{aligned} \sum _{{\begin{array}{c} \ell \in {\mathbb {Z}}^d \\ j\in {\mathbb {Z}}^2 \end{array}}} \langle \ell , j - j' \rangle ^{2 s} | \widehat{{\mathcal {R}}{\mathcal {Q}}}(\ell )_j^{j'}|^2 \langle j' \rangle ^{ - 2 (m + m')}&\lesssim _s (A) + (B) \,, \end{aligned}$$

where

$$\begin{aligned} (A)&:= \sum _{{\begin{array}{c} \ell \in {\mathbb {Z}}^d \\ j \in {\mathbb {Z}}^2 \end{array}}} \Big ( \sum _{{\begin{array}{c} k \in {\mathbb {Z}}^d \\ i \in {\mathbb {Z}}^2 \end{array}}} \langle \ell - k, j - i \rangle ^{s} |\widehat{{\mathcal {R}}}_j^{i}(\ell - k) | | \widehat{{\mathcal {Q}}}_{i}^{j'}(k) | \Big )^2 \langle j' \rangle ^{- 2(m + m')}\,, \\ (B)&:= \sum _{{\begin{array}{c} \ell \in {\mathbb {Z}}^d \\ j \in {\mathbb {Z}}^2 \end{array}}} \Big ( \sum _{{\begin{array}{c} k \in {\mathbb {Z}}^d \\ i \in {\mathbb {Z}}^2 \end{array}}} \langle k , i - j' \rangle ^{s} |\widehat{{\mathcal {R}}}_j^{i}(\ell - k) | | \widehat{{\mathcal {Q}}}_{i}^{j'}(k) | \Big )^2\langle j' \rangle ^{- 2(m + m')} \,. \end{aligned}$$

We start with estimating (*A*). By the elementary inequality $\langle i \rangle ^m\langle j' \rangle ^{- m} \lesssim _m \langle j' - i \rangle ^{|m|}$, the Cauchy-Schwartz inequality and having the series $\sum _{k \in {\mathbb {Z}}^d, i \in {\mathbb {Z}}^2} \langle k, i - j'\rangle ^{- 2 s_0} = C(s_0)<\infty$, one has

$$\begin{aligned} \begin{aligned} (A)&\lesssim _{s,m} \sum _{{\begin{array}{c} k \in {\mathbb {Z}}^d \\ i \in {\mathbb {Z}}^2 \end{array}}} \langle k, i - j' \rangle ^{2 (s_0 + |m|)}| \widehat{{\mathcal {Q}}}_{i}^{j'}(k) |^2 \langle j' \rangle ^{- 2 m'} \sum _{{\begin{array}{c} \ell \in {\mathbb {Z}}^d \\ j \in {\mathbb {Z}}^2 \end{array}}} \langle \ell - k, j - i \rangle ^{ 2s} |\widehat{{\mathcal {R}}}_j^{i}(\ell - k) |^2 \langle i \rangle ^{- 2 m}\\&{\mathop {\lesssim _{s,m}}\limits ^{2.11}} |{{\mathcal {Q}}}|_{s_0 + |m|, m'}^2 |{{\mathcal {R}}}|_{s, m}^2 \end{aligned} \end{aligned}$$

By similar arguments, one gets $(B) \lesssim _m |{{\mathcal {Q}}}|_{s + |m|, m'}^2 |{{\mathcal {R}}}|_{s_0, m}^2$ and hence the claimed estimate follows by taking the supremum over $j' \in {\mathbb {Z}}^2$ in (2.14). If we reintroduce the dependence on the parameter $\lambda$, the estimate for the Lipschitz seminorm follows as usual by taking two parameters $\lambda _1, \lambda _2$ and writing ${{\mathcal {R}}}(\lambda _1) {{\mathcal {Q}}}(\lambda _1) - {{\mathcal {R}}}(\lambda _2) {{\mathcal {Q}}}(\lambda _2) = ({{\mathcal {R}}}(\lambda _1) - {{\mathcal {R}}}(\lambda _2)) {{\mathcal {Q}}}(\lambda _1) + {{\mathcal {R}}}(\lambda _2) ({{\mathcal {Q}}}(\lambda _1) - {{\mathcal {Q}}}(\lambda _2))$.

(iii) The claim follows by an induction argument and item (ii).

(iv) The claim follows by a Neumann series argument, together with item (iii).

(v) The claims are a direct consequence of the definition of the matrix decay norm in Definition 2.5. $\square$

We recall the definition of the set of the Diophantine vectors in a bounded, measurable set $\Lambda \subset {\mathbb {R}}^{d + 2}$. Given $\gamma , \tau > 0$, we define

2.15 $$\begin{aligned} \Lambda (\gamma ,\tau ):= \big \{ (\omega ,\zeta )\in \Lambda :\, |\omega \cdot \ell +\zeta \cdot j| \ge \frac{\gamma }{|(\ell ,j)|^{\tau }}, \ \forall \,(\ell ,j)\in {\mathbb {Z}}^{d+2}\setminus \{0\} \big \},\nonumber \\ \end{aligned}$$

where $|(\ell ,j)|:=|\ell |+|j|$ for any $\ell \in {\mathbb {Z}}^d$, $j\in {\mathbb {Z}}^2$.

#### Lemma 2.7

(Homological equation). Let $\Lambda \ni \lambda =(\omega ,\zeta )\mapsto {{\mathcal {R}}}(\lambda )$ be a Lipschitz family of linear operators in ${{\mathcal {O}}}{{\mathcal {P}}}{{\mathcal {M}}}^m_{s + 2 \tau + 1}$. Then, for any $\lambda \in \Lambda (\gamma , \tau )$, there exists a solution $\Psi = \Psi (\lambda ) \in {{\mathcal {O}}}{{\mathcal {P}}}{{\mathcal {M}}}^m_{s}$ of the equation

2.16 $$\begin{aligned} \omega \cdot \partial _\varphi \Psi + [\zeta \cdot \nabla , \Psi ] + {{\mathcal {R}}} = {{\mathcal {D}}}_{{\mathcal {R}}} \end{aligned}$$

satisfying the estimate $|\Psi |_{m, s}^{{\textrm{Lip}}(\gamma )} \lesssim \gamma ^{- 1} |{{\mathcal {R}}}|_{m, s + 2 \tau + 1}^{{\textrm{Lip}}(\gamma )}$. Moreover, if ${{\mathcal {R}}}$ is invariant on the space of zero average functions, also $\Psi$ is invariant on the space of zero average functions.

#### Proof

By the matrix representation (2.9), (2.10), Eq. (2.16) is equivalent to

$$\begin{aligned} {{\textrm{i}}}\big (\omega \cdot \ell + \zeta \cdot (j - j') \big ) {{\widehat{\Psi }}}_j^{j'}(\ell ) + \widehat{{\mathcal {R}}}(\ell )_j^{j'} = 0 \end{aligned}$$

for any $(\ell , j, j') \in {\mathbb {Z}}^d \times {\mathbb {Z}}^2 \times {\mathbb {Z}}^2$ with $(\ell , j, j') \ne (0, j, j)$. We then define $\Psi$ as

$$\begin{aligned} {{\widehat{\Psi }}}(\ell )_j^{j'}:= {\left\{ \begin{array}{ll} - \dfrac{\widehat{{\mathcal {R}}}(\ell )_j^{j'}}{{{\textrm{i}}}\big (\omega \cdot \ell + \zeta \cdot (j - j') \big )} &{}\quad \text {if} \quad (\ell , j, j') \ne (0, j, j), \\ 0 &{}\quad \text {otherwise.} \end{array}\right. } \end{aligned}$$

Since $\lambda = (\omega , \zeta ) \in \Lambda (\gamma , \tau )$, by (2.15), one has that

$$\begin{aligned} |{{\widehat{\Psi }}}(\ell )_j^{j'}| \le \gamma ^{- 1} \langle \ell , j - j' \rangle ^\tau |\widehat{{\mathcal {R}}}(\ell )_j^{j'}|. \end{aligned}$$

The latter estimate, together with Definition 2.11, implies that

2.17 $$\begin{aligned} |\Psi |_{m, s} \lesssim \gamma ^{- 1} |{{\mathcal {R}}}|_{m, s + \tau }. \end{aligned}$$

We prove now the Lipschitz estimate. Let $\lambda _1, \lambda _2 \in \Lambda (\gamma , \tau )$. A direct computation shows that

$$\begin{aligned} \begin{aligned} |{{\widehat{\Psi }}}(\ell )_j^{j'}(\lambda _1) - {{\widehat{\Psi }}}(\ell )_j^{j'}(\lambda _2)|&\lesssim \gamma ^{- 1} \langle \ell , j - j' \rangle ^\tau |\widehat{{\mathcal {R}}}(\ell )_j^{j'} (\lambda _1) - \widehat{{\mathcal {R}}}(\ell )_j^{j'}(\lambda _2)| \\&\quad + \gamma ^{- 2} \langle \ell , j - j' \rangle ^{2 \tau + 1} |\widehat{{\mathcal {R}}}(\ell )_j^{j'}(\lambda _2)| |\lambda _1 - \lambda _2|. \end{aligned} \end{aligned}$$

Hence by recalling (2.11), (2.12) one obtains that

2.18 $$\begin{aligned} |\Psi |_{m, s}^{\textrm{lip}} \lesssim \gamma ^{- 1} |{{\mathcal {R}}}|_{m, s + \tau }^{\textrm{lip}} + \gamma ^{- 2} |{{\mathcal {R}}}|^{\textrm{sup}}_{m, s + 2 \tau + 1}. \end{aligned}$$

The estimates (2.17), (2.18) imply the claimed bound $|\Psi |_{m, s}^{{\textrm{Lip}}(\gamma )} \lesssim \gamma ^{- 1} |{{\mathcal {R}}}|_{m, s + 2 \tau + 1}^{{\textrm{Lip}}(\gamma )}$. $\square$

For $N > 0$, we define the operators $\Pi _N {{\mathcal {R}}}$ and $\Pi _N^\perp {\mathcal {R}}$ by means of their matrix representation as follows:

2.19 $$\begin{aligned} (\widehat{\Pi _N {{\mathcal {R}}}})_{j}^{j'}(\ell ):= {\left\{ \begin{array}{ll} \widehat{{\mathcal {R}}}_j^{j'}(\ell ) &{} \quad \text {if } |\ell |, |j - j'| \le N, \\ 0 &{} \quad \text {otherwise}, \end{array}\right. } \qquad \ \Pi _N^\bot {{\mathcal {R}}}:= {{\mathcal {R}}} - \Pi _N {{\mathcal {R}}}.\nonumber \\ \end{aligned}$$

#### Lemma 2.8

For all $s, \alpha \ge 0$, $m \in {\mathbb {R}}$, one has $|\Pi _N {{\mathcal {R}}}|_{m, s + \alpha }^{{\textrm{Lip}}(\gamma )} \le N^\alpha |{{\mathcal {R}}}|_{m, s}^{{\textrm{Lip}}(\gamma )}$ and $|\Pi _N^\bot {{\mathcal {R}}}|_{m, s}^{{\textrm{Lip}}(\gamma )} \le N^{- \alpha } |{{\mathcal {R}}}|_{m, s + \alpha }^{{\textrm{Lip}}(\gamma )}$.

#### Proof

The claims follow directly from (2.11) and (2.19). $\square$

We also define the projection $\Pi _0$ on the space of zero average functions as

$$\begin{aligned} \Pi _0 h:= \frac{1}{(2 \pi )^{ 2}} \int _{{\mathbb {T}}^{ 2}} h(\varphi , x)\, \textrm{d} x, \qquad \Pi _0^\bot := {\textrm{Id}} - \Pi _0. \end{aligned}$$

In particular, for any $m, s \ge 0$,

2.20 $$\begin{aligned} |\Pi _0^\bot |_{0, s} \le 1, \quad |\Pi _0|_{- m, s} \lesssim _{m} 1. \end{aligned}$$

We finally mention the elementary properties of the Laplacian operator $- \Delta$ and its inverse $(- \Delta )^{- 1}$ acting on functions with zero average in *x*:

$$\begin{aligned} - \Delta u(x) = \sum _{\xi \ne 0} |\xi |^2 {{\widehat{u}}}(\xi ) e^{{{\textrm{i}}}x \cdot \xi }, \quad (- \Delta )^{- 1} u(x) = \sum _{\xi \in {\mathbb {Z}}^2 \setminus \{ 0 \}} \frac{1}{|\xi |^2} {{\widehat{u}}}(\xi ) e^{{{\textrm{i}}}x \cdot \xi }. \end{aligned}$$

By Definition 2.5, one easily verifies, for any $s\ge 0$,

2.21 $$\begin{aligned} |- \Delta |_{2, s} \le 1, \quad |(- \Delta )^{- 1}|_{- 2, s} \le 1. \end{aligned}$$

### Real and Reversible Operators

We recall the notation introduced in (1.5), that is, for any function $u(\varphi ,x)$, we write $u \in X$ when $u = \text {even}(\varphi ,x)$ and $u \in Y$ when $u = \text {odd}(\varphi ,x)$.

#### Definition 2.9

(i) We say that a linear operator $\Phi$ is *reversible* if $\Phi : X \rightarrow Y$ and $\Phi : Y \rightarrow X$. We say that $\Phi$ is *reversibility preserving* if $\Phi : X \rightarrow X$ and $\Phi : Y \rightarrow Y$.
(ii) We say that an operator $\Phi : L^2({\mathbb {T}}^2) \rightarrow L^2({\mathbb {T}}^2)$ is *real* if $\Phi (u)$ is real valued for any *u* real valued.

It is convenient to reformulate real and reversibility properties of linear operators in terms of their matrix representations.

#### Lemma 2.10

A linear operator ${{\mathcal {R}}}$ is:

(i) real if and only if $\widehat{{\mathcal {R}}}_{j}^{j'}(\ell ) = \overline{\widehat{{\mathcal {R}}}_{- j}^{- j'}(- \ell )}$ for all $\ell \in {\mathbb {Z}}^d$, $j, j' \in {\mathbb {Z}}^2$;
(ii) reversible if and only if $\widehat{{\mathcal {R}}}_j^{j'}(\ell ) = - \widehat{{\mathcal {R}}}_{- j}^{- j'}(- \ell )$ for all $\ell \in {\mathbb {Z}}^d$, $j, j' \in {\mathbb {Z}}^2$;
(iii) reversibility preserving if and only if $\widehat{{\mathcal {R}}}_j^{j'}(\ell ) = \widehat{{\mathcal {R}}}_{- j}^{- j'}(- \ell )$ for all $\ell \in {\mathbb {Z}}^d$, $j, j' \in {\mathbb {Z}}^2$.

## The Nonlinear Functional and the Linearized Navier–Stokes Operator at the Euler Solution

We shall show the existence of solutions of (1.4) by finding zeroes of the nonlinear operator ${{\mathcal {F}}}_\nu : H^{s + 2}_0({\mathbb {T}}^{d + 2}) \rightarrow H^s_0({\mathbb {T}}^{d + 2})$ defined by

3.1 $$\begin{aligned} \begin{aligned} {{\mathcal {F}}}_\nu (v)&:= \omega \cdot \partial _\varphi v + \zeta \cdot \nabla v - \nu \Delta v + \varepsilon \big (\Pi _0^\bot \big [ \nabla _\bot (- \Delta )^{- 1} v \cdot \nabla v \big ] - F(\varphi , x) \big ), \end{aligned}\nonumber \\ \end{aligned}$$

with $\nabla _\bot$ as in (1.4), and, without loss of generality, $F = \nabla \times f$ has zero average in space, namely

$$\begin{aligned} \int _{{\mathbb {T}}^2} F(\varphi , x)\, \textrm{d}x = 0, \quad \forall \,\varphi \in {\mathbb {T}}^d. \end{aligned}$$

We consider parameters $(\omega , \zeta )$ in a bounded open set $\Omega \subset {\mathbb {R}}^d \times {\mathbb {R}}^2$; we will use such parameters along the proof in order to impose appropriate non-resonance conditions.

In this section and Sect. 4 we assume the following ansatz, which is implied by Theorem 1.1: there exists $S \gg 0$ large enough such that $v_e(\cdot ; \lambda ) \in H^{S}_0({\mathbb {T}}^d \times {\mathbb {T}}^2)$, $\lambda \in \Omega _\varepsilon$, is a solution of the Euler equation satisfying

3.2 $$\begin{aligned} \Vert v_e \Vert _{S}^{{\textrm{Lip}}(\gamma )} \lesssim _S \varepsilon ^{\mathtt a} \ll 1, \qquad \mathtt a \in (0, 1), \quad S > {{\overline{S}}} \end{aligned}$$

where ${{\overline{S}}}:= {{\overline{S}}}(d)$ is the minimal regularity threshold for the existence of quasi-periodic solutions of the Euler equation provided by Theorem 1.1. We want to study the linearized operator ${{\mathcal {L}} }_\nu := {\mathrm d}{{\mathcal {F}}}_\nu (v_e)$ at the solution of the Euler equation $v_e$. where ${\mathcal {F}}_\nu (v)$ is defined in (3.1). The linearized operator has the form

3.3 $$\begin{aligned} \begin{aligned} {{\mathcal {L}}}_{\nu }&= {{\mathcal {L}}}_e - \nu \Delta , \\ {{\mathcal {L}}}_e&:= \omega \cdot \partial _\varphi + \big (\zeta + \varepsilon a(\varphi , x) \big )\cdot \nabla + \varepsilon {{\mathcal {R}}}(\varphi ) \end{aligned} \end{aligned}$$

where $a(\varphi ,x)$ is the function defined by

3.4 $$\begin{aligned} a(\varphi , x):= \nabla _\bot (- \Delta )^{- 1} v_e, \end{aligned}$$

with $\nabla _\bot$ as in (1.4) and ${{\mathcal {R}}}(\varphi )$ is a pseudo-differential operator of order $- 1$, given by

3.5 $$\begin{aligned} \begin{aligned}&{{\mathcal {R}}}(\varphi ) h:= \nabla v_e(\varphi ,x) \cdot \nabla _\bot (- \Delta )^{- 1}h . \end{aligned} \end{aligned}$$

Using that ${\textrm{div}} (\nabla _\bot h) = 0$ for any *h*, the operators $a \cdot \nabla$, ${{\mathcal {R}}}$ ${{\mathcal {L}}}_\nu$ and ${{\mathcal {L}}}_e$ leave invariant the subspace of zero average function, with

3.6 $$\begin{aligned} \begin{aligned}&[\Pi _0^\bot ,\, a \cdot \nabla ] = 0, \, [\Pi _0^\bot ,\, {{\mathcal {R}}}] = 0, \\&a \cdot \nabla \Pi _0 = \Pi _0 a \cdot \nabla = 0,\, {{\mathcal {R}}} \Pi _0 = \Pi _0 {{\mathcal {R}}} = 0 \\&[\Pi _0^\bot , {{\mathcal {L}}}_{\nu }] = 0 = [\Pi _0^\bot , {{\mathcal {L}}}_e], \quad \Pi _0 {{\mathcal {L}}}_{\nu } = {{\mathcal {L}}}_{\nu } \Pi _0 = 0, \quad \Pi _0 {{\mathcal {L}}}_{e} = {{\mathcal {L}}}_{e} \Pi _0 = 0 , \end{aligned} \end{aligned}$$

implying that

$$\begin{aligned} a \cdot \nabla = \Pi _0^\bot a \cdot \nabla \Pi _0^\bot , \quad {{\mathcal {R}}} = \Pi _0^\bot {{\mathcal {R}}} \Pi _0^\bot , \quad {{\mathcal {L}}}_{\nu } = \Pi _0^\bot {{\mathcal {L}}}_{\nu } \Pi _0^\bot , \quad {{\mathcal {L}}}_e = \Pi _0^\bot {{\mathcal {L}}}_e \Pi _0^\bot . \end{aligned}$$

We always work on the space of zero average functions and we shall preserve this invariance along the whole paper.

The goal of next two sections is to invert the whole linearized Navier–Stokes operator ${\mathcal {L}}_\nu$ obtained by linearizing the nonlinear functional ${\mathcal {F}}_\nu (v)$ in (3.1) at any quasi-periodic solution $v_e(\varphi ,x)|_{\varphi =\omega t}$ provided by Theorem 1.1 by requiring a smallness condition on $\varepsilon$ which is independent of the viscosity parameter $\nu > 0$. This is achieved in two steps. First, we fully reduce to a constant coefficient, diagonal operator the linearized Euler operator ${\mathcal {L}}_{e}$ in (3.3). This is done in Sect. 4, in the spirit of [4, 5], by combining a reduction to constant coefficients up to an arbitrarily regularizing remainder with a KAM reducibility scheme. We check step by step that this normal form procedure, when applied to the full operator ${\mathcal {L}}_{\nu }$ in (3.3), just perturbs the unbounded viscous term $-\nu \Delta$ by an unbounded pseudo differential operator of order two that “gain smallness”, namely it is of size $O(\nu \varepsilon )$, see (5.1)–(5.2). In Sect. 5, we use this normal form procedure in order to infer the invertibility of the operator ${\mathcal {L}}_{\nu }$ uniformly with respect to the viscosity parameter, namely by imposing a smallness condition on $\varepsilon$ that is independent of $\nu$. The inverse of the Navier–Stokes operator is bounded from $H^s_0$, *gains two space derivatives* and it has size $O(\nu ^{- 1})$ (see Proposition 5.4), whereas the inverse of the linearized Euler operator ${{{\mathcal {L}}}}_e$ loses $\tau$ derivatives, due to the small divisors (see Proposition 5.5). The invertibility of the linearized Euler operator ${{{\mathcal {L}}}}_e$ is used to construct the approximate solution in Sect. 6 and the invertibility of the linearized Navier Stokes operator ${{{\mathcal {L}}}}_\nu$ is used to implement the fixed point argument of Sect. 7.

## Normal Form Reduction of the Operator ${\mathcal {L}}_{\nu }$

In this section we reduce to a constant coefficients, diagonal operator the operator ${\mathcal {L}}_{\nu }$ in (3.3) up to an unbounded remainder of order two which is of size $O(\varepsilon \nu )$. First, we deal with the conjugation of the transport operator in Sect. 4.1, which is the highest order term in the operator ${\mathcal {L}}_{e}$. In Sect. 4.2, the lower order terms after the previous conjugation are regularized to constant coefficients up to a remainder of arbitrary smoothing matrix decay and up to an unbounded remainder of order two and size $O(\varepsilon \nu )$. Then, in Sects. 4.3–4.4 we perform the full KAM reducibility for the regularized version of the operator ${\mathcal {L}}_{e}$. In particular, in Sect. 4.3 the *n*-th iterative step of the reduction is performed and in Sect. 4.4 the convergence of the scheme is proved via Nash-Moser estimates to overcome the loss of derivatives coming from the small divisors. The linearized Navier Stokes operator is then reduced to a diagonal operator plus an unbounded operator of order two and size $O(\varepsilon \nu )$ in (5.1)–(5.2). This is the starting point for its inversion in Sect. 5.

From now on, the parameters $\gamma \in (0,1)$ and $\tau >0$, characterizing the set $\Lambda (\gamma ,\tau )$ in (2.15) of the Diophantine frequencies in a given measurable set $\Lambda$, are considered as fixed and $\tau$ is chosen in (8.2) and $\gamma$ at the end of Sect. 8, see (8.6). Therefore we omit to recall them each time. Moreover, from now on, we denote by $DC(\gamma , \tau )$, the set of Diophantine frequencies in $\Omega _\varepsilon$, where the set $\Omega _\varepsilon$ is provided in Theorem 1.1, namely $\Lambda (\gamma , \tau )$ with $\Lambda = \Omega _\varepsilon$. We repeat the definition for clarity of the reader:

4.1 $$\begin{aligned} DC(\gamma , \tau ):= \big \{ (\omega ,\zeta )\in \Omega _\varepsilon :\, |\omega \cdot \ell +\zeta \cdot j| \ge \frac{\gamma }{|(\ell ,j)|^{\tau }}, \ \forall \,(\ell ,j)\in {\mathbb {Z}}^{d+2}\setminus \{0\} \big \}.\nonumber \\ \end{aligned}$$

### Reduction of the Highest Order Term

First, we state the proposition that allows to reduce to constant coefficients the highest order operator

$$\begin{aligned} {{\mathcal {T}}}:= \omega \cdot \partial _\varphi + \big ( \zeta + \varepsilon a(\varphi , x) \big ) \cdot \nabla \end{aligned}$$

where we recall, by (3.4), that $\Pi _0 a = 0$ and ${\textrm{div}}(a) = 0$. The result has been proved in Proposition 4.1 in [4] (see also [23] for a more general result of this kind). We restate it with clear adaptation to our case and we refer to the former for the proof.

#### Proposition 4.1

(Straightening of the transport operator ${\mathcal {T}}$). There exist $\sigma := \sigma (\tau , d) > 0$ large enough such that for any $S > s_0 + \sigma$ there exists $\delta := \delta (S, \tau , d) \in (0, 1)$ small enough such that if (3.2) holds and

4.2 $$\begin{aligned} \varepsilon \gamma ^{- 1} \le \delta , \end{aligned}$$

are fulfilled, then the following holds. There exists an invertible diffeomorphism ${\mathbb {T}}^2 \rightarrow {\mathbb {T}}^2$, $x \mapsto x + \alpha (\varphi , x; \omega , \zeta )$ with inverse $y \mapsto y + \breve{\alpha }(\varphi , y; \omega , \zeta )$, defined for all $(\omega , \zeta ) \in DC(\gamma , \tau )$, with the set given in (4.1), satisfying, for any $s_0\le s \le S - \sigma$,

4.3 $$\begin{aligned} \Vert \alpha \Vert _s^{{\textrm{Lip}}(\gamma )}, \Vert \breve{\alpha }\Vert _{s}^{{\textrm{Lip}}(\gamma )} \lesssim _{s} \varepsilon \gamma ^{- 1} \end{aligned}$$

such that, by defining

4.4 $$\begin{aligned} {{\mathcal {A}}} h (\varphi , x):= h(\varphi , x + \alpha (\varphi , x)), \quad \text {with } \quad {{\mathcal {A}}}^{- 1} h(\varphi , y) = h(\varphi , y + \breve{\alpha }(\varphi , y)),\nonumber \\ \end{aligned}$$

one gets the conjugation

$$\begin{aligned} {{\mathcal {A}}}^{- 1} {{\mathcal {T}}} {{\mathcal {A}}} = \omega \cdot \partial _\varphi + \zeta \cdot \nabla \end{aligned}$$

Furthermore, $\alpha ,\breve{\alpha }$ are $\text {odd}(\varphi ,x)$ and the maps ${{\mathcal {A}}}, {{\mathcal {A}}}^{- 1}$ are reversibility preserving, satisfying the estimates

4.5 $$\begin{aligned} \begin{aligned}&\Vert {{\mathcal {A}}}^{\pm 1} h\Vert _s^{{\textrm{Lip}}(\gamma )} \lesssim _{s} \Vert h \Vert _s^{{\textrm{Lip}}(\gamma )}. \end{aligned} \end{aligned}$$

We remark that the assumptions of Proposition 4.1 are satisfied by the ansatz (3.2) and by the choice of the parameter $\gamma >0$ at the end of Sect. 8 in (8.6). In particular, the smallness condition (4.2) becomes $\varepsilon \gamma ^{-1}= \varepsilon ^{1-\frac{\mathtt a}{2}} \ll 1$, which is clearly satisfied since $\mathtt a \in (0,1)$ and for $\varepsilon$ sufficiently small.

In order to study the conjugation of the operator ${{\mathcal {L}}}_{\nu }: H^{s+2}_0 \rightarrow H^{s}_0$ in (3.3) under the transformation ${\mathcal {A}}$, we need the following auxiliary Lemma.

#### Lemma 4.2

Let $S > s_0 + \sigma + 2$ (where $\sigma$ is the constant appearing in Proposition 4.1). Then there exists $\delta := \delta (S, \tau , d) \in (0, 1)$ small enough such that if (3.2), (4.2) are fulfilled, the following hold:

(i) Let ${{\mathcal {A}}}_\bot := \Pi _0^\bot {{\mathcal {A}}} \Pi _0^\bot$. Then, for any $s_0\le s\le S - \sigma$, the operator ${{\mathcal {A}}}_\bot : H^s_0 \rightarrow H^s_0$ is invertible with bounded inverse given by ${{\mathcal {A}}}_\bot ^{- 1} = \Pi _0^\bot {{\mathcal {A}}}^{- 1} \Pi _0^\bot : H^s_0 \rightarrow H^s_0$;
(ii) Let $s_0\le s\le S - \sigma - 1$, $a (\cdot ; \lambda ) \in H^{s + 1}({\mathbb {T}}^{d + 2})$ and let ${{\mathcal {R}}}_a$ be the linear operator defined by
  $$\begin{aligned} {{\mathcal {R}}}_a: h(\varphi , x) \mapsto \nabla a(\varphi , x) \cdot \nabla ^\bot h(\varphi , x). \end{aligned}$$
  Then $${{\mathcal {A}}}_\bot ^{- 1} {{\mathcal {R}}}_a {{\mathcal {A}}}_\bot \in {{\mathcal {O}}}{{\mathcal {P}}}{{\mathcal {M}}}^1_s$$ and $$|{{\mathcal {A}}}_\bot ^{- 1} {{\mathcal {R}}}_a {{\mathcal {A}}}_\bot |_{1, s}^{{\textrm{Lip}}(\gamma )} \lesssim _s \Vert a \Vert _{s + 1}^{{\textrm{Lip}}(\gamma )}$$;
(iii) For any $s_0\le s\le S- \sigma - 2$, the operator ${{\mathcal {P}}}_\Delta := {{\mathcal {A}}}_\bot ^{-1} (- \Delta ) {{\mathcal {A}}}_\bot =- \Delta +{\mathcal {R}}_{\Delta }: H^{s+2}_0 \rightarrow H_{0}^{s}$ is in ${{\mathcal {O}}{{\mathcal {P}}}{{\mathcal {M}}}}^2_s$, with estimates
  4.6 $$\begin{aligned} |{\mathcal {R}}_{\Delta }|_{2,s}^{{{\textrm{Lip}}(\gamma )}} \lesssim _s \varepsilon \gamma ^{- 1}. \end{aligned}$$
(iv) For any $s_0\le s \le S- \sigma - 2$, the operator ${{\mathcal {P}}}_\Delta$ is invertible, with inverse of the form
  $$\begin{aligned} {{\mathcal {P}}}_\Delta ^{- 1} = {{\mathcal {A}}}_\bot ^{- 1} (- \Delta )^{- 1}{{\mathcal {A}}}_\bot \in {{\mathcal {O}}}{{\mathcal {P}}}{{\mathcal {M}}}_s^{- 2} \end{aligned}$$
  satisfying the estimates
  $$\begin{aligned} |{{\mathcal {P}}}_\Delta ^{- 1}|_{- 2, s}^{{\textrm{Lip}}(\gamma )} \lesssim _{s} 1. \end{aligned}$$

#### Proof

Proof of (i). For any $u \in H^s({\mathbb {T}}^{d + 2})$, we split $u = \Pi _0^\bot u + \Pi _0 u$, where $\Pi _0^\bot u \in H^s_0 = H^s_0({\mathbb {T}}^{d + 2})$ and $\Pi _0 u \in H^s_\varphi := H^s({\mathbb {T}}^d)$. Since ${{\mathcal {A}}}$ is an operator of the form (4.4), one has that ${{\mathcal {A}}} h = h$ if $h(\varphi )$ does not depend on *x*. This implies that ${{\mathcal {A}}} \Pi _0 = \Pi _0$. Similarly, one can show that ${{\mathcal {A}}}^{- 1} \Pi _0 = \Pi _0$ and therefore

4.7 $$\begin{aligned} \Pi _0^\bot {{\mathcal {A}}} \Pi _0 = 0 \quad \text {and} \quad \Pi _0^\bot {{\mathcal {A}}}^{- 1} \Pi _0 = 0 . \end{aligned}$$

We now show that the operator $\Pi _0^\bot {{\mathcal {A}}}^{- 1} \Pi _0^\bot : H^s_0 \rightarrow H^s_0$ is the inverse of the operator ${\mathcal {A}}_\perp :=\Pi _0^\bot {{\mathcal {A}}} \Pi _0^\bot : H^s_0 \rightarrow H^s_0$. By (4.7), one has

$$\begin{aligned} \begin{aligned} (\Pi _0^\bot {{\mathcal {A}}}^{- 1} \Pi _0^\bot )(\Pi _0^\bot {{\mathcal {A}}} \Pi _0^\bot )&= \Pi _0^\bot {{\mathcal {A}}}^{- 1} \big ( {\textrm{Id}} - \Pi _0 \big ) {{\mathcal {A}}} \Pi _0^\bot \\&= \Pi _0^\bot {{\mathcal {A}}}^{- 1} {{\mathcal {A}}} \Pi _0^\bot - \Pi _0^\bot {{\mathcal {A}}}^{- 1} \Pi _0 {{\mathcal {A}}} \Pi _0^\bot = \Pi _0^\bot \end{aligned} \end{aligned}$$

and similarly $(\Pi _0^\bot {{\mathcal {A}}} \Pi _0^\bot ) (\Pi _0^\bot {{\mathcal {A}}}^{- 1} \Pi _0^\bot )=\Pi _0^\perp$. The claimed statement then follows.

Proof of (ii). First, we note that, given a function $h(\varphi , x)$ and integrating by parts

$$\begin{aligned} \begin{aligned} \Pi _0[ {{\mathcal {R}}}_a h]&= \frac{1}{(2 \pi )^2} \int _{{\mathbb {T}}^2} \nabla a(\varphi , x) \cdot \nabla ^\bot h(\varphi , x)\, \textrm{d} x \\&= - \frac{1}{(2 \pi )^2} \int _{{\mathbb {T}}^2} a(\varphi , x) {\textrm{div}}\big ( \nabla ^\bot h(\varphi , x) \big )\, \textrm{d} x = 0 \end{aligned} \end{aligned}$$

since ${\textrm{div}}(\nabla ^\bot h) = 0$ for any function *h*. Moreover it is easy to see that ${{\mathcal {R}}}_a \Pi _0 = 0$. This implies that the linear operator ${{\mathcal {R}}}_a$ is invariant on the space of zero average functions. Then, using also item (i), one has that

4.8 $$\begin{aligned} \begin{aligned} {{\mathcal {A}}}_\bot ^{- 1} {{\mathcal {R}}}_a {{\mathcal {A}}}_\bot&= \Pi _0^\bot {{\mathcal {A}}}^{- 1} {{\mathcal {R}}}_a {{\mathcal {A}}} \Pi _0^\bot = \Pi _0^\bot ({{\mathcal {A}}}^{- 1} {{\mathcal {M}}}_{\nabla a} {{\mathcal {A}}} ) \cdot ({{\mathcal {A}}}^{- 1} \nabla ^\bot {{\mathcal {A}}}) \Pi _0^\bot , \end{aligned} \end{aligned}$$

where ${{\mathcal {M}}}_{\nabla a}$ denotes the multiplication operator by $\nabla a$. A direct calculation shows that the operator ${{\mathcal {A}}}^{- 1}{{\mathcal {M}}}_{\nabla a} {{\mathcal {A}}} = {{\mathcal {M}}}_g$, where the function $g (\varphi , y):= \nabla a (\varphi , y + \breve{\alpha }(\varphi , y)) = \{{{\mathcal {A}}}^{- 1} \nabla a\}(\varphi , y)$. The estimates (2.13), (4.5) imply that

4.9 $$\begin{aligned} |{{\mathcal {A}}}^{- 1} {{\mathcal {M}}}_{\nabla a} {{\mathcal {A}}}|_{0, s} = |{{\mathcal {M}}}_g|_{0, s} \lesssim _s \Vert a \Vert _{s + 1}. \end{aligned}$$

Moreover, one computes ${{\mathcal {A}}}^{- 1} \partial _{x_i} {{\mathcal {A}}} \,[h]= \partial _{y_i} h + {{\mathcal {A}}}^{- 1}[\partial _{x_i} \alpha ] \cdot \nabla h$, for $i=1,2$. Using that $|\partial _{x_1}|_{1, s}, |\partial _{x_2}|_{1, s} \le 1$ and by applying the estimates (2.13), (4.3), (4.5), (4.2), one obtains that

4.10 $$\begin{aligned} |{{\mathcal {A}}}^{- 1} \nabla ^\bot {{\mathcal {A}}}|_{1, s} \lesssim _s 1 + \Vert \alpha \Vert _{s + 1} \lesssim _s 1. \end{aligned}$$

Using the trivial fact that $|\Pi _0^\bot |_{0, s} \le 1$, the formula (4.8), the estimates (4.9), (4.10), together with the composition Lemma 2.6-(ii), imply the claimed bound.

Proof of (iii). Since $\Pi _0 \Delta = \Delta \Pi _0 = 0$, one computes

$$\begin{aligned} {{\mathcal {P}}}_\Delta = {{\mathcal {A}}}_\bot ^{- 1} (- \Delta ) {{\mathcal {A}}}_\bot = \Pi _0^\bot {{\mathcal {A}}}^{- 1} (- \Delta ) {{\mathcal {A}}} \Pi _0^\bot . \end{aligned}$$

and hence, a direct calculation shows that

4.11 $$\begin{aligned} \begin{aligned}&{{\mathcal {A}}}_\bot ^{- 1} (- \Delta ) {{\mathcal {A}}}_\bot = - \Delta + {{\mathcal {R}}}_\Delta , \\&{{\mathcal {R}}}_\Delta := \Pi _0^\bot \left( \sum _{i, j = 1}^2 {\mathcal {A}}^{-1} [a_{i j}]\, \partial _{y_i y_j} + \sum _{k = 1}^2 {\mathcal {A}}^{-1} [b_k ] \, \partial _{y_k} \right) \Pi _0^\bot , \end{aligned} \end{aligned}$$

where

$$\begin{aligned} \begin{aligned} a_{11}(\varphi ,x)&:=-\alpha _{x_1}(\varphi ,x)(2+\alpha _{x_1}(\varphi ,x)),\quad a_{22}(\varphi ,x):=-\alpha _{x_2}(\varphi ,x)(2+\alpha _{x_2}(\varphi ,x)), \\ a_{12}(\varphi ,x)&=a_{21}(\varphi ,x)=-\alpha _{x_1}(\varphi ,x)\alpha _{x_2}(\varphi ,x) -\tfrac{1}{2}(\alpha _{x_1}(\varphi ,x)+\alpha _{x_2}(\varphi ,x)), \\ b_{1}(\varphi ,x)&:=-\alpha _{x_1 x_1}(\varphi ,x)-\alpha _{x_1 x_2}(\varphi ,x), \quad b_{2}(\varphi ,x):=-\alpha _{x_1 x_2}(\varphi ,x)-\alpha _{x_2 x_2}(\varphi ,x). \end{aligned} \end{aligned}$$

By Lemma 2.3 and the estimate (4.3), we have, for any $s_0\le s \le S- \sigma - 2$

4.12 $$\begin{aligned} \Vert {\mathcal {A}}^{-1} [a_{i j}] \Vert _s^{{\textrm{Lip}}(\gamma )},\, \Vert {\mathcal {A}}^{-1}[b_k] \Vert _s^{{\textrm{Lip}}(\gamma )} \lesssim _s \Vert \alpha \Vert _{s + 2}^{{\textrm{Lip}}(\gamma )} + (\Vert \alpha \Vert _{s + 2}^{{\textrm{Lip}}(\gamma )})^2 \lesssim _s \varepsilon \gamma ^{- 1}\nonumber \\ \end{aligned}$$

By (4.11), Lemma 2.3, estimates (2.13), (2.20), (4.12), Lemma  2.6-(ii) together with the trivial fact that $|\partial _{x_j}|_{1, s}\,,\, |\partial _{x_i x_j}|_{2, s} \lesssim 1$, we conclude that ${\mathcal {R}}_{\Delta }$ satisfies the claimed bound.

Proof of (iv). We write

$$\begin{aligned} {{\mathcal {P}}}_\Delta = - \Delta + {{\mathcal {R}}}_\Delta = (- \Delta ) \big ( {\textrm{Id}}_0 + (- \Delta )^{- 1} {{\mathcal {R}}}_\Delta \big ) \end{aligned}$$

where ${\textrm{Id}}_0$ is the identity on the space of the $L^2$ zero average functions. By Lemma 2.6-(ii), estimates (2.21), (4.6), one obtains that $|(- \Delta )^{- 1} {{\mathcal {R}}}_\Delta |_{0, s}^{{\textrm{Lip}}(\gamma )} \lesssim _s \varepsilon \gamma ^{- 1}$. Hence, by the smallness condition in (4.2) and by Lemma 2.6-(iv), one gets that ${\textrm{Id}}_0 + (- \Delta )^{- 1} {{\mathcal {R}}}_\Delta : H^s_0 \rightarrow H^s_0$ is invertible, with $|\big ( {\textrm{Id}}_0 + (- \Delta )^{- 1} {{\mathcal {R}}}_\Delta \big )^{- 1}|_{0, s}^{{\textrm{Lip}}(\gamma )} \lesssim _s 1$. The claimed statement then follows since we have

$$\begin{aligned} {{\mathcal {P}}}_\Delta ^{- 1} = \big ( {\textrm{Id}}_0 + (- \Delta )^{- 1} {{\mathcal {R}}}_\Delta \big )^{- 1}(- \Delta )^{- 1} \end{aligned}$$

using again Lemma 2.6-(ii). $\square$

We conclude this section by conjugating the whole operator ${{\mathcal {L}}}$ defined in (3.3) by means of the map ${{\mathcal {A}}}$ constructed in Proposition 4.1.

#### Proposition 4.3

Let $S > s_0 + \sigma + 2$ (where $\sigma$ is the constant appearing in Proposition 4.1). Then there exists $\delta := \delta (S, \tau , d) \in (0, 1)$ small enough such that, if (3.2) and (4.2) are fulfilled, the following holds. For any $(\omega , \zeta ) \in DC(\gamma , \tau )$ defined in (4.1), one has

4.13 $$\begin{aligned} \begin{aligned} {{\mathcal {L}}}^{(1)}_\nu&:= {{\mathcal {A}}}_\bot ^{- 1} {{\mathcal {L}}} {{\mathcal {A}}}_\bot = {{\mathcal {L}}}^{(1)}_e - \nu \Delta + {{\mathcal {R}}}_\nu ^{(1)} , \\ {{\mathcal {L}}}^{(1)}_e&:= {{\mathcal {A}}}^{- 1}_\bot {{\mathcal {L}}}_e {{\mathcal {A}}}_\bot = \omega \cdot \partial _\varphi + \zeta \cdot \nabla +{{\mathcal {R}}}^{(1)} \end{aligned} \end{aligned}$$

where the map ${\mathcal {A}}_\perp$ is defined as in Proposition 4.1 and Lemma 4.2-(i), whereas, for any $s_0 \le s \le S - \sigma - 2$, the operators ${{\mathcal {R}}}^{(1)} \in {{\mathcal {O}}}{{\mathcal {P}}}{{\mathcal {M}}}^{- 1}_{s}$ and ${{\mathcal {R}}}^{(1)}_\nu \in {{\mathcal {O}}}{{\mathcal {P}}}{{\mathcal {M}}}^2_s$ satisfy the estimates

4.14 $$\begin{aligned} \begin{aligned}&|{{\mathcal {R}}}^{(1)}|_{- 1, s}^{{\textrm{Lip}}(\gamma )} \lesssim _{s} \varepsilon , \qquad |{{\mathcal {R}}}_\nu ^{(1)}|_{2, s}^{{\textrm{Lip}}(\gamma )} \lesssim _s \varepsilon \gamma ^{- 1} \,\nu . \end{aligned} \end{aligned}$$

Moreover, the operators ${{\mathcal {L}}}^{(1)}_e$ and ${{\mathcal {R}}}^{(1)}$ are real and reversible and ${{\mathcal {R}}}^{(1)}, {{\mathcal {R}}}_\nu ^{(1)}$ leave invariant the space of functions with zero average in *x*.

#### Proof

By recalling (3.3) and by Lemma 4.2-(i), one gets ${{\mathcal {L}}}^{(1)}_\nu = {{\mathcal {L}}}_e^{(1)} - \nu {{\mathcal {A}}}_\bot ^{- 1} \Delta {{\mathcal {A}}}_\bot$, where, by (3.6),

$$\begin{aligned} \begin{aligned} {{\mathcal {L}}}_e^{(1)}&:= {{\mathcal {A}}}_\bot ^{- 1} {{\mathcal {L}}}_e {{\mathcal {A}}}_\bot = {{\mathcal {A}}}_\bot ^{- 1} \big ( \omega \cdot \partial _\varphi + (\zeta + \varepsilon a(\varphi , x)) \cdot \nabla \big ) {{\mathcal {A}}}_\bot + \varepsilon {{\mathcal {A}}}_\bot ^{- 1} {{\mathcal {R}}} {{\mathcal {A}}}_\bot \\&:= \Pi _0^\bot {{\mathcal {A}}}^{- 1}\Pi _0^\bot \big ( \omega \cdot \partial _\varphi + (\zeta + \varepsilon a(\varphi , x)) \cdot \nabla \big ) \Pi _0^\bot {{\mathcal {A}}} \Pi _0^\bot + \varepsilon {{\mathcal {A}}}_\bot ^{- 1} {{\mathcal {R}}} {{\mathcal {A}}}_\bot \\&= \Pi _0^\bot {{\mathcal {A}}}^{- 1} \big ( \omega \cdot \partial _\varphi + (\zeta + \varepsilon a(\varphi , x)) \cdot \nabla \big ) {{\mathcal {A}}} \Pi _0^\bot + \varepsilon {{\mathcal {A}}}_\bot ^{- 1} {{\mathcal {R}}} {{\mathcal {A}}}_\bot . \end{aligned} \end{aligned}$$

By Proposition 4.1, using the formula (3.3), one has that

$$\begin{aligned} {{\mathcal {L}}}^{(1)}_e = \omega \cdot \partial _\varphi + \zeta \cdot \nabla + {{\mathcal {R}}}^{(1)}, \quad {{\mathcal {R}}}^{(1)}:= \varepsilon {{\mathcal {A}}}_\bot ^{- 1} {{\mathcal {R}}} {{\mathcal {A}}}_\bot . \end{aligned}$$

By (3.5), one writes, according to the notation of Lemma 4.2-(ii),

$$\begin{aligned} {{\mathcal {R}}} = {{\mathcal {R}}}_{v_e} \circ (- \Delta )^{- 1}, \quad {{\mathcal {R}}}_{v_e}: h(\varphi , x) \mapsto \nabla v_e \cdot \nabla ^\bot h(\varphi , x). \end{aligned}$$

Therefore,

$$\begin{aligned} \begin{aligned} {{\mathcal {R}}}^{(1)}&= \varepsilon {{\mathcal {A}}}_\bot ^{- 1} {{\mathcal {R}}} {{\mathcal {A}}}_\bot = \varepsilon \big ( {{\mathcal {A}}}_\bot ^{- 1} {{\mathcal {R}}}_{v_e} {{\mathcal {A}}}_\bot \big ) \circ \big ({{\mathcal {A}}}_\bot ^{- 1} (- \Delta )^{- 1} {{\mathcal {A}}}_\bot \big ). \end{aligned} \end{aligned}$$

Then, by Lemma 4.2, (ii), (iv) Lemma 2.6-(ii), the ansatz (3.2) and $\varepsilon \gamma ^{- 1} \le \delta < 1$, one gets the claimed bound (4.14) for ${{\mathcal {R}}}^{(1)}$. By Lemma 4.2-(iii), one has $- \nu {{\mathcal {A}}}_\bot ^{- 1} \Delta {{\mathcal {A}}}_\bot = - \nu \Delta + {{\mathcal {R}}}_\nu ^{(1)}$, with ${{\mathcal {R}}}_\nu ^{(1)}:= \nu \Pi _0^{\bot } \big ( \Delta - {{\mathcal {A}}}^{- 1} \Delta {{\mathcal {A}}} \big ) \Pi _0^\bot$ satisfying the estimate $|{{\mathcal {R}}}_\nu ^{(1)}|_s^{{\textrm{Lip}}(\gamma )} \lesssim _s \varepsilon \gamma ^{- 1} \nu$, which is the second estimate in (4.14). Moreover, since ${{\mathcal {A}}}, {{\mathcal {A}}}^{- 1}$ are real and reversibility preserving and ${{\mathcal {R}}}$ is real and reversible, then ${{\mathcal {L}}}_e^{(1)}, {{\mathcal {R}}}^{(1)}$ are real and reversible. This concludes the proof. $\square$

### Reduction to Constant Coefficients of the Lower Order Terms

In this section we diagonalize the operator ${{\mathcal {L}}}^{(1)}_\nu$ in (4.13) up to a remainder of size $O(\varepsilon )$ and arbitrarily smoothing matrix decay and up to an unbounded operator of order 2 of size $O(\varepsilon \gamma ^{- 1} \nu )$. More precisely, we prove the following Proposition.

#### Proposition 4.4

Let $M\in {\mathbb {N}}$ be fixed. There exists $\sigma _{M - 1}:= \sigma _{M - 1}( \tau , d) > \sigma + 2$ large enough (where $\sigma$ is the constant appearing in Proposition 4.1) such that for any $S > s_0 + \sigma _{M - 1}$ there exists $\delta := \delta (S, M, \tau , d) \in (0, 1)$ small enough such that, if (3.2), (4.2) are fulfilled, the following holds. For any $(\omega , \zeta ) \in DC(\gamma , \tau )$ defined in (4.1), there exists a real and reversibility preserving, invertible map ${{\mathcal {B}}}$ satisfying, for any $s_0\le s\le S-\sigma _{M - 1}$,

4.15 $$\begin{aligned} {{\mathcal {B}}}^{\pm 1}: H^s_0 \rightarrow H^s_0, \quad |{{\mathcal {B}}}^{\pm 1}- {\textrm{Id}} |_{0, s}^{{\textrm{Lip}}(\gamma )} \lesssim _{s,M}\varepsilon \gamma ^{- 1} \end{aligned}$$

such that

4.16 $$\begin{aligned} \begin{aligned} {{\mathcal {L}}}^{(2)}_\nu&:= {{\mathcal {B}}}^{- 1}{{\mathcal {L}}}^{(1)}_\nu {{\mathcal {B}}} = {{\mathcal {L}}}_e^{(2)} - \nu \Delta + {{\mathcal {R}}}_\nu ^{(2)},\\ {{\mathcal {L}}}_e^{(2)}&:= {{\mathcal {B}}}^{- 1}{{\mathcal {L}}}^{(1)}_e {{\mathcal {B}}} = \omega \cdot \partial _\varphi + \zeta \cdot \nabla + {{\mathcal {Q}}} + {{\mathcal {R}}}^{(2)} \end{aligned} \end{aligned}$$

where ${{\mathcal {Q}}} \in {{\mathcal {O}}}{{\mathcal {P}}}{{\mathcal {M}}}^{- 1}_{s}$ is a diagonal operator, ${{\mathcal {R}}}^{(2)}$ belongs to ${{\mathcal {O}}}{{\mathcal {P}}}{{\mathcal {M}}}^{- M}_{s}$ and ${{\mathcal {R}}}^{(2)}_\nu \in {{\mathcal {O}}}{{\mathcal {P}}}{{\mathcal {M}}}^2_{s}$. Moreover, for any $s_0\le s \le S-\sigma _{M - 1}$,

4.17 $$\begin{aligned} \begin{aligned}&|{{\mathcal {Q}}}|_{- 1, s}^{{\textrm{Lip}}(\gamma )} \,, \, |{{\mathcal {R}}}^{(2)}|_{- M, s}^{{\textrm{Lip}}(\gamma )} \lesssim _{s, M} \varepsilon , \quad |{{\mathcal {R}}}^{(2)}_\nu |_{2, s}^{{\textrm{Lip}}(\gamma )} \lesssim _{s,M} \varepsilon \gamma ^{- 1} \,\nu . \end{aligned} \end{aligned}$$

The operators ${{\mathcal {Q}}}$, ${{\mathcal {R}}}^{(2)}$ are real, reversible and ${{\mathcal {Q}}}$, ${{\mathcal {R}}}^{(2)}, {{\mathcal {R}}}^{(2)}_\nu$ leave invariant the space of functions with zero average in *x*.

Proposition 4.4 follows by the following iterative lemma on the operator obtained by neglecting the viscosity term $- \nu \Delta + {{\mathcal {R}}}^{(2)}_\nu$ in (4.13), namely, in the next proposition, we only consider

4.18 $$\begin{aligned} {{\mathcal {L}}}^{(1)}_0 \equiv {{\mathcal {L}}}^{(1)}_e:= \omega \cdot \partial _\varphi + \zeta \cdot \nabla + {{\mathcal {R}}}^{(1)}. \end{aligned}$$

#### Lemma 4.5

Let $M \in {\mathbb {N}}$. There exist $0< \sigma _0< \sigma _1< \ldots < \sigma _{M - 1}$ large enough such that for any $S > s_0 + \sigma _{M - 1}$ there exists $\delta := \delta (S, \tau , d) \in (0, 1)$ small enough such that, if (3.2), (4.2) are fulfilled, the following holds. For any $n = 0, \ldots , M -1$ and any $(\omega , \zeta ) \in DC(\gamma , \tau )$, there exists a real, reversibility preserving, invertible map ${{\mathcal {T}}}_n$ satisfying, for any $s_0 \le s \le S-\sigma _{n}$

4.19 $$\begin{aligned} {{\mathcal {T}}}_n^{\pm 1}: H^s_0 \rightarrow H^s_0, \quad |{{\mathcal {T}}}_n^{\pm 1} - {\textrm{Id}}|_{- n,s}^{{\textrm{Lip}}(\gamma )} \lesssim _{s,n} \varepsilon \gamma ^{- 1} \end{aligned}$$

and, for any $n = 1, \ldots , M - 1$ and for any $(\omega , \zeta ) \in DC(\gamma , \tau )$,

4.20 $$\begin{aligned} {{\mathcal {L}}}^{(1)}_n = {{\mathcal {T}}}_n^{- 1}{{\mathcal {L}}}_{n - 1}^{(1)} {{\mathcal {T}}}_n , \end{aligned}$$

where ${{\mathcal {L}}}^{(1)}_n$ has the form

4.21 $$\begin{aligned} {{\mathcal {L}}}^{(1)}_n:= \omega \cdot \partial _\varphi + \zeta \cdot \nabla + {{\mathcal {Z}}}_n + {{\mathcal {R}}}_n^{(1)} \end{aligned}$$

where ${{\mathcal {Z}}}_n \in {{\mathcal {O}}}{{\mathcal {P}}}{{\mathcal {M}}}^{- 1}_{s}$ is diagonal, ${{\mathcal {R}}}_n^{(1)} \in {{\mathcal {O}}}{{\mathcal {P}}}{{\mathcal {M}}}^{- (n + 1) }_{s}$ and they satisfy

4.22 $$\begin{aligned} \begin{aligned}&|{{\mathcal {Z}}}_n|_{- 1, s}^{{\textrm{Lip}}(\gamma )} \lesssim _{s,n} \varepsilon \quad |{{\mathcal {R}}}_n^{(1)}|_{- (n + 1), s}^{{\textrm{Lip}}(\gamma )} \lesssim _{s,n} \varepsilon \quad \ \forall s_0 \le s \le S-\sigma _{n}. \end{aligned} \end{aligned}$$

The operators ${{\mathcal {L}}}_n^{(1)}, {{\mathcal {Z}}}_n$ and ${{\mathcal {R}}}_n^{(1)}$ are real, reversible and leave invariant the space of zero average functions in *x*.

#### Proof

We prove the lemma arguing by induction. For $n = 0$ the desired properties follow by Proposition 4.3, by defining ${{\mathcal {L}}}^{(1)}_0$ as in (4.18), ${{\mathcal {Z}}}_0 = 0$, ${{\mathcal {R}}}^{(1)}_0:= {{\mathcal {R}}}^{(1)}$ and $\sigma _0 = \sigma$ given in Proposition 4.3.

We assume that the claimed statement holds for some $n \in \{0, \ldots , M - 2\}$ and we prove it at the step $n+1$. Let us consider a transformation ${{\mathcal {T}}}_{n+1} = {\textrm{Id}} +{{\mathcal {K}}}_{n+1}$ where ${{\mathcal {K}}}_{n+1}$ is an operator of order $- (n+1)$ which has to be determined. One computes

4.23 $$\begin{aligned} \begin{aligned} {{\mathcal {L}}}_n^{(1)} {{\mathcal {T}}}_{n + 1}&= {{\mathcal {T}}}_{n + 1} \big ( \omega \cdot \partial _\varphi + \zeta \cdot \nabla \big ) + {{\mathcal {Z}}}_n + (\omega \cdot \partial _\varphi {{\mathcal {K}}}_{n + 1}) + [\zeta \cdot \nabla , {{\mathcal {K}}}_{n + 1}] + {{\mathcal {R}}}_n^{(1)}\\&\quad + {{\mathcal {Z}}}_n {{\mathcal {K}}}_{n + 1} + {{\mathcal {R}}}_n^{(1)}{{\mathcal {K}}}_{n + 1}. \end{aligned}\nonumber \\ \end{aligned}$$

By the induction hypothesis ${{\mathcal {R}}}_n^{(1)} \in {{\mathcal {O}}}{{\mathcal {P}}}{{\mathcal {M}}}_{s}^{- (n + 1)}$, with $|{{\mathcal {R}}}_n^{(1)} |_{- (n + 1), s} \lesssim _{s,n+1} \varepsilon$ for any $s_0\le s \le S- \sigma _{n}$. Therefore, by Lemma 2.7, there exists ${{\mathcal {K}}}_{n + 1} \in {{\mathcal {O}}}{{\mathcal {P}}}{{\mathcal {M}}}_{s}^{- (n + 1)}$ solving the homological equation

4.24 $$\begin{aligned} \begin{aligned}&\omega \cdot \partial _\varphi \,{{{\mathcal {K}}}}_{n + 1} + [\zeta \cdot \nabla , {{{\mathcal {K}}}}_{n + 1}] + {{\mathcal {R}}}_n^{(1)} = {{\mathcal {D}}}_{{{\mathcal {R}}}_n^{(1)}}, \end{aligned} \end{aligned}$$

with ${{\mathcal {D}}}_{{{\mathcal {R}}}_n^{(1)}}$ as in Definition 2.4, satisfying, for any $s_0\le s \le S-\sigma _{n + 1}$ (for an arbitrary $\sigma _{n + 1} > \sigma _n + 2 \tau + 1$),

4.25 $$\begin{aligned} |{{\mathcal {K}}}_{n + 1}|_{- (n + 1), s}^{{\textrm{Lip}}(\gamma )} \lesssim _{s,n+1} \gamma ^{- 1}|{{\mathcal {R}}}_n^{(1)} |^{{\textrm{Lip}}(\gamma )}_{- (n + 1), s + 2 \tau + 1} \lesssim _{s,n+1} \varepsilon \gamma ^{- 1}. \end{aligned}$$

By Lemma 2.6-(iv), we obtain that ${{\mathcal {T}}}_{n + 1} = {\textrm{Id}} + {{\mathcal {K}}}_{n + 1}$ is invertible with inverse ${{\mathcal {T}}}_{n + 1}^{- 1}$ satisfying the estimate, for $s_0\le s\le S-\sigma _{n + 1}$,

4.26 $$\begin{aligned} |{{\mathcal {T}}}_{n + 1}^{- 1} - {\textrm{Id}}|_{- (n + 1), s}^{{\textrm{Lip}}(\gamma )} \lesssim _{s,n+1} \varepsilon \gamma ^{- 1}. \end{aligned}$$

Hence the estimate (4.19) at the step $n + 1$ holds. By (4.23), (4.24) we get, for any $(\omega , \zeta ) \in DC(\gamma , \tau )$, the conjugation (4.20) at the step $n + 1$ where ${{\mathcal {L}}}^{(1)}_{n + 1}$ has the form (4.21), with

$$\begin{aligned} \begin{aligned} {{\mathcal {Z}}}_{n + 1}&:= {{\mathcal {Z}}}_n + {{\mathcal {D}}}_{{{\mathcal {R}}}_n^{(1)}}, \\ {{\mathcal {R}}}_{n + 1}^{(1)}&:= ({{\mathcal {T}}}_{n + 1}^{- 1} - {\textrm{Id}}){{\mathcal {Z}}}_{n + 1} + {{\mathcal {T}}}_{n + 1}^{- 1} \big ( {{\mathcal {Z}}}_n{{{\mathcal {K}}}}_{n + 1} + {{\mathcal {R}}}_n^{(1)}{{{\mathcal {K}}}}_{n + 1} \big ) . \end{aligned} \end{aligned}$$

By Lemma 2.6-(*v*), since ${{\mathcal {R}}}_n^{(1)}$ and ${{\mathcal {Z}}}_n$ satisfy (4.22), we deduce that ${{\mathcal {Z}}}_{n + 1} \in {{\mathcal {O}}}{{\mathcal {P}}}{{\mathcal {M}}}_{s}^{- 1}$, with estimates $|{{\mathcal {Z}}}_{n + 1}|_{- 1,s}^{{\textrm{Lip}}(\gamma )} \lesssim _{s,n+1} \varepsilon$. Moreover, by (4.25), (4.26), Lemma 2.6-(ii) and the condition in (3.2), we obtain that ${{\mathcal {R}}}_{n + 1}^{(1)} \in \mathcal {OPM}_{s}^{- (n + 2)}$, with $|{{\mathcal {R}}}_{n + 1}^{(1)}|_{- (n + 2), s}^{{\textrm{Lip}}(\gamma )} \lesssim _{s,n+1} \varepsilon$ for any $s_0\le s\le S-\sigma _{n + 1}$, by fixing $\sigma _{n+1}:= \sigma _{n} + 2 \tau + 1 + n + 1$. This concludes the induction argument and therefore the claimed statement is proved. $\square$

#### Proof of Proposition 4.4

By (4.13), (4.18), Lemma 4.5 and by defining

$$\begin{aligned} \begin{aligned} {{\mathcal {B}}}&:= {{\mathcal {T}}}_1 \circ \ldots \circ {{\mathcal {T}}}_{M - 1} , \quad {{\mathcal {Q}}}:= {{\mathcal {Z}}}_{M - 1}, \quad {{\mathcal {R}}}^{(2)}:= {{\mathcal {R}}}_{M - 1}^{(1)}, \end{aligned} \end{aligned}$$

one obtains that

$$\begin{aligned} {{\mathcal {L}}}^{(2)}_e:= {{\mathcal {B}}}^{- 1} {{\mathcal {L}}}^{(1)}_e {{\mathcal {B}}} = \omega \cdot \partial _\varphi + \zeta \cdot \nabla + {{\mathcal {Q}}} + {{\mathcal {R}}}^{(2)} \end{aligned}$$

To deduce the claimed properties of ${{\mathcal {Q}}}$ and ${{\mathcal {R}}}^{(2)}$, it suffices to apply Lemma 4.5 for $n = M - 1$. The estimate (4.15) then follows by the estimate (4.19) on ${{\mathcal {T}}}_n$, $n = 1, \ldots , M - 1$, using the composition property stated in Lemma 2.6-(ii). We now study the conjugation ${{\mathcal {B}}}^{- 1}( - \nu \Delta + {{\mathcal {R}}}_\nu ^{(1)}){{\mathcal {B}}}$. One has

$$\begin{aligned} \begin{aligned} {{\mathcal {B}}}^{- 1} \big ( - \nu \Delta + {{\mathcal {R}}}_\nu ^{(1)} \big ){{\mathcal {B}}}&= - \nu {{\mathcal {B}}}^{- 1} \Delta {{\mathcal {B}}} + {{\mathcal {B}}}^{- 1} {{\mathcal {R}}}_\nu ^{(1)} {{\mathcal {B}} } = - \nu \Delta + {{\mathcal {R}}}_\nu ^{(2)}, \end{aligned} \end{aligned}$$

where

$$\begin{aligned} {{\mathcal {R}}}_\nu ^{(2)}:= - \nu \big ( \Delta ({{\mathcal {B}}} - {\textrm{Id}}) + ({{\mathcal {B}}}^{- 1} - {\textrm{Id}}) \Delta {{\mathcal {B}}} \big ) + {{\mathcal {B}}}^{- 1} {{\mathcal {R}}}_\nu ^{(1)} {{\mathcal {B}}}. \end{aligned}$$

By applying again Lemma 2.6-(ii) and the estimates (4.14), (4.15) (using also that $|\Delta |_{2, s} \le 1$ for any *s*), one gets that ${{\mathcal {R}}}_\nu ^{(2)}$ satisfies (4.17) for any $s_0\le s\le S-\sigma _{M - 1}$. The proof of the claimed statement is then concluded. $\square$

### KAM Reducibility

In this section we perform the KAM reducibility scheme for the operator obtained by neglecting the small viscosity term $- \nu \Delta + {{\mathcal {R}}}_{ \nu }^{(2)}$ from the operator ${{\mathcal {L}}}^{(2)}_\nu$ in (4.16), namely we only consider the operator ${{\mathcal {L}}}^{(2)}_e$. More precisely we consider the operator

4.27 $$\begin{aligned} \begin{aligned} {{\mathcal {L}}}_0&:= {{\mathcal {L}}}^{(2)}_e = \omega \cdot \partial _\varphi + {{\mathcal {D}}}_0 + {{\mathcal {R}}}_0, \\ {{\mathcal {D}}}_0&:= \zeta \cdot \nabla + {{\mathcal {Q}}}, \quad {{\mathcal {R}}}_0:= {{\mathcal {R}}}^{(2)}. \end{aligned} \end{aligned}$$

where the diagonal operator ${\mathcal {Q}}$ and the smoothing operator ${\mathcal {R}}^{(2)}$ are as in Proposition 4.4. Given $\tau , N_0 > 0$, we fix the constants

4.28 $$\begin{aligned} \begin{aligned}&M:= [4 \tau ] + 1, \quad \alpha := (1+\chi ^{-1})\tau _1+1 , \quad \beta := \alpha + 1 , \\&\tau _1:= 4\tau +2+M, \quad \Sigma (\beta ):= \sigma _{M - 1} +\beta , \\&N_{- 1}:= 1, \quad N_n:= N_0^{\chi ^n}, \quad n \ge 0, \quad \chi := 3/2 \end{aligned} \end{aligned}$$

where $[4\tau ]$ is the integer part of $4\tau$ and *M*, $\sigma _{M - 1}$ are introduced in Proposition 4.4.

By Proposition 4.4, replacing *s* by $s + \beta$ in (4.17) and having ${{\mathcal {Q}}} = {\textrm{diag}}_{j \in {\mathbb {Z}}^2 {\setminus } \{ 0 \}} q_0(j)$ diagonal, one gets the initialization conditions for the KAM reducibility, for any $s_0\le s\le S-\Sigma (\beta )$,

4.29 $$\begin{aligned} \sup _{j \in {\mathbb {Z}}^2 \setminus \{ 0 \}} |j| |q_0(j)|^{{\textrm{Lip}}(\gamma )}, |{{\mathcal {R}}}_0|_{- M, s + \beta }^{{\textrm{Lip}}(\gamma )} \le C(S) \varepsilon . \end{aligned}$$

#### Proposition 4.6

(Reducibility). Let $S > s_0 + \Sigma (\beta )$. There exist $N_0:= N_0(S, \tau , d) > 0$ large enough and $\delta := \delta (S, \tau , d) \in (0, 1)$ small enough such that, if (3.2) holds and

4.30 $$\begin{aligned} N_0^{\tau _1}\varepsilon \gamma ^{-1} \le \delta , \end{aligned}$$

then the following statements hold for any integer $n \ge 0$.

$\mathbf{(S1)}_n$ There exists a real and reversible operator

4.31 $$\begin{aligned} \begin{aligned} {{\mathcal {L}}}_n&:= \omega \cdot \partial _\varphi + {{\mathcal {D}}}_n + {{\mathcal {R}}}_n: H^{s + 1}_0 \rightarrow H^s_0, \\ {{\mathcal {D}}}_n&:= \zeta \cdot \nabla + {{\mathcal {Q}}}_n = {\textrm{diag}}_{j \in {\mathbb {Z}}^2 \setminus \{ 0 \}} \mu _n(j) , \\ {{\mathcal {Q}}}_n&= {\textrm{diag}}_{j \in {\mathbb {Z}}^2 \setminus \{ 0 \}} q_n(j), \quad \mu _n(j):= {{\textrm{i}}}\, \zeta \cdot j + q_n(j), \end{aligned} \end{aligned}$$

defined for any $\lambda \in \Lambda _n^\gamma$, where we define $\Lambda _0^\gamma := DC(\gamma , \tau )$ for $n=0$ and, for $n \ge 1$,

4.32 $$\begin{aligned} \begin{aligned} \Lambda _n^\gamma&:= \Big \{ \lambda = (\omega , \zeta ) \in \Lambda _{n - 1}^\gamma : |{{\textrm{i}}}\,\omega \cdot \ell + \mu _{n - 1}(j) - \mu _{n - 1}(j') | \ge \frac{\gamma }{\langle \ell \rangle ^\tau |j|^\tau |j'|^\tau }, \\&\quad \forall \,\ell \in {\mathbb {Z}}^d, \ j,j' \in {\mathbb {Z}}^2 \setminus \{ 0 \}, \ \ (\ell , j, j') \ne (0, j, j), \ \ |\ell |, |j-j'| \le N_{n-1} \Big \}. \end{aligned}\nonumber \\ \end{aligned}$$

For any $j \in {\mathbb {Z}}^2 \setminus \{ 0 \}$, the eigenvalues $\mu _{n}(j)= \mu _{n}(j;\lambda )$ are purely imaginary and satisfy the conditions

4.33 $$\begin{aligned} \begin{aligned} \mu _n(j)&= - \mu _n(- j) = \overline{\mu _n(- j)}, \ \ \text {or equivalently} \\ q_n(j)&= - q_n(- j)= \overline{q_n(- j)}, \end{aligned} \end{aligned}$$

and the estimates

4.34 $$\begin{aligned}&|q_n(j)|^{{\textrm{Lip}}(\gamma )} \lesssim \varepsilon |j|^{- 1}\,, \quad | q_n(j)- q_0(j) |^{{\textrm{Lip}}(\gamma )} \lesssim \varepsilon |j|^{- M}, \quad \forall j \in {\mathbb {Z}}^2 \setminus \{ 0 \}\,, \end{aligned}$$

4.35 $$\begin{aligned}&| q_n(j) - q_{n - 1}(j)|^{{\textrm{Lip}}(\gamma )} \lesssim \varepsilon N_{n - 2}^{- \alpha } |j|^{- M} \quad \ \text {when } \quad n\ge 1 \,. \end{aligned}$$

The operator ${{\mathcal {R}}}_n$ is real and reversible, satisfying, for any $s_0\le s\le S-\Sigma (\beta )$,

4.36 $$\begin{aligned} \begin{aligned}&|{{\mathcal {R}}}_n |_{- M, s}^{{\textrm{Lip}}(\gamma )} \le C_*(s,\beta ) \varepsilon N_{n - 1}^{- \alpha }, \quad |{{\mathcal {R}}}_n |_{-M, s + \beta }^{{\textrm{Lip}}(\gamma )} \le C_*(s,\beta ) \varepsilon N_{n - 1} \end{aligned} \end{aligned}$$

for some constant $C_* (s) = C_*(s, \tau ) > 0$.

When $n \ge 1$, there exists an invertible, real and reversibility preserving map $\Phi _{n -1} = {\textrm{Id}} + \Psi _{n - 1}$, such that, for any $\lambda = (\omega , \zeta ) \in \Lambda _n^\gamma$,

4.37 $$\begin{aligned} {{\mathcal {L}}}_n = \Phi _{n - 1}^{- 1} {{\mathcal {L}}}_{n - 1} \Phi _{n - 1}. \end{aligned}$$

Moreover, for any $s_0\le s\le S-\Sigma (\beta )$, the map $\Psi _{n - 1}: H^s_0 \rightarrow H^s_0$ satisfies

4.38 $$\begin{aligned} \begin{aligned} |\Psi _{n - 1}|_{0, s}^{{\textrm{Lip}}(\gamma )}&\le C(s,\beta ) \varepsilon \gamma ^{- 1} N_{n - 1}^{4 \tau + 2} N_{n - 2}^{- \alpha }, \\ |\Psi _{n - 1} |_{0, s + \beta }^{{\textrm{Lip}}(\gamma )}&\le C(s,\beta ) \varepsilon \gamma ^{- 1} N_{n - 1}^{4 \tau + 2} N_{n - 2}, \end{aligned} \end{aligned}$$

for some constant $C(s,\beta )>0$.

$\mathbf{(S2)}_n$ For all $j \in {\mathbb {Z}}^2 \setminus \{ 0 \}$, there exist a Lipschitz extension of the eigenvalues $\mu _n(j;\,\cdot \,):\Lambda _n^\gamma \rightarrow {{\textrm{i}}}\, {\mathbb {R}}$ to the set $DC(\gamma , \tau )$, denoted by ${{\widetilde{\mu }}}_n(j;\,\cdot \,): DC(\gamma , \tau ) \rightarrow {{\textrm{i}}}\,{\mathbb {R}}$, satisfying, for $n \ge 1$,

4.39 $$\begin{aligned} |{{\widetilde{\mu }}}_n(j) - {{\widetilde{\mu }}}_{n - 1}(j) |^{{\textrm{Lip}}(\gamma )} \lesssim |j|^{- M} |{{\mathcal {R}}}_{n - 1}|_{-M,s_0}^{{\textrm{Lip}}(\gamma )} \lesssim |j|^{- M} \varepsilon N_{n - 2}^{- \alpha } . \end{aligned}$$

#### Proof

Proof of $\mathbf{(S1)}_0,\mathbf{(S2)}_0$. The claimed properties follow directly from Proposition 4.4, recalling (4.27), (4.29) and the definition of $\Lambda _0^\gamma := DC(\gamma , \tau )$.

Proof of $\mathbf{(S1)}_{n+1}$. By induction, we assume the claimed properties $\mathbf{(S1)}_n$, $\mathbf{(S2)}_n$ hold for some $n \ge 0$ and we prove them at the step $n + 1$. Let $\Phi _n = {\textrm{Id}} + \Psi _n$ where $\Psi _n$ is an operator to be determined. We compute

4.40 $$\begin{aligned} \begin{aligned} {{\mathcal {L}}}_n \Phi _n&= \Phi _n ( \omega \cdot \partial _\varphi + {{\mathcal {D}}}_n ) \\&\quad + \omega \cdot \partial _\varphi \Psi _n + [{{\mathcal {D}}}_n, \Psi _n] + \Pi _{N_n} {{\mathcal {R}}}_n \\&\quad + \Pi _{N_n}^\bot {{\mathcal {R}}}_n + {{\mathcal {R}}}_n \Psi _n \end{aligned} \end{aligned}$$

where ${{\mathcal {D}}}_n:= \zeta \cdot \nabla + {{\mathcal {Q}}}_n$ and the projectors $\Pi _N$, $\Pi _N^\bot$ are defined in (2.19). Our purpose is to find a map $\Psi _n$ solving the *homological equation*

4.41 $$\begin{aligned} \omega \cdot \partial _\varphi \Psi _n + [{{\mathcal {D}}}_n, \Psi _n] + \Pi _{N_n} {{\mathcal {R}}}_n ={{\mathcal {D}}}_{{{\mathcal {R}}}_n} \end{aligned}$$

where ${{\mathcal {D}}}_{{{\mathcal {R}}}_n}$ is the diagonal operator as per Definition 2.4. By (2.9) and (4.31), the homological Eq. (4.41) is equivalent to

4.42 $$\begin{aligned} \big ( {{\textrm{i}}}\,\omega \cdot \ell + \mu _{n}(j) - \mu _{n}(j') \big ) {{\widehat{\Psi }}}_{n}(\ell )_j^{j'} + \widehat{{\mathcal {R}}}_{n}(\ell )_j^{j'} = \widehat{{{\mathcal {D}}}_{{{\mathcal {R}}}_{n}}}(\ell )_j^{j'} \end{aligned}$$

for $\ell \in {\mathbb {Z}}^d$, $j, j' \in {\mathbb {Z}}^2 {\setminus } \{ 0 \}$. Therefore, we define the linear operator $\Psi _{n}$ by

4.43 $$\begin{aligned} {{\widehat{\Psi }}}_{n} (\ell )_j^{j'}:= {\left\{ \begin{array}{ll} - \dfrac{\widehat{{\mathcal {R}}}_{n}(\ell )_j^{j'} }{ {{\textrm{i}}}\, \omega \cdot \ell + \mu _{n}(j) - \mu _{n}(j') }, \quad \forall (\ell , j, j') \ne (0, j, j), &{}\quad |\ell |, |j - j'| \le N_{n}, \\ 0 &{}\quad \text {otherwise}, \end{array}\right. }\nonumber \\ \end{aligned}$$

which is the solution of (4.42).

#### Lemma 4.7

The operator $\Psi _n$ in (4.43), defined for any $(\omega , \zeta ) \in \Lambda _{n + 1}^\gamma$, satisfies, for any $s_0\le s\le S-\Sigma (\beta )$,

4.44 $$\begin{aligned} \begin{aligned} |\Psi _n|_{0, s}^{{\textrm{Lip}}(\gamma )}&\lesssim _{s} N_n^{4 \tau +2} \gamma ^{- 1} |{{\mathcal {R}}}_n|_{- M, s}^{{\textrm{Lip}}(\gamma )}, \\ |\Psi _n|_{0, s + M}^{{\textrm{Lip}}(\gamma )}&\lesssim _{s} N_n^{\tau _1} \gamma ^{- 1} |{{\mathcal {R}}}_n|_{- M, s}, \end{aligned} \end{aligned}$$

where $\tau _1>1$ is given in (4.28). Moreover, $\Psi _n$ is real and reversibility preserving.

#### Proof

To simplify notations, in this proof we drop the index *n*. Since $\lambda = (\omega , \zeta ) \in \Lambda _{n + 1}^\gamma$ (see (4.32)), one immediately gets the estimate

4.45 $$\begin{aligned} |{{\widehat{\Psi }}} (\ell )_j^{j'}| \lesssim \gamma ^{- 1} \langle \ell \rangle ^\tau | j |^\tau | j' |^\tau |{{\mathcal {R}}}(\ell )_j^{j'}|. \end{aligned}$$

For any $\lambda _1 = (\omega _1, \zeta _1), \lambda _2 = (\omega _2, \zeta _2) \in \Lambda _{n + 1}^\gamma$, we define $\delta _{\ell j j'}:={{\textrm{i}}}\, \omega \cdot \ell +\mu (j) - \mu (j')$. By (4.34), (4.32) one has

$$\begin{aligned} \begin{aligned} \Big | \frac{1}{ \delta _{\ell j j'}(\lambda _1)} - \frac{1}{\delta _{\ell j j'}(\lambda _2)} \Big |&\le \dfrac{|\delta _{\ell j j'}(\lambda _1) - \delta _{\ell j j'}(\lambda _2)|}{|\delta _{\ell j j'}(\lambda _1)| |\delta _{\ell j j'}(\lambda _2)|} \\&\lesssim \gamma ^{-2} \langle \ell \rangle ^{2 \tau + 1} \langle j - j' \rangle |j|^{2 \tau } |j'|^{2 \tau } |\lambda _1 - \lambda _2|. \end{aligned} \end{aligned}$$

The latter estimate (recall (4.43)) implies also that

4.46 $$\begin{aligned}&|{{\widehat{\Psi }}} (\ell )_j^{j'}(\lambda _1) - {{\widehat{\Psi }}} (\ell )_j^{j'}(\lambda _2)| \lesssim \langle \ell \rangle ^\tau |j|^\tau |j'|^\tau \gamma ^{- 1} |\widehat{{\mathcal {R}}}(\ell )_j^{j'}(\lambda _1) - \widehat{{\mathcal {R}}}(\ell )_j^{j'}(\lambda _2)| \nonumber \\&\quad + \langle \ell \rangle ^{2 \tau + 1} \langle j - j' \rangle |j|^{2 \tau } |j'|^{2 \tau } \gamma ^{- 2} |\widehat{{\mathcal {R}}}(\ell )_j^{j'}(\lambda _2)| |\lambda _1 - \lambda _2 |. \end{aligned}$$

Using that $\langle \ell , j - j' \rangle \le N$ and the elementary chain of inequalities $|j| \lesssim |j - j'| + |j'| \lesssim N + |j'| \lesssim N |j'|$, the estimates (4.45), (4.46) take the form

$$\begin{aligned} \begin{aligned} |{{\widehat{\Psi }}} (\ell )_j^{j'}|&\lesssim N^{2 \tau }\gamma ^{- 1} |j'|^{2 \tau } |{{\mathcal {R}}}(\ell )_j^{j'}|, \\ |{{\widehat{\Psi }}} (\ell )_j^{j'}(\lambda _1) - {{\widehat{\Psi }}} (\ell )_j^{j'}(\lambda _2)|&\lesssim N^{2 \tau } \gamma ^{- 1} |j'|^{2 \tau } |\widehat{{\mathcal {R}}}(\ell )_j^{j'}(\lambda _1) - \widehat{{\mathcal {R}}}(\ell )_j^{j'}(\lambda _2)| \\&\quad + N^{4 \tau + 2}\gamma ^{- 2} |j'|^{ 4 \tau } |\widehat{{\mathcal {R}}}(\ell )_j^{j'}(\lambda _2)| |\lambda _1 - \lambda _2 |. \end{aligned} \end{aligned}$$

Since $M > 4 \tau$ by (4.28), recalling Definition 2.5, the latter estimates imply that

$$\begin{aligned} \begin{aligned} |\Psi |_{0, s}^{\textrm{sup}}&\lesssim N^{2 \tau } \gamma ^{- 1} |{{\mathcal {R}}}|_{- M, s}^{\textrm{sup}}, \\ |\Psi |_{0, s}^{\textrm{lip}}&\lesssim N^{2 \tau } \gamma ^{- 1} |{{\mathcal {R}}}|_{- M, s}^{\textrm{lip}} + N^{4 \tau + 2} \gamma ^{- 2} |{{\mathcal {R}}}|_{- M, s}^{\textrm{sup}} \end{aligned} \end{aligned}$$

and similarly, using also that $\langle \ell , j - j' \rangle ^{M} \lesssim N^M$,

$$\begin{aligned} \begin{aligned} |\Psi |_{0, s + M}^{\textrm{sup}}&\lesssim N^{2 \tau + M} \gamma ^{- 1} |{{\mathcal {R}}}|_{- M, s}^{\textrm{sup}}, \\ |\Psi |_{0, s+ M}^{\textrm{lip}}&\lesssim N^{2 \tau + M} \gamma ^{- 1} |{{\mathcal {R}}}|_{- M, s}^{\textrm{lip}} + N^{4 \tau +M + 2} \gamma ^{- 2} |{{\mathcal {R}}}|_{- M, s}^{\textrm{sup}}. \end{aligned} \end{aligned}$$

Hence, we conclude the claimed bounds in (4.44). Finally, since ${{\mathcal {R}}}$ is real and reversible, by Lemma 2.10 and the properties (4.33) for $\mu (j)$, we deduce that $\Psi$ is real and reversibility preserving. $\square$

By Lemma 4.7 and the estimate in (4.36), we obtain, for any $s_0\le s\le S-\Sigma (\beta )$,

4.47 $$\begin{aligned} \begin{aligned} |\Psi _n|_{0, s}^{{\textrm{Lip}}(\gamma )}&\lesssim _{s} N_n^{4 \tau + 2} \gamma ^{- 1} |{{\mathcal {R}}}_n |_{- M, s}^{{\textrm{Lip}}(\gamma )} \lesssim _{s } N_n^{4 \tau + 2} N_{n - 1}^{- \alpha } \varepsilon \gamma ^{- 1}, \\ |\Psi _n|_{0, s + M}^{{\textrm{Lip}}(\gamma )} ,\,&\lesssim _{s} N_n^{\tau _1} \gamma ^{- 1} |{{\mathcal {R}}}_n |_{- M, s}^{{\textrm{Lip}}(\gamma )} \lesssim _{s } N_n^{\tau _1} N_{n - 1}^{- \alpha } \varepsilon \gamma ^{- 1}, \\ |\Psi _n|_{0, s + \beta }^{{\textrm{Lip}}(\gamma )}&\lesssim _{s,\beta } N_n^{4 \tau + 2} \gamma ^{- 1} |{{\mathcal {R}}}_n |_{-M, s + \beta }^{{\textrm{Lip}}(\gamma )} \lesssim _{s } N_n^{4 \tau + 2} N_{n - 1} \varepsilon \gamma ^{- 1}, \\ |\Psi _n|_{0, s + \beta + M}^{{\textrm{Lip}}(\gamma )}&\lesssim _{s,\beta } N_n^{\tau _1} \gamma ^{- 1} |{{\mathcal {R}}}_n |_{-M, s + \beta }^{{\textrm{Lip}}(\gamma )} \lesssim _{s } N_n^{\tau _1} N_{n - 1} \varepsilon \gamma ^{- 1}, \end{aligned}\nonumber \\ \end{aligned}$$

which are the estimates (4.38) at the step $n + 1$. By (4.28) and by the smallness condition (4.30), one has, for any $s_0\le s\le S-\Sigma (\beta )$ and for $N_0>0$ large enough,

4.48 $$\begin{aligned} |\Psi _n|_{0, s}^{{\textrm{Lip}}(\gamma )} \lesssim _s N_n^{4 \tau + 2} N_{n - 1}^{- \alpha } \le C(s) N_0^{4 \tau + 2} \varepsilon \gamma ^{- 1} \le \delta < 1 \end{aligned}$$

Therefore, by Lemma 2.6-(iv), $\Phi _n = {\textrm{Id}} + \Psi _n$ is invertible and

4.49 $$\begin{aligned} |\Phi _n^{- 1} - {\textrm{Id}}|_{0, s}^{{\textrm{Lip}}(\gamma )} \lesssim _{s} |\Psi _n|_{0, s}^{{\textrm{Lip}}(\gamma )}, \quad |\Phi _n^{- 1} - {\textrm{Id}}|_{0, s + \beta }^{{\textrm{Lip}}(\gamma )} \lesssim _{s} |\Psi _n|_{0, s +\beta }^{{\textrm{Lip}}(\gamma )}. \end{aligned}$$

Then, we define

4.50 $$\begin{aligned} \begin{aligned} {{\mathcal {L}}}_{n + 1}&:= \omega \cdot \partial _\varphi + {{\mathcal {D}}}_{n + 1} + {{\mathcal {R}}}_{n + 1}, \\ {{\mathcal {D}}}_{n + 1}&:= \zeta \cdot \nabla + {{\mathcal {Q}}}_{n + 1}, \qquad {{\mathcal {Q}}}_{n + 1}: = {{\mathcal {Q}}}_n + {{\mathcal {D}}}_{{{\mathcal {R}}}_n}, \\ {{\mathcal {R}}}_{n + 1}&:=\Pi _{N_n}^\bot {{\mathcal {R}}}_n + ( \Phi _n^{- 1} - {\textrm{Id}} ) \big ( {{\mathcal {D}}}_{{{\mathcal {R}}}_n} + \Pi _{N_n}^\bot {{\mathcal {R}}}_n\big )+ \Phi _n^{- 1} {{\mathcal {R}}}_n \Psi _n. \end{aligned} \end{aligned}$$

All the operators in (4.50) are defined for any $\lambda = (\omega ,\zeta ) \in \Lambda _{n + 1}^\gamma$. Since $\Psi _n, \Phi _n, \Phi _n^{- 1}$ are real and reversibility preserving and ${{\mathcal {D}}}_n, {{\mathcal {R}}}_n$ are real and reversible operators, one gets that ${{\mathcal {D}}}_{n + 1}$, ${{\mathcal {R}}}_{n + 1}$ are real and reversible operators. Moreover, by (4.40), (4.41), for $(\omega ,\zeta ) \in \Lambda ^\gamma _{n+1}$ one has the identity $\Phi _n^{-1} {\mathcal {L}}_n \Phi _n = {\mathcal {L}}_{n+1}$, which is (4.37) at the step $n+1$. By Definition 2.4 applied to ${{\mathcal {D}}}_{{{\mathcal {R}}}_n}$, one has that

4.51 $$\begin{aligned} \begin{aligned} {{\mathcal {Q}}}_{n + 1}&:= {{\mathcal {Q}}}_n + {{\mathcal {D}}}_{{{\mathcal {R}}}_n} = {\textrm{diag}}_{j \in {\mathbb {Z}}^2 \setminus \{ 0 \}} q_{n + 1}(j), \\ q_{n + 1}(j)&:= q_n(j) + \widehat{{\mathcal {R}}}_n(0)_j^j, \\ {{\mathcal {D}}}_{n + 1}&:= \zeta \cdot \nabla + {{\mathcal {Q}}}_{n + 1} = {\textrm{diag}}_{j \in {\mathbb {Z}}^2 \setminus \{ 0 \}} \mu _{n + 1}(j), \\ \mu _{n + 1}(j)&:= {{\textrm{i}}}\,\zeta \cdot j + q_{n + 1}(j). \end{aligned} \end{aligned}$$

The reality and the reversibility of ${{\mathcal {D}}}_{n + 1}$ and ${{\mathcal {Q}}}_{n + 1}$ imply that (4.33) is verified at the step $n + 1$. Moreover, by Lemma 2.6-(v)

$$\begin{aligned} \begin{aligned} | \mu _{n + 1}(j) - \mu _n(j)|^{{\textrm{Lip}}(\gamma )}&= | q_{n + 1}(j) - q_n(j)|^{{\textrm{Lip}}(\gamma )} \\&\le | \widehat{{\mathcal {R}}}_n(0)_j^j |^{{\textrm{Lip}}(\gamma )} \lesssim |{{\mathcal {R}}}_n|_{- M, s_0}^{{\textrm{Lip}}(\gamma )} \langle j \rangle ^{- M}. \end{aligned} \end{aligned}$$

Then, the estimate (4.36) implies (4.35) at the step $n + 1$. The estimate (4.34) at the step $n + 1$ follows, as usual, by a telescoping argument, using the fact that $\sum _{n \ge 0} N_{n - 1}^{- \alpha }$ is convergent since $\alpha > 0$ (see (4.28)). Now we prove the estimates (4.36) at the step $n + 1$. By (4.50), estimates (4.47), (4.48), (4.49), Lemma 2.6-(ii), (v) and Lemma 2.8, we get, for any $s_0\le s \le S-\Sigma (\beta )$,

$$\begin{aligned} \begin{aligned} |{{\mathcal {R}}}_{n + 1}|_{- M, s}^{{\textrm{Lip}}(\gamma )}&\lesssim _{s} N_n^{- \beta } |{{\mathcal {R}}}_n|_{- M, s + \beta }^{{\textrm{Lip}}(\gamma )} + N_n^{\tau _1} \gamma ^{- 1} |{{\mathcal {R}}}_n|_{- M, s_0}^{{\textrm{Lip}}(\gamma )} |{{\mathcal {R}}}_n|_{- M, s}^{{\textrm{Lip}}(\gamma )} , \\ |{{\mathcal {R}}}_{n + 1}|_{- M, s + \beta }^{{\textrm{Lip}}(\gamma )}&\lesssim _{s} |{{\mathcal {R}}}_n|_{- M, s +\beta }^{{\textrm{Lip}}(\gamma )} + N_{n}^{\tau _1} \gamma ^{-1}\big ( |{{\mathcal {R}}}_n|_{- M, s_0}^{{\textrm{Lip}}(\gamma )} |{{\mathcal {R}}}_n|_{- M, s+\beta }^{{\textrm{Lip}}(\gamma )}\\&+ |{{\mathcal {R}}}_n|_{- M, s}^{{\textrm{Lip}}(\gamma )} |{{\mathcal {R}}}_n|_{- M, s_0+\beta }^{{\textrm{Lip}}(\gamma )} \big ) . \end{aligned}\nonumber \\ \end{aligned}$$

By the induction estimate (4.36), the definition of the constants in (4.28) and the smallness condition in (4.30), taking $N_0 = N_0(S, \tau )> 0$ large enough, we obtain the estimates (4.36) at the step $n + 1$.

Proof of $\mathbf{(S2)}_{n + 1}$. It remains to construct a Lipschitz extension for the eigenvalues $\mu _{n + 1}(j,\,\cdot \,): \Lambda _{n + 1}^\gamma \rightarrow {{\textrm{i}}}\,{\mathbb {R}}$. By the induction hypothesis, there exists a Lipschitz extension of $\mu _n(j,\lambda )$, denoted by ${{\widetilde{\mu }}}_{n}(j,\lambda )$ to the whole set $DC(\gamma , \tau )$ that satisfies $\mathbf{(S2)}_n$. By (4.51), we have $\mu _{n + 1}(j) = \mu _n(j) + r_n(j)$ where $r_n(j)=r_n(j,\lambda ):= \widehat{{\mathcal {R}}}_n(0;\lambda )_j^j$ satisfies $|r_n(j)|^{{\textrm{Lip}}(\gamma )} \lesssim N_{n - 1}^{- \alpha } |j|^{- M} \varepsilon$. By the reversibility and the reality of ${{\mathcal {R}}}_n$, we have $r_n(j) = - r_n(- j)=\overline{r_n(-j)}$, implying that $r_n(j) \in {{\textrm{i}}}\, {\mathbb {R}}$. Hence by the Kirszbraun Theorem (see Lemma M.5 [36]) there exists a Lipschitz extension ${{\widetilde{r}}}_n(j,\,\cdot \,): DC(\gamma , \tau ) \rightarrow {{\textrm{i}}}{\mathbb {R}}$ of $r_n(j,\,\cdot \,): \Lambda _{n + 1}^\gamma \rightarrow {{\textrm{i}}}\,{\mathbb {R}}$ satisfying $|{{\widetilde{r}}}_n(j)|^{{\textrm{Lip}}(\gamma )} \lesssim |r_n(j)|^{{\textrm{Lip}}(\gamma )} \lesssim N_{n - 1}^{- \alpha } |j|^{- M} \varepsilon$. The claimed statement then follows by defining ${{\widetilde{\mu }}}_{n + 1}(j):= {{\widetilde{\mu }}}_n(j) + {{\widetilde{r}}}_n(j)$. $\square$

### KAM Reducibility: Convergence

In this section we prove that the KAM reducibility scheme for the operator ${\mathcal {L}}_{e}^{(2)}$, whose iterative step is described in Proposition 4.6, is convergent under the smallness condition (4.30) with the final operator being diagonal with purely imaginary eigenvalues.

#### Lemma 4.8

For any $j \in {\mathbb {Z}}^2 \setminus \{ 0 \}$, the sequence $\{ {{\widetilde{\mu }}}_n(j)= {{\textrm{i}}}\, \zeta \cdot j + q_n(j) \}_{n\in {\mathbb {N}}}$ converges to some limit

$$\begin{aligned} \mu _\infty (j) = {{\textrm{i}}}\,\zeta \cdot j + q_\infty (j), \quad \mu _\infty (j)= \mu _\infty (j,\,\cdot \,):DC(\gamma ,\tau )\rightarrow {{\textrm{i}}}\,{\mathbb {R}}, \end{aligned}$$

satisfying the following estimates

4.52 $$\begin{aligned} \begin{aligned}&| \mu _\infty (j) - {{\widetilde{\mu }}}_n(j) |^{{\textrm{Lip}}(\gamma )} = | q_\infty (j) - {{\widetilde{q}}}_n(j) |^{{\textrm{Lip}}(\gamma )} \lesssim N_{n - 1}^{- \alpha } |j|^{- M} \varepsilon , \\&| q_\infty (j)|^{{\textrm{Lip}}(\gamma )} \lesssim |j|^{- 1} \varepsilon . \end{aligned} \end{aligned}$$

#### Proof

By Proposition 4.6-$\mathbf{(S2)}_n$, we have that the sequence $\{{{\widetilde{\mu }}}_n(j,\lambda )\}_{n\in {\mathbb {N}}}\subset {{\textrm{i}}}\,{\mathbb {R}}$ is Cauchy on the closed set $DC(\gamma ,\tau )$, therefore it is convergent for any $\lambda \in DC(\gamma ,\tau )$. The estimate (4.52) follows by a telescoping argument with the estimate (4.39). $\square$

We define the set $\Lambda _\infty ^\gamma$ of the non-resonance conditions for the final eigenvalues as

4.53 $$\begin{aligned} \Lambda _\infty ^\gamma&:= \Big \{ \lambda \in DC(\gamma ,\tau ): |{{\textrm{i}}}\,\omega \cdot \ell + \mu _\infty (j) - \mu _\infty (j')| \ge \frac{2\gamma }{\langle \ell \rangle ^\tau | j |^\tau | j' |^\tau }, \nonumber \\&\qquad \forall \ell \in {\mathbb {Z}}^d, \ \ j, j' \in {\mathbb {Z}}^2 \setminus \{ 0 \}, \ \ (\ell , j, j') \ne (0, j, j)\Big \}. \end{aligned}$$

#### Lemma 4.9

We have $$\Lambda _\infty ^\gamma \subseteq \cap _{n \ge 0} \, \Lambda _n^\gamma $$.

#### Proof

We prove by induction that $\Lambda _\infty ^\gamma \subseteq \Lambda _n^\gamma$ for any integer $n \ge 0$. The statement is trivial for $n=0$, since $\Lambda _0^\gamma := DC(\gamma ,\tau )$ (see Proposition 4.6). We now assume by induction that $\Lambda _\infty ^\gamma \subseteq \Lambda _n^\gamma$ for some $n \ge 0$ and we show that $\Lambda _\infty ^\gamma \subseteq \Lambda _{n + 1}^\gamma$. Let $\lambda \in \Lambda _\infty ^\gamma$, $\ell \in {\mathbb {Z}}^d$, $j, j' \in {\mathbb {Z}}^2 {\setminus } \{ 0 \}$, with $(\ell , j, j') \ne (0, j, j)$ and $|\ell |, |j - j'| \le N_n$. By (4.52), (4.53), we compute

$$\begin{aligned} \begin{aligned} |{{\textrm{i}}}\,\omega \cdot \ell + \mu _n(j) - \mu _n(j')|&\ge |{{\textrm{i}}}\,\omega \cdot \ell + \mu _\infty (j) - \mu _\infty (j')| - |\mu _\infty (j) - \mu _n(j)| \\&\quad - |\mu _\infty (j') - \mu _n(j')| \\&\ge \frac{2\gamma }{\langle \ell \rangle ^\tau | j |^\tau | j' |^\tau } - C N_{n - 1}^{- \alpha } \varepsilon \Big ( |j|^{- M} + |j'|^{- M} \Big ) \\&\ge \frac{\gamma }{\langle \ell \rangle ^\tau | j |^\tau | j' |^\tau } \end{aligned} \end{aligned}$$

for some positive constant $C>0$, provided

4.54 $$\begin{aligned} C \varepsilon \gamma ^{- 1}\langle \ell \rangle ^\tau | j |^\tau | j' |^\tau \Big ( |j|^{- M} + |j'|^{- M} \Big ) \le 1. \end{aligned}$$

Using that $|\ell |, |j - j'| \le N_n$ and the chain of inequalities

$$\begin{aligned} \begin{aligned}&|j| \le |j'| + |j - j'| \le |j'| + N_n \lesssim N_n |j'|, \end{aligned} \end{aligned}$$

we deduce that, for some $C_0>0$ and recalling that $M > 4 \tau$ by (4.28),

$$\begin{aligned} \langle \ell \rangle ^\tau | j |^\tau | j' |^\tau \Big ( |j|^{- M} + |j'|^{- M} \Big ) \le C_0 N_n^{2 \tau }. \end{aligned}$$

Therefore, (4.54) is verified provided $C C_0 N_n^{2 \tau } \varepsilon \gamma ^{- 1} \le 1\,.$ The latter inequality is implied by the smallness condition (4.30). We conclude that $\lambda = (\omega , \zeta ) \in \Lambda _{n + 1}^\gamma$. $\square$

Now we define the sequence of invertible maps

4.55 $$\begin{aligned} {{\widetilde{\Phi }}}_n:= \Phi _0 \circ \Phi _1 \circ \ldots \circ \Phi _n, \quad n \in {\mathbb {N}}. \end{aligned}$$

#### Proposition 4.10

Let $S > s_0 + \Sigma (\beta )$. There exists $\delta := \delta (S, \tau , d) > 0$ such that, if (3.2) (4.30) are verified, then the following holds. For any $\lambda = (\omega , \zeta ) \in \Lambda _\infty ^\gamma$, the sequence $({{\widetilde{\Phi }}}_n)_{n\in {\mathbb {N}}}$ converges in norm $| \cdot |_{0, s}^{{\textrm{Lip}}(\gamma )}$ to an invertible map $\Phi _\infty$, satisfying, for any $s_0\le s\le S-\Sigma (\beta )$,

4.56 $$\begin{aligned} \begin{aligned}&|\Phi _\infty ^{\pm 1} - {{\widetilde{\Phi }}}_n^{\pm 1}|_{0, s}^{{\textrm{Lip}}(\gamma )} \lesssim _{s} N_{n + 1}^{4 \tau + 2} N_n^{- \alpha } \varepsilon \gamma ^{- 1} , \quad |\Phi _\infty ^{\pm 1} - {\textrm{Id}}|_{0, s}^{{\textrm{Lip}}(\gamma )} \lesssim _{s} \varepsilon \gamma ^{- 1} . \end{aligned} \end{aligned}$$

The operators $\Phi _\infty ^{\pm 1}: H^s_0 \rightarrow H^s_0$ are real and reversibility preserving. Moreover, for any $\lambda \in \Lambda _\infty ^\gamma$, one has

4.57 $$\begin{aligned} {{\mathcal {L}}}_{ e}^{(\infty )}:= \Phi _\infty ^{- 1} {{\mathcal {L}}}_e^{(2)} \Phi _\infty = \omega \cdot \partial _\varphi + {{\mathcal {D}}}_\infty , \quad {{\mathcal {D}}}_\infty := {\textrm{diag}}_{j \in {\mathbb {Z}}^2 \setminus \{ 0 \}} \mu _\infty (j)\nonumber \\ \end{aligned}$$

where the operator ${{\mathcal {L}}}_e^{(2)}$ is given in (4.16)–(4.27) and the final eigenvalues $\mu _\infty (j)$ are given in Lemma 4.8.

#### Proof

The existence of the invertible map $\Phi _\infty ^{\pm 1}$ and the estimates (4.56) follow by (4.38), (4.55), arguing as in Corollary 4.1 in [3]. By (4.55), Lemma 4.9 and Proposition 4.6, one has ${{\widetilde{\Phi }}}_n^{- 1} {{\mathcal {L}}}_0 {{\widetilde{\Phi }}}_n = \omega \cdot \partial _\varphi + {{\mathcal {D}}}_n + {{\mathcal {R}}}_n$ for all $n \ge 0$. The claimed statement then follows by passing to the limit as $n \rightarrow \infty$, by using (4.36), (4.56) and Lemma 4.8. $\square$

## Inversion of the Linearized Navier–Stokes Operator ${\mathcal {L}}_\nu$

The main purpose of this section is to prove the invertibility of the operators ${\mathcal {L}}_\nu$ in (3.3), for any value of the viscosity $\nu > 0$ with a smallness condition on $\varepsilon$ which is independent of $\nu$. We also prove the invertibility of ${\mathcal {L}}_{e}$, which we shall use to construct an approximate solution up to order $O(\nu ^2)$ in Sect. 6. We use the normal form reduction implemented in Sect. 4. First, we recollect all the terms. By (4.16) and by Lemma 4.10, for any $\lambda \in \Lambda _\infty ^\gamma$, the operator ${\mathcal {L}}^{(2)}_\nu$ in (4.16) is conjugated to

5.1 $$\begin{aligned} \begin{aligned} {{\mathcal {L}}}^{(\infty )}_\nu&:= \Phi _\infty ^{- 1} {{\mathcal {L}}}^{(2)}_\nu \Phi _\infty = \Phi _\infty ^{- 1} {{\mathcal {L}}}_e^{(2)} \Phi _\infty - \nu \Phi _\infty ^{- 1} \Delta \Phi _\infty + \Phi _\infty ^{- 1} {{\mathcal {R}}}_\nu ^{(2)} \Phi _\infty \\&= {{\mathcal {L}}}_{e}^{(\infty )} - \nu \Delta + {{\mathcal {R}}}_{\nu }^{(\infty )}, \\ {{\mathcal {R}}}_{\nu }^{(\infty )}&:= - \nu \big ( \Delta (\Phi _\infty - {\textrm{Id}}) + (\Phi _\infty ^{- 1} - {\textrm{Id}}) \Delta \Phi _\infty \big ) + \Phi _\infty ^{- 1} {{\mathcal {R}}}_{\nu }^{(2)} \Phi _\infty \end{aligned} \end{aligned}$$

By the estimates (4.17), (4.56), using that $|\Delta |_{2, s} \le 1$, for any $s > s_0$ and for $S > s_0 + \Sigma (\beta ) + 2$, there exists $\delta := \delta (S, \tau , d) \in (0, 1)$ such that if (3.2), (4.2) hold, by applying Lemma 2.6-(ii), one gets

5.2 $$\begin{aligned} |{{\mathcal {R}}}_{\nu }^{(\infty )}|^{{\textrm{Lip}}(\gamma )}_{2, s} \lesssim _s \varepsilon \gamma ^{- 1}\,\nu , \quad \forall s_0 \le s \le S - \Sigma (\beta ) - 2. \end{aligned}$$

### Lemma 5.1

(Inversion of ${{\mathcal {L}}}_\nu ^{(\infty )}$). For any $S > s_0 + \Sigma (\beta ) + 4$, there exists $\delta := \delta (s, \tau , d) \in (0,1)$ small enough such that, if (3.2) holds and $\varepsilon \gamma ^{- 1} \le \delta$, the operator ${{\mathcal {L}}}_\nu ^{(\infty )}$ is invertible for any $\nu > 0$ with bounded inverse $({{\mathcal {L}}}_\nu ^{(\infty )})^{- 1} \in {{\mathcal {B}}}(H^s_0)$ for any $s_0\le s\le S-\Sigma (\beta ) - 4$. Moreover, the inverse operator $({\mathcal {L}}_{\nu }^{(\infty )})^{-1}$ is smoothing of order two, that is $({{\mathcal {L}}}_\nu ^{(\infty )})^{- 1} (- \Delta ) \in {{\mathcal {B}}}(H^s_0)$, with $\Vert ({{\mathcal {L}}}_\nu ^{(\infty )})^{- 1} (- \Delta )\Vert _{{{\mathcal {B}}}(H^s_0)} \lesssim _s \nu ^{- 1}$.

### Proof

By (5.1) and (4.57), we write

5.3 $$\begin{aligned} \begin{aligned}&{{\mathcal {L}}}_\nu ^{(\infty )} = {\mathtt L}_\nu ^{(\infty )} + {{\mathcal {R}}}_{ \nu }^{(\infty )}, \qquad {\mathtt L}_\nu ^{(\infty )}:= {\mathcal {L}}_{e}^{(\infty )} -\nu \Delta = \omega \cdot \partial _\varphi + {{\mathcal {D}}}_\infty - \nu \Delta . \end{aligned} \end{aligned}$$

By Lemma 4.10, one has that ${\mathtt L}_\nu ^{(\infty )}$ is a diagonal operator with eigenvalues ${{\textrm{i}}}\,\omega \cdot \ell + \mu _\infty (j) + \nu |j|^2$, for $\ell \in {\mathbb {Z}}^d, j \in {\mathbb {Z}}^2 {\setminus } \{ 0 \}$. By Lemma 4.8, $\mu _\infty (j) \in {{\textrm{i}}}{\mathbb {R}}$ is purely imaginary. Therefore, for any $\ell \in {\mathbb {Z}}^d$, $j \in {\mathbb {Z}}^2 {\setminus } \{ 0 \}$, we obtain a lower bound of the eigenvalues for any value of the parameters $\lambda = (\omega , \zeta )$ as follows:

5.4 $$\begin{aligned} \begin{aligned} |{{\textrm{i}}}\,\omega \cdot \ell + \mu _\infty (j) + \nu |j|^2|&\ge \big |{\textrm{Re}}\big ( {{\textrm{i}}}\,\omega \cdot \ell + \mu _\infty (j) + \nu |j|^2\big ) \big | = \nu |j|^2. \end{aligned} \end{aligned}$$

The latter bound implies that the operator ${\mathtt L}_\nu ^{(\infty )}$ in (5.3) is invertible for any $\nu >0$, with inverse given by

$$\begin{aligned} ({\mathtt L}_\nu ^{(\infty )})^{- 1} h(\varphi , x) = \sum _{{\begin{array}{c} \ell \in {\mathbb {Z}}^d \\ j \in {\mathbb {Z}}^2 \setminus \{ 0 \} \end{array}}} \dfrac{{{\widehat{h}}}(\ell , j)}{{{\textrm{i}}}\,\omega \cdot \ell + \mu _\infty (j) +\nu |j|^2} \,e^{{{\textrm{i}}}\ell \cdot \varphi } e^{{{\textrm{i}}}j \cdot x}, \quad h \in H^s_0. \end{aligned}$$

Recalling that $\langle j \rangle = {\textrm{max}}\{ 1, |j| \}=|j|$ for $j \ne 0$, by the smoothing term from the small divisor (5.4), one has, for any $s_0\le s \le S-\Sigma (\beta )$,

$$\begin{aligned} \begin{aligned} \Vert ( {\mathtt L}_\nu ^{(\infty )})^{- 1} (- \Delta ) h \Vert _s^2&= \sum _{{\begin{array}{c} \ell \in {\mathbb {Z}}^d \\ j \in {\mathbb {Z}}^2 \setminus \{ 0 \} \end{array}}} \langle \ell , j \rangle ^{2 s} \frac{| j |^{4}}{|{{\textrm{i}}}\,\omega \cdot \ell + \mu _\infty (j) + \nu |j|^2|^2} |{{\widehat{h}}}(\ell , j)|^2 \\&\le \nu ^{- 2} \sum _{{\begin{array}{c} \ell \in {\mathbb {Z}}^d \\ j \in {\mathbb {Z}}^2 \setminus \{ 0 \} \end{array}}} \langle \ell , j \rangle ^{2 s} |{{\widehat{h}}}(\ell , j)|^2 = \nu ^{- 2} \Vert h \Vert _s^2 \end{aligned} \end{aligned}$$

implying that

5.5 $$\begin{aligned} \Vert ({\mathtt L}_\nu ^{(\infty )})^{- 1} (- \Delta ) \Vert _{{{\mathcal {B}}}(H^s_0)} \le \nu ^{- 1} \quad \text {and } \quad \Vert ({\mathtt L}_\nu ^{(\infty )})^{- 1} \Vert _{{{\mathcal {B}}}(H^s_0)} \le \nu ^{- 1}. \end{aligned}$$

We write the operator ${{\mathcal {L}}}_\nu ^{(\infty )}$ in (5.3) as ${{\mathcal {L}}}_\nu ^{(\infty )} = {\mathtt L}_\nu ^{(\infty )} \big ( {\textrm{Id}} + ({\mathtt L}_\nu ^{(\infty )})^{- 1} {{\mathcal {R}}}_{ \nu }^{(\infty )} \big )$. By the estimates (5.5), (5.2) and Lemma 2.6-(i), (ii), one gets, for any $s_0\le s \le S-\Sigma (\beta ) - 4$,

$$\begin{aligned} \begin{aligned} \Vert ({\mathtt L}_\nu ^{(\infty )})^{- 1} {{\mathcal {R}}}_{ \nu }^{(\infty )} \Vert _{{{\mathcal {B}}}(H^s_0)}&\le \Vert ({\mathtt L}_\nu ^{(\infty )})^{- 1} (- \Delta ) \Vert _{{{\mathcal {B}}}(H^s_0)} \Vert (- \Delta )^{- 1} {{\mathcal {R}}}_{ \nu }^{(\infty )} \Vert _{{{\mathcal {B}}}(H^s_0)} \\&\le \nu ^{- 1} |(- \Delta )^{- 1} {{\mathcal {R}}}_{ \nu }^{(\infty )} |_{0, s} \lesssim \nu ^{- 1} |{{\mathcal {R}}}_{ \nu }^{(\infty )}|_{2, s + 2} \\&\lesssim _s \nu ^{- 1} \nu \varepsilon \gamma ^{- 1} \lesssim _s \varepsilon \gamma ^{- 1}. \end{aligned} \end{aligned}$$

Hence, having $\varepsilon \gamma ^{- 1} \le \delta < 1$ small enough (independent of the viscosity $\nu > 0$), the operator ${\textrm{Id}} + ({\mathtt L}_\nu ^{(\infty )})^{- 1} {{\mathcal {R}}}_{ \nu }^{(\infty )}$ is invertible by Neumann series, uniformly with respect to the viscosity parameter $\nu > 0$ and $\big \Vert \big ( {\textrm{Id}} + {\mathtt L}_\infty ^{- 1} {{\mathcal {R}}}_{\infty , \nu } \big )^{- 1} \big \Vert _{{{\mathcal {B}}}(H^s_0)} \lesssim _s 1$. Together with the estimate (5.5), one deduces that $({{\mathcal {L}}}_\nu ^{(\infty )})^{- 1} = \big ( {\textrm{Id}} + ({\mathtt L}_\nu ^{(\infty )})^{- 1} {{\mathcal {R}}}_{\nu }^{(\infty )} \big )^{- 1} ({\mathtt L}_\nu ^{(\infty )})^{- 1}$, with

$$\begin{aligned} \begin{aligned} \Vert ({{\mathcal {L}}}_\nu ^{(\infty )})^{- 1} (- \Delta ) \Vert _{{{\mathcal {B}}}(H^s_0)}&\lesssim _s \nu ^{- 1}. \end{aligned} \end{aligned}$$

The claimed statement has then been proved. $\square$

We now deal with the inversion of the linearized operator ${\mathcal {L}}_{\nu }$ in (3.3). By Propositions 4.1, 4.4, Lemma 4.10 and recalling the definition of the set $\Lambda _\infty ^\gamma$ in (4.53), one has that, for any $\lambda \in \Lambda _\infty ^\gamma$,

5.6 $$\begin{aligned} \begin{aligned}&{{\mathcal {L}}}_\nu = {{\mathcal {W}}}_\infty {{\mathcal {L}}}_\nu ^{(\infty )} {{\mathcal {W}}}_\infty ^{- 1}, \quad {{\mathcal {L}}}_e = {{\mathcal {W}}}_\infty {{\mathcal {L}}}_{e}^{(\infty )} {{\mathcal {W}}}_\infty ^{- 1} , \quad {{\mathcal {W}}}_\infty := {{\mathcal {A}}}_\bot {{\mathcal {B}}} \Phi _\infty , \end{aligned} \end{aligned}$$

where the invertible maps ${\mathcal {A}}_\perp$, ${\mathcal {B}}$ and $\Phi _\infty$ are provided in Propositions 4.3, 4.4 and 4.10, respectively. Furthermore, by Lemma 5.1, the inverse of the operator ${{\mathcal {L}}}_\nu$ is given by ${{\mathcal {L}}}_\nu ^{- 1} = {{\mathcal {W}}}_\infty ({{\mathcal {L}}}_\nu ^{(\infty )})^{- 1} {{\mathcal {W}}}_\infty ^{- 1}$. We need to estimate $\Vert {{\mathcal {L}}}_\nu ^{- 1} (- \Delta ) \Vert _{{{\mathcal {B}}}(H^s_0)}$. First, we need some auxiliary lemmata. Let us denote

$$\begin{aligned} \langle D \rangle ^{2}:= {\textrm{Id}} - \Delta , \quad \langle D \rangle ^{-2}:= ({\textrm{Id}} - \Delta )^{-1} . \end{aligned}$$

### Lemma 5.2

There exists $\Sigma _1(\beta ) > \Sigma (\beta )$ (where $\Sigma (\beta )$ is given in (4.28)) large enough such that, for any $S > s_0 + \Sigma _1(\beta )$, there exists $\delta := \delta (S, \tau , d) \in (0, 1)$ such that, if (3.2) is fulfilled and $\varepsilon \gamma ^{- 1} \le \delta$, the invertible map ${{\mathcal {A}}}$ given in Proposition 4.1 satisfies $\langle D \rangle ^{- 2} {{\mathcal {A}}}^{\pm 1} \langle D \rangle ^2 \in {{\mathcal {B}}}(H^s)$ for any $s_0\le s \le S-\Sigma _1(\beta )$, with estimate $\Vert \langle D \rangle ^{- 2} {{\mathcal {A}}}^{\pm 1} \langle D \rangle ^2 \Vert _{{{\mathcal {B}}}(H^s)} \lesssim _s 1$.

### Proof

We prove the claim for $\langle D \rangle ^{- 2} {{\mathcal {A}}} \langle D \rangle ^2$. The proof for $\langle D \rangle ^{- 2} {{\mathcal {A}}}^{-1} \langle D \rangle ^2$ is analogous and we omit it. For any $\tau \in [- 1, 1]$, we define the operator ${{\mathcal {A}}}(\tau )$ by

$$\begin{aligned} {{\mathcal {A}}}(\tau ): u_0(\varphi , x) \mapsto u_0(\varphi , x + \tau \alpha (\varphi , x)). \end{aligned}$$

By Proposition (4.1), for any $\tau \in [- 1, 1]$, the map ${{\mathcal {A}}}(\tau )$ is invertible on $H^s({\mathbb {T}}^{d + 2})$ and

5.7 $$\begin{aligned} \Vert {{\mathcal {A}}}(\tau )^{\pm 1} \Vert _{{{\mathcal {B}}}(H^s)} \lesssim _s 1, \quad \forall \tau \in [- 1 ,\, 1]. \end{aligned}$$

For some $\Sigma _1(\beta ) \gg 0$ sufficiently large and for any $S > s_0 + \Sigma _1(\beta )$, we can apply the estimate (4.3) with $s + 4$ instead of *s*, obtaining, for any $s_0\le s \le S-\Sigma _1(\beta )$,

$$\begin{aligned} \Vert \alpha \Vert _{s + 4}, \Vert \breve{\alpha } \Vert _{s + 4} \lesssim _s \varepsilon \gamma ^{- 1}. \end{aligned}$$

A direct calculation shows that ${{\mathcal {A}}}(\tau )$ solves

$$\begin{aligned} {\left\{ \begin{array}{ll} \partial _\tau {{\mathcal {A}}}(\tau ) = b(\tau , \varphi , x) \cdot \nabla {{\mathcal {A}}}(\tau ), \\ {{\mathcal {A}}}(0) = {\textrm{Id}}, \end{array}\right. } \text {where} \quad b(\tau , \varphi , x):= ({\textrm{Id}} + \tau D_{x} \alpha (\varphi , x))^{- 1} \alpha (\varphi , x). \end{aligned}$$

Note that, since $\Vert \alpha \Vert _{s + 1} \lesssim _s \varepsilon \gamma ^{- 1} \le \delta < 1$, the $2 \times 2$ matrix ${\textrm{Id}} + D_{x} \alpha (\varphi , x)$ is invertible by Neumann series and by using the tame estimate in (2.6), one has that $\Vert ({\textrm{Id}} + \tau D_{x} \alpha (\varphi , x))^{- 1} \Vert _s \lesssim _s 1$. Hence

5.8 $$\begin{aligned} \Vert b(\tau , \cdot ) \Vert _s \lesssim _s \Vert \alpha \Vert _{s + 1} \lesssim _s \varepsilon \gamma ^{- 1}, \quad \text {uniformly in} \quad \tau \in [- 1, 1]. \end{aligned}$$

Define

$$\begin{aligned} \Phi (\tau ):= \langle D \rangle ^{- 2} {{\mathcal {A}}}(\tau ) \langle D \rangle ^2, \quad \tau \in [- 1, 1]. \end{aligned}$$

Clearly $\Phi (0, \varphi ) = {\textrm{Id}}$ and, by a direct computation,

$$\begin{aligned} \begin{aligned}&\partial _\tau \Phi (\tau ) = b (\tau , \varphi , x) \cdot \nabla \Phi (\tau ) + {{\mathcal {R}}}(\tau ) \Phi (\tau ) ,\\&{{\mathcal {R}}}(\tau ):= [\langle D \rangle ^{- 2},b (\tau , \varphi , x) \cdot \nabla ] \langle D \rangle ^{2} . \end{aligned} \end{aligned}$$

By variation of the constants, we write $\Phi (\tau ) = {{\mathcal {A}}}(\tau ) {{\mathcal {M}}}(\tau )$, where ${{\mathcal {M}}}(\tau )$ solves

5.9 $$\begin{aligned} {\left\{ \begin{array}{ll} \partial _\tau {{\mathcal {M}}}(\tau ) = {{\mathcal {Q}}}(\tau ) {{\mathcal {M}}}(\tau ) ,\\ {{\mathcal {M}}}(0) = {\textrm{Id}}, \end{array}\right. } \quad \text {with} \quad {{\mathcal {Q}}}(\tau ):= {{\mathcal {A}}}(\tau )^{- 1} {{\mathcal {R}}}(\tau ) {{\mathcal {A}}}(\tau ). \end{aligned}$$

First, we estimate ${\mathcal {R}}(\tau )$. We claim that

5.10 $$\begin{aligned} \sup _{\tau \in [- 1, 1]} \Vert {{\mathcal {R}}}(\tau )\Vert _{{{\mathcal {B}}}(H^s)} \lesssim _s \varepsilon \gamma ^{- 1}. \end{aligned}$$

By a direct calculation, the matrix representation of the operator ${{\mathcal {R}}}(\tau )$ is given by

5.11 $$\begin{aligned} \widehat{{\mathcal {R}}}(\tau , \ell )_j^{j'} = \Big (\dfrac{\langle j' \rangle ^2 - \langle j \rangle ^2}{\langle j \rangle ^2 \langle j' \rangle ^2} \Big ) \langle j' \rangle ^2\, {{\textrm{i}}}\,j' \cdot {{\widehat{b}}}(\tau , \ell , j - j'), \quad (\ell , j, j') \in {\mathbb {Z}}^d \times {\mathbb {Z}}^2 \times {\mathbb {Z}}^2.\nonumber \\ \end{aligned}$$

Note that $\widehat{{\mathcal {R}}}(\tau , \ell )_j^{j'} = 0$ when $j'=0$. Therefore, we can always consider $j' \ne 0$, implying that $\langle j' \rangle = |j'|$. One then has

$$\begin{aligned} \begin{aligned} \langle j' \rangle ^2 - \langle j \rangle ^2&= |j'|^2 - \langle j \rangle ^2 = |j + j' - j|^2 - \langle j \rangle ^2 \\&= |j|^2 + 2 j \cdot (j - j') + |j - j'|^2 - \langle j \rangle ^2, \\ \end{aligned} \end{aligned}$$

which implies that $|\langle j' \rangle ^2 - \langle j \rangle ^2 | \lesssim \langle j - j' \rangle ^2 \langle j \rangle$. This implies that the coefficients in (5.11) are estimated as

$$\begin{aligned} \begin{aligned} |\widehat{{\mathcal {R}}}(\tau , \ell )_j^{j'} |&\lesssim \dfrac{ \langle j - j' \rangle ^2 \langle j' \rangle }{\langle j \rangle }|{{\widehat{b}}}(\tau , \ell , j - j')| \\&\lesssim \dfrac{ \langle j - j' \rangle ^2 (\langle j\rangle + \langle j - j'\rangle )}{\langle j \rangle }|{{\widehat{b}}}(\tau , \ell , j - j')| \lesssim \langle j - j' \rangle ^3 |{{\widehat{b}}}(\tau , \ell , j - j')| \end{aligned} \end{aligned}$$

Then, by (2.11), (2.13) and (5.8), one gets

$$\begin{aligned} |{{\mathcal {R}}}(\tau )|_{0, s} \lesssim \Vert b(\tau , \cdot ) \Vert _{s + 3} \lesssim _s \Vert \alpha \Vert _{s + 4} \lesssim _s \varepsilon \gamma ^{- 1}. \end{aligned}$$

We conclude the claimed bound (5.10) by Lemma 2.6-(i).

We now estimate $\Phi (\tau )$. By the estimates (5.7), (5.10), one gets that ${{\mathcal {Q}}}(\tau )$ satisfies

$$\begin{aligned} \sup _{\tau \in [- 1, 1]} \Vert {{\mathcal {Q}}}(\tau ) \Vert _{{{\mathcal {B}}}(H^s)} \lesssim _s \varepsilon \gamma ^{- 1}. \end{aligned}$$

Hence, by (5.9), ${{\mathcal {M}}}(\tau )$ is the propagator associated to a linear vector field which is bounded on $H^s({\mathbb {T}}^{d + 2})$ uniformly with respect to $\tau$. By standard arguments, we get

$$\begin{aligned} \sup _{\tau \in [- 1, 1]} \Vert {{\mathcal {M}}}(\tau ) - {\textrm{Id}} \Vert _{{{\mathcal {B}}}(H^s)} \lesssim _s \varepsilon \gamma ^{-1}. \end{aligned}$$

Since $\Phi (\tau ) = {{\mathcal {A}}}(\tau ) {{\mathcal {M}}}(\tau )$, the latter estimate together with (5.7) and $\varepsilon \gamma ^{- 1} \le \delta 1$ implies the claimed bound for $\Phi (1)\equiv \langle D \rangle ^{-2} {\mathcal {A}}\langle D \rangle ^{2}$. $\square$

### Lemma 5.3

There exists $\Sigma _2(\beta ) > \Sigma _1(\beta )$ large enough (where $\Sigma _1(\beta )$ is given in Lemma 5.2) such that, for any $S > s_0 + \Sigma _2(\beta )$, there exists $\delta := \delta (S, \tau , d) \in (0, 1)$ such that, if (3.2) is fulfilled and $\varepsilon \gamma ^{- 1} \le \delta$, the maps ${{\mathcal {W}}}_\infty ^{\pm 1}: H^s_0 \rightarrow H^s_0$ in (5.6) are bounded for any $s_0\le s\le S-\Sigma _2(\beta )$ with estimates $\Vert {{\mathcal {W}}}_\infty ^{\pm 1} \Vert _{{{\mathcal {B}}}(H^s_0)} \lesssim _s 1$. Moreover, we have $\Vert (- \Delta )^{- 1}{{\mathcal {W}}}_\infty ^{\pm 1} (- \Delta ) \Vert _{{{\mathcal {B}}}(H^s_0)} \lesssim _s 1$.

### Proof

We prove the claimed bound for $(- \Delta )^{- 1}{{\mathcal {W}}}_\infty (- \Delta )$. The other bounds follow similarly and we omit their proof. By (5.6), one has

$$\begin{aligned} \begin{aligned} (- \Delta )^{- 1}{{\mathcal {W}}}_\infty (- \Delta )&= (- \Delta )^{- 1}{{\mathcal {A}}}_\bot (- \Delta ) (- \Delta )^{- 1}{{\mathcal {B}}} \Phi _\infty (- \Delta ) \\&= \Pi _0^\bot (\langle D \rangle ^{- 2}{{\mathcal {A}}} \langle D \rangle ^2) \Pi _0^\bot (- \Delta )^{- 1}{{\mathcal {B}}} \Phi _\infty (- \Delta ) \\&= \langle D \rangle ^2 (- \Delta )^{- 1} \Pi _0^\bot \langle D \rangle ^{- 2} {{{\mathcal {A}}}} \langle D \rangle ^2 \Pi _0^\bot (- \Delta ) \langle D \rangle ^{- 2} (- \Delta )^{- 1}{{\mathcal {B}}} \Phi _\infty (- \Delta ) \end{aligned} \end{aligned}$$

Hence, the claimed bound follows by Lemmas 5.2,  2.6-(i), (ii), the estimates (4.3) (4.15), (4.56) (for $\varepsilon \gamma ^{- 1} \le \delta < 1$) and by the trivial fact that $\Vert \langle D \rangle ^2 (- \Delta )^{- 1} \Vert _{{{\mathcal {B}}}(H^s)}$, $\Vert (- \Delta ) \langle D \rangle ^{- 2} \Vert _{{{\mathcal {B}}}(H^s)} \lesssim 1$ for any $s \ge 0$. $\square$

We are now ready to prove the invertibility of the operator ${\mathcal {L}}_{\nu }$.

### Proposition 5.4

(Inversion of the operator ${\mathcal {L}}_{\nu }$) There exists $\Sigma _3(\beta ) > \Sigma _2(\beta )$ (where $\Sigma _2(\beta )$ is given in Lemma 5.3) such that, for any $S > s_0 + \Sigma _3(\beta )$, there exists $\delta := \delta (S, \tau , d) \in (0, 1)$ such that, if (3.2) holds and $\varepsilon \gamma ^{- 1} \le \delta$, for any value of the viscosity parameter $\nu > 0$, for any $\lambda = (\omega , \zeta ) \in \Lambda _\infty ^\gamma$ and for any $s_0\le s\le S-\Sigma _3(\beta )$, the operator ${{\mathcal {L}}}_\nu$ is invertible with a bounded inverse ${{\mathcal {L}}}_\nu ^{- 1} \in {{\mathcal {B}}}(H^s_0)$, satisfying the estimates

$$\begin{aligned} \Vert {{\mathcal {L}}}_\nu ^{- 1} \Vert _{{{\mathcal {B}}}(H^s_0)},\, \Vert {{\mathcal {L}}}_\nu ^{- 1} (- \Delta ) \Vert _{{{\mathcal {B}}}(H^s_0)} \lesssim _s \nu ^{- 1}. \end{aligned}$$

### Proof

We write ${{\mathcal {L}}}_\nu ^{- 1} = {{\mathcal {W}}}_\infty ({{\mathcal {L}}}_\nu ^{(\infty )})^{- 1} {{\mathcal {W}}}_\infty ^{- 1}$ and, consequently,

$$\begin{aligned} \begin{aligned} {{\mathcal {L}}}_\nu ^{- 1} (- \Delta )&= {{\mathcal {W}}}_\infty ({{\mathcal {L}}}_\nu ^{(\infty )})^{- 1} {{\mathcal {W}}}_\infty ^{- 1} (- \Delta ) \\&= {{\mathcal {W}}}_\infty \big ( ({{\mathcal {L}}}_\nu ^{(\infty )})^{- 1} (- \Delta ) \big ) \big ( (- \Delta )^{- 1} {{\mathcal {W}}}_\infty ^{- 1} (- \Delta ) \big ). \end{aligned} \end{aligned}$$

Therefore, by Lemmata 5.1, 5.3

$$\begin{aligned} \begin{aligned} \Vert {{\mathcal {L}}}_\nu ^{- 1} (- \Delta )\Vert _{{{\mathcal {B}}}(H^s_0)}&\le \Vert {{\mathcal {W}}}_\infty \Vert _{{{\mathcal {B}}}(H^s_0)}\Vert ({{\mathcal {L}}}_\nu ^{(\infty )})^{- 1} (- \Delta ) \Vert _{{{\mathcal {B}}}(H^s_0)}\Vert (- \Delta )^{- 1} {{\mathcal {W}}}_\infty ^{- 1} (- \Delta )\Vert _{{{\mathcal {B}}}(H^s_0)}\\&\lesssim _s \nu ^{- 1} \end{aligned} \end{aligned}$$

The bound $\Vert {{\mathcal {L}}}_\nu ^{- 1} \Vert _{{{\mathcal {B}}}(H^s_0)} \lesssim _s \nu ^{- 1}$ follows from the latter. $\square$

In order to compute a good approximate solution for the nonlinear equation, we also need to invert the linearized Euler operator ${{\mathcal {L}}}_e$ at the Euler solution $v_e$, see (3.3). To this purpose, we then define the set of the *first Melnikov non-resonance conditions*

5.12 $$\begin{aligned} \begin{aligned} \Gamma _\infty ^\gamma&:= \Big \{ \lambda = (\omega , \zeta ) \in DC(\gamma , \tau ) : \, |{{\textrm{i}}}\, \omega \cdot \ell + \mu _\infty (j)| \ge \frac{\gamma }{\langle \ell \rangle ^\tau |j|^\tau } \\&\qquad \forall \, (\ell , j) \in {\mathbb {Z}}^d \times ({\mathbb {Z}}^2 \setminus \{ 0 \}) \Big \}. \end{aligned} \end{aligned}$$

### Proposition 5.5

(Inversion of the operator ${\mathcal {L}}_{\varepsilon }$) There exists $\Sigma _4(\beta ) > \Sigma _3(\beta )$ large enough (where $\Sigma _3(\beta )$ is given in Proposition 5.4) such that, for any $S > s_0 + \Sigma _4(\beta )$, there exists $\delta := \delta (S, \tau , d) \in (0, 1)$ small enough such that, if (3.2) holds and $\varepsilon \gamma ^{- 1} \le \delta$, for any $\lambda = (\omega , \zeta ) \in \Lambda _\infty ^\gamma \cap \Gamma _\infty ^\gamma$, the operator ${{\mathcal {L}}}_e: H^{s + 1}_0 \rightarrow H^s_0$ is invertible (with loss of derivatives) with the inverse ${{\mathcal {L}}}_e^{- 1} \in {{\mathcal {B}}}(H^{s + \tau }_0, H^s_0)$ for any $s_0\le s \le S-\Sigma _4(\beta )$, satisfying

$$\begin{aligned} \Vert {{\mathcal {L}}}_e^{- 1} \Vert _{{{\mathcal {B}}}(H^{s + \tau }_0, H^s_0)} \lesssim _s \gamma ^{- 1}. \end{aligned}$$

### Proof

By (5.6), one has that ${{\mathcal {L}}}_e = {{\mathcal {W}}}_\infty {{\mathcal {L}}}_{ e}^{(\infty )} {{\mathcal {W}}}_\infty ^{- 1}$ where ${{\mathcal {L}}}_{e}^{(\infty )}$ is defined in (4.57). Thus, for any $\lambda = (\omega , \zeta ) \in \Gamma _\infty ^\gamma$ (see (5.12)), we can invert ${{\mathcal {L}}}_\infty$, its inverse is given by

$$\begin{aligned} ({{\mathcal {L}}}_{e}^{(\infty )})^{- 1} = {\textrm{diag}}_{(\ell , j) \in {\mathbb {Z}}^d \times ({\mathbb {Z}}^2 \setminus \{ 0 \})} \dfrac{1}{{{\textrm{i}}}\,\omega \cdot \ell + \mu _\infty (j)}, \end{aligned}$$

satisfying the estimate $\Vert ({{\mathcal {L}}}_{e}^{(\infty )})^{- 1} \Vert _{{{\mathcal {B}}}(H^{s + \tau }_0, H^s_0)} \le \gamma ^{- 1}$. The claimed statement then follows by Lemma 5.3 and using that ${{\mathcal {L}}}_e^{- 1} = {{\mathcal {W}}}_\infty ({{\mathcal {L}}}_{ e}^{(\infty )})^{- 1} {{\mathcal {W}}}_\infty ^{- 1}$. $\square$

## Approximate Solutions

The purpose of this section is to find an approximate solution up to order $O(\nu ^2)$ of the functional equation ${{\mathcal {F}}}_\nu (v) = 0$ where ${{\mathcal {F}}}_\nu$ is the nonlinear operator defined in (3.1). We actually write

6.1 $$\begin{aligned} \begin{aligned}&{{\mathcal {F}}}_\nu (v) = {\mathtt L}_0 v +\varepsilon {{\mathcal {Q}}}(v) - \varepsilon F - \nu \Delta v , \\&\text {where} \quad {\mathtt L}_0:= \omega \cdot \partial _\varphi + \zeta \cdot \nabla , \quad {{\mathcal {Q}}}(v):= {{\mathcal {N}}}(v, v), \\&{{\mathcal {N}}}(v_1, v_2):= \nabla _\bot (- \Delta )^{- 1} v_1 \cdot \nabla v_2, \end{aligned} \end{aligned}$$

with $\nabla _\bot$ as in (1.4). The map $v \mapsto {{\mathcal {Q}}}(v)$ is a quadratic form. Therefore, for any $v_1, v_2$,

6.2 $$\begin{aligned} \begin{aligned} {{\mathcal {Q}}}(v_1 + v_2)&= {{\mathcal {Q}}}(v_1) + {\mathrm d}{{\mathcal {Q}}}(v_1)[v_2] + {{\mathcal {Q}}}(v_2), \\ {\mathrm d}{{\mathcal {Q}}}(v_1)[v_2]&= {{\mathcal {N}}}(v_1, v_2) + {{\mathcal {N}}}(v_2, v_1). \end{aligned} \end{aligned}$$

By standard Sobolev algebra estimates, one has, for any $v,h \in H^{s+1}_0$, $s\ge s_0$,

6.3 $$\begin{aligned} \begin{aligned} \Vert {{\mathcal {Q}}}(v) \Vert _s&\lesssim _s \Vert v \Vert _{s + 1}^{2}, \quad \Vert {\mathrm d}{{\mathcal {Q}}}(v)[h] \Vert _s \lesssim _s \Vert v \Vert _{s + 1} \Vert h \Vert _{s + 1}. \end{aligned} \end{aligned}$$

We recall the function $v_e$ solves the Euler equation, i.e., (6.1) with $\nu = 0$, and satisfies (3.2):

6.4 $$\begin{aligned} \begin{aligned}&{\mathtt L}_0 v_e +\varepsilon {{\mathcal {Q}}}(v_e) - \varepsilon F = 0 \quad \text {and} \\&\Vert v_e \Vert _S \lesssim _S \varepsilon ^{\mathtt a}, \quad \mathtt a \in (0, 1), \quad S > {{\overline{S}}} \end{aligned} \end{aligned}$$

where ${{\overline{S}}}$ is given in Theorem 1.1. We now prove the following proposition.

### Proposition 6.1

(Approximate solutions). There exists ${{\overline{\mu }}} > \Sigma _4(\beta )$ large enough (where $\Sigma _4(\beta )$ is given in Proposition 5.5) such that, for any $S \ge {\textrm{max}}\{ {{\overline{S}}}\,,\,s_0 + {{\overline{\mu }}} \}$, there exists $\delta := \delta (S, \tau , d) \in (0, 1)$ small enough such that, if $\varepsilon ^{\mathtt a} \gamma ^{- 1} \le \delta$, for any $\lambda = (\omega , \zeta ) \in \Lambda _\infty ^\gamma \cap \Gamma _\infty ^\gamma$ (see (4.53), (5.12)), there exists an approximate solution of the form $v_{app} = v_e + \nu v_1$ of the functional equation ${{\mathcal {F}}}_\nu (v) = 0$ with the following properties:

6.5 $$\begin{aligned} \begin{aligned}&v_1 \in H^s_0, \quad \Vert v_1 \Vert _s \lesssim _s \varepsilon ^{\mathtt a} \gamma ^{- 1}, \\&\Vert {{\mathcal {F}}}_{\nu }(v_{app}) \Vert _s \lesssim _s \varepsilon ^{\mathtt a} \gamma ^{- 1} \nu ^2 , \quad \forall s_0 \le s \le S - {{\overline{\mu }}}. \end{aligned} \end{aligned}$$

### Proof

We look for an approximate solution up to order $O(\nu ^2)$ of the form

$$\begin{aligned} v_{app}:= v_e + \nu v_1 \end{aligned}$$

where $v_e$ is the solution of the Euler equation in (6.4) and $v_1$ has to be determined. By (6.4), one has

6.6 $$\begin{aligned} \begin{aligned} {{\mathcal {F}}}_{\nu }(v_e + \nu v_1)&= {\mathtt L}_0 v_e +\varepsilon {{\mathcal {Q}}}(v_e) - \varepsilon F + \nu \big ( {\mathtt L}_0 v_1 + \varepsilon {\mathrm d}{{\mathcal {Q}}}(v_e) v_1 - \Delta v_e \big ) \\&\quad + \nu ^2 \big ( - \Delta v_1+ \varepsilon {{\mathcal {Q}}}(v_1) \big ) \\&= \nu \big ( {\mathtt L}_0 v_1 + \varepsilon {{\mathcal {Q}}}'(v_e) v_1 - \Delta v_e \big ) + \nu ^2 \big ( - \Delta v_1+ \varepsilon {{\mathcal {Q}}}(v_1) \big ). \end{aligned} \end{aligned}$$

We want to choose $v_1$ in such a way that

6.7 $$\begin{aligned} {\mathtt L}_0 v_1 + \varepsilon {\mathrm d}{{\mathcal {Q}}}(v_e) v_1 - \Delta v_e = 0. \end{aligned}$$

Note that $\Delta v_e$ has zero space average and ${\mathtt L}_0 + \varepsilon {\mathrm d}{{\mathcal {Q}}}(v_e) = {{\mathcal {L}}}_e$, see (3.3)–(3.5). Hence, by Proposition 5.5 and by (6.4), for some ${{\overline{\mu }}} \gg 0$ sufficiently large and for any $S > s_0 + {{\overline{\mu }}}$, if (3.2) holds and $\varepsilon ^{\mathtt a} \gamma ^{- 1} \le \delta < 1$, we define, for any $\lambda = (\omega , \zeta ) \in \Lambda _\infty ^\gamma \cap \Gamma _\infty ^\gamma$,

6.8 $$\begin{aligned} v_1:= {{\mathcal {L}}}_e^{- 1} \Delta v_e \in H_0^{s + 2}, \quad \Vert v_1 \Vert _{s + 2} \lesssim _s \varepsilon ^{\mathtt a} \gamma ^{- 1}, \quad \forall s_0 \le s \le S - {{\overline{\mu }}} \end{aligned}$$

By (6.4), (6.6), (6.7) and estimates (6.3), (6.8) (using also $\varepsilon ^{\mathtt a} \gamma ^{- 1} \le \delta < 1$ sufficiently small) we conclude that

$$\begin{aligned} \begin{aligned} \Vert {{\mathcal {F}}}_\nu (v_e + \nu v_1) \Vert _s&= \nu ^2\Vert - \Delta v_1 + \varepsilon {{\mathcal {Q}}}(v_1) \Vert _s \le \nu ^2 \big ( \Vert \Delta v_1 \Vert _s + \Vert {{\mathcal {Q}}}(v_1) \Vert _s\big ) \\&\le \nu ^2 \big ( \Vert v_1 \Vert _{s + 2} + \Vert v_1 \Vert _{s + 1}^2 \big ) \lesssim _s \nu ^2 \varepsilon ^{\mathtt a} \gamma ^{- 1}. \end{aligned} \end{aligned}$$

The claimed statement has then been proved. $\square$

## The Fixed Point Argument

In this section we want to find a solution *v* of the functional equation ${{\mathcal {F}}}_\nu (v) = 0$ bifurcating from the approximate solution $v_{app} = v_e + \nu v_1$ constructed in the previous section. We search for a solution of the form $v = v_{app} + \psi$. By (6.1), (6.2) and using that $v_{app} = v_e + \nu v_1$, one computes

7.1 $$\begin{aligned} \begin{aligned} {{\mathcal {F}}}_\nu (v_{app} + \psi )&= {{\mathcal {F}}}_\nu (v_{app}) + \big ( {\mathtt L}_0 + \varepsilon {\mathrm d}{{\mathcal {Q}}}(v_e) - \nu \Delta \big )[\psi ] + \varepsilon \nu {\mathrm d}{{\mathcal {Q}}}(v_1)[\psi ] + \varepsilon {{\mathcal {Q}}}(\psi ) \\&= {{\mathcal {F}}}_\nu (v_{app}) + {{\mathcal {L}}}_\nu \psi + \varepsilon \, \nu \, d {{\mathcal {Q}}}(v_1)[\psi ] + \varepsilon \,{{\mathcal {Q}}}(\psi ) \end{aligned}\nonumber \\ \end{aligned}$$

since the linear operator ${\mathtt L}_0 + \varepsilon {\mathrm d}{{\mathcal {Q}}}(v_e)- \nu \Delta$ is exactly the linearized Navier Stokes operator ${\mathcal {L}}_{\nu }$ in (3.3) obtained by linearizing the nonlinear functional ${{\mathcal {F}}}_\nu$ at the Euler solution $v_e$. Therefore, the equation ${{\mathcal {F}}}_\nu (v_{app} + \psi ) = 0$ reduces to

$$\begin{aligned} {{\mathcal {L}}}_\nu \psi = - \big ({{\mathcal {F}}}_{\nu }(v_{app}) + \varepsilon \, \nu \, {\mathrm d}{{\mathcal {Q}}}(v_1)[\psi ] + \varepsilon \, {{\mathcal {Q}}}(\psi )\big ). \end{aligned}$$

By the invertibility of the linear operator ${{\mathcal {L}}}_\nu$, proved in Proposition 5.4 for any $\nu >0$, we conclude that finding solutions of ${{\mathcal {F}}}_{\nu }(v_{app} + \psi ) = 0$ is equivalent to solve a fixed point problem, that is

7.2 $$\begin{aligned} \begin{aligned}&\psi = {{\mathcal {S}}}_\nu (\psi ) \quad \text {where} \\&{{\mathcal {S}}}_\nu (\psi ):= - {{\mathcal {L}}}_\nu ^{- 1}\big ({{\mathcal {F}}}_{\nu }(v_{app}) + \varepsilon \,\nu \, {\mathrm d}{{\mathcal {Q}}}(v_1)[\psi ] + \varepsilon {{\mathcal {Q}}}(\psi )\big ). \end{aligned} \end{aligned}$$

For any $s \ge 0$, $\eta > 0$, we define the closed ball

$$\begin{aligned} {{\mathcal {B}}}_s(\eta ):= \big \{ z \in H^s_0: \Vert z \Vert _s \le \eta \big \}. \end{aligned}$$

We want to show that for $S > {\textrm{max}}\{ {{\overline{S}}}\,,\,s_0 + {{\overline{\mu }}} \}$ (as in Proposition 6.1) (where the constant ${{\overline{\mu }}} \gg 0$ is given in Proposition 6.1) if (6.4) holds and $\varepsilon ^{\mathtt a} \gamma ^{- 1} \ll 1$, for any $s_0 \le s \le S - {{\overline{\mu }}}$ and for any value of the viscosity parameter $\nu > 0$, the map ${{\mathcal {S}}}_\nu : {{\mathcal {B}}}_s(\nu ) \rightarrow {{\mathcal {B}}}_s(\nu )$ is a contraction. We actually need this preliminary lemma which allows to estimate in a sharp way the terms ${\mathrm d}{{\mathcal {Q}}}(v_1)[\psi ]$ and ${{\mathcal {Q}}}(\psi )$ appearing in (7.2).

### Lemma 7.1

For $s\ge s_0$, we define

$$\begin{aligned} {\mathcal {N}}:H^{s + 1}_0\times H^{s + 1}_0 \rightarrow H^{s}_0 , \quad (v_1,v_2) \mapsto {{\mathcal {N}}}(v_1, v_2):= \big ( \nabla _\bot (- \Delta )^{- 1} v_1 \big ) \cdot \nabla v_2 \end{aligned}$$

where $\nabla _\bot$ is as in (1.4). Then for any $n \in {\mathbb {N}}$, $s \ge s_0$,

$$\begin{aligned} \Vert (- \Delta )^{- \frac{n}{2}}{{\mathcal {N}}}(v_1, v_2) \Vert _s \lesssim _s \Vert v_1 \Vert _s \Vert v_2 \Vert _s, \quad \forall v_1, v_2 \in H^s. \end{aligned}$$

### Proof

First of all, note that if $v_1, v_2$ have zero space average, using that ${\textrm{div}}\big (\nabla _\bot \langle D \rangle ^{- 2} v_1 \big ) = 0$ and by integrating by parts, one can easily see that ${{\mathcal {N}}}(v_1, v_2)$ has zero space average. Let $v_1, v_2 \in H^s_0$ and expand the bilinear form ${{\mathcal {N}}}(v_1, v_2)$ in Fourier coefficients. One obtains

7.3 $$\begin{aligned} (- \Delta )^{- \frac{n}{2}} {{\mathcal {N}}}(v_1, v_2) = \sum _{{\begin{array}{c} \xi _1, \xi _2 \in {\mathbb {Z}}^2 \setminus \{ 0 \} \\ \ell _1, \ell _2 \in {\mathbb {Z}}^d \\ \xi _1 + \xi _2 \ne 0 \end{array}}} N(\xi _1, \xi _2) {{\widehat{v}}}_1(\ell _1, \xi _1) {{\widehat{v}}}_2(\ell _2, \xi _2) e^{{{\textrm{i}}}(\ell _1 + \ell _2) \cdot \varphi } e^{{{\textrm{i}}}(\xi _1 + \xi _2) \cdot x}\nonumber \\ \end{aligned}$$

where

$$\begin{aligned} N(\xi _1, \xi _2):= {{\textrm{i}}}\dfrac{\xi _1^\bot \cdot \xi _2}{| \xi _1 + \xi _2 |^n | \xi _1 |^2}, \quad \xi _1, \xi _2 \in {\mathbb {Z}}^2 \setminus \{ 0 \}, \quad \xi _1 + \xi _2 \ne 0 \end{aligned}$$

where $y^\bot := (- y_2, y_1)$ for $y = (y_1, y_2)$. Using that $|\xi _1^\perp |\le |\xi _1 |$ and $| \xi _2 | \lesssim | \xi _1 | + | \xi _1 + \xi _2 | \lesssim | \xi _1 | |\xi _1 + \xi _2|$ for any $\xi _1, \xi _2 \in {\mathbb {Z}}^2 {\setminus } \{ 0 \}$ with $\xi _1 + \xi _2 \ne 0$, one has

7.4 $$\begin{aligned} \begin{aligned} |N(\xi _1, \xi _2)|&\le \dfrac{|\xi _1^\bot | | \xi _2|}{\langle \xi _1 + \xi _2 \rangle ^n \langle \xi _1 \rangle ^2} \le \dfrac{ | \xi _2|}{| \xi _1 + \xi _2 |^n | \xi _1 |} \lesssim 1, \end{aligned} \end{aligned}$$

uniformly in $\xi _1,\xi _2 \in {\mathbb {Z}}^{2} {\setminus } \{ 0 \}$, $\xi _1 + \xi _2 \ne 0$. Therefore, by (7.3), (7.4) and using that $\langle \ell , \xi \rangle ^s \lesssim _s \langle \ell ', \xi ' \rangle ^s + \langle \ell - \ell ', \xi - \xi ' \rangle ^s$ for any $\ell , \ell ' \in {\mathbb {Z}}^d$, $\xi , \xi ' \in {\mathbb {Z}}^2$, one has

$$\begin{aligned} \begin{aligned}&\Vert (- \Delta )^{- \frac{n}{2}} {{\mathcal {N}}}(v_1, v_2) \Vert _s^2 \\&= \sum _{(\ell , \xi ) \in {\mathbb {Z}}^{d} \times ({\mathbb {Z}}^2 \setminus \{ 0 \})} \langle \ell , \xi \rangle ^{2s} \Big | \sum _{(\ell ', j') \in {\mathbb {Z}}^{d} \times ({\mathbb {Z}}^2 \setminus \{ 0 \})} N(\xi - \xi ', \xi ') {{\widehat{v}}}_1(\ell - \ell ', \xi - \xi ') {{\widehat{v}}}_2(\ell ', \xi ') \Big |^2 \\&\lesssim \sum _{(\ell , \xi ) \in {\mathbb {Z}}^{d} \times ({\mathbb {Z}}^2 \setminus \{ 0 \})} \langle \ell , \xi \rangle ^{2s} \Big ( \sum _{(\ell ', \xi ') \in {\mathbb {Z}}^{d} \times ({\mathbb {Z}}^2 \setminus \{ 0 \})} |{{\widehat{v}}}_1(\ell - \ell ', \xi - \xi ') ||{{\widehat{v}}}_2(\ell ', \xi ')| \Big )^2 \\&\lesssim _s A_1 + A_2 , \end{aligned} \end{aligned}$$

where

$$\begin{aligned} \begin{aligned} A_1&:= \sum _{(\ell , \xi ) \in {\mathbb {Z}}^{d} \times ({\mathbb {Z}}^2 \setminus \{ 0 \})} \Big ( \sum _{(\ell ', \xi ') \in {\mathbb {Z}}^{d} \times ({\mathbb {Z}}^2 \setminus \{ 0 \})} \langle \ell ', \xi ' \rangle ^{s} |{{\widehat{v}}}_1(\ell - \ell ', \xi - \xi ') ||{{\widehat{v}}}_2(\ell ', \xi ')| \Big )^2 , \\ A_2&:= \sum _{(\ell , \xi ) \in {\mathbb {Z}}^{d} \times ({\mathbb {Z}}^2 \setminus \{ 0 \})} \Big ( \sum _{(\ell ', \xi ') \in {\mathbb {Z}}^{d} \times ({\mathbb {Z}}^2 \setminus \{ 0 \})} \langle \ell - \ell ', \xi - \xi ' \rangle ^{s} |{{\widehat{v}}}_1(\ell - \ell ', \xi - \xi ') ||{{\widehat{v}}}_2(\ell ', \xi ')| \Big )^2. \end{aligned} \end{aligned}$$

We estimate $A_1$. The estimate of $A_2$ can be done similarly. By the Cauchy-Schwartz inequality and using that $\sum _{\ell ', \xi '} \frac{1}{\langle \ell - \ell ', \xi - \xi ' \rangle ^{2 s_0}} \le C_0$ (since $s_0 > \frac{d + 2}{2}$, see (2.2)), one has

$$\begin{aligned} \begin{aligned} A_1&\lesssim \sum _{{\begin{array}{c} (\ell , \xi ) \in {\mathbb {Z}}^{d} \times ({\mathbb {Z}}^2 \setminus \{ 0 \}) \\ (\ell ', \xi ') \in {\mathbb {Z}}^{d} \times ({\mathbb {Z}}^2 \setminus \{ 0 \}) \end{array}}}\langle \ell - \ell ', \xi - \xi ' \rangle ^{2 s_0} |{{\widehat{v}}}_1(\ell - \ell ', \xi - \xi ') |^2 \langle \ell ', \xi ' \rangle ^{2 s} |{{\widehat{v}}}_2(\ell ', \xi ')|^2 \\&\lesssim \sum _{(\ell ', \xi ') \in {\mathbb {Z}}^{d} \times ({\mathbb {Z}}^2 \setminus \{ 0 \})} \langle \ell ', \xi ' \rangle ^{2 s} |{{\widehat{v}}}_2(\ell ', \xi ')|^2 \sum _{(\ell , \xi ) \in {\mathbb {Z}}^{d} \times ({\mathbb {Z}}^2 \setminus \{ 0 \})} \langle \ell - \ell ', \xi - \xi ' \rangle ^{2 s_0} |{{\widehat{v}}}_1(\ell - \ell ', \xi - \xi ') |^2 \\&\lesssim \Vert v_2 \Vert _s^2 \Vert v_1 \Vert _{s_0}^2. \end{aligned} \end{aligned}$$

Similarly, one shows that $A_2 \lesssim \Vert v_2 \Vert _{s_0}^2 \Vert v_1 \Vert _s^2$ and the claimed estimate follows. $\square$

We are now ready to perform a fixed point argument on the map ${{\mathcal {S}}}_\nu$ defined in (7.2).

### Proposition 7.2

(Contraction). For any $S > {\textrm{max}}\{ {{\overline{S}}}\,,\, s_0 + {{\overline{\mu }}} \}$ (where ${{\overline{\mu }}} \gg 0$ is the constant given in Proposition 6.1) there exists $\delta := \delta (S, \tau , d) \in (0, 1)$ such that, if (6.4) holds and $\varepsilon ^{\mathtt a} \gamma ^{- 1} \le \delta$, for any $s_0 \le s \le S - {{\overline{\mu }}}$, for any value of the viscosity $\nu > 0$ and for any value of the parameter $\lambda = (\omega , \zeta ) \in \Lambda _\infty ^\gamma \cap \Gamma _\infty ^\gamma$ (see (4.53), (5.12)), the map ${{\mathcal {S}}}_\nu : {{\mathcal {B}}}_s(\nu ) \rightarrow {{\mathcal {B}}}_s(\nu )$, defined in (7.2), is a contraction.

### Proof

We write the map ${{\mathcal {S}}}_\nu$ as

7.5 $$\begin{aligned} \begin{aligned} {{\mathcal {S}}}_\nu (\psi )&:= - {{\mathcal {L}}}_\nu ^{- 1}{{\mathcal {F}}}_\nu (v_{app}) - \varepsilon \, \nu \, \big ({{\mathcal {L}}}_\nu ^{- 1} (- \Delta ) \big ) \big ((- \Delta )^{- 1} {\mathrm d}{{\mathcal {Q}}}(v_1)[\psi ] \big ) \\&\quad - \varepsilon \, \big ({{\mathcal {L}}}_\nu ^{- 1} (- \Delta ) \big ) \big ((- \Delta )^{- 1}{{\mathcal {Q}}}(\psi ) \big ). \end{aligned} \end{aligned}$$

Let $s_0 \le s \le S - {{\overline{\mu }}}$, $\nu > 0$, $\psi \in {{\mathcal {B}}}_s(\nu )$. By Proposition 5.4, one has that

7.6 $$\begin{aligned} \begin{aligned} \Vert {{\mathcal {S}}}_\nu (\psi ) \Vert _s&\lesssim _s \nu ^{- 1} \big ( \Vert {{\mathcal {F}}}_\nu (v_{app})\Vert _s + \varepsilon \nu \Vert (- \Delta )^{- 1}{\mathrm d}{{\mathcal {Q}}}(v_1)[\psi ] \Vert _s \\&\qquad + \varepsilon \Vert (- \Delta )^{- 1}{{\mathcal {Q}}}(\psi ) \Vert _s \big ). \end{aligned} \end{aligned}$$

By (6.1)–(6.2), Proposition 6.1, Lemma 7.1, and using that $\Vert \psi \Vert _s \le \nu$, one gets

$$\begin{aligned} \begin{aligned}&\Vert {{\mathcal {F}}}_\nu (v_{app})\Vert _s \lesssim _s \nu ^2 \varepsilon ^{\mathtt a} \gamma ^{- 1}, \quad \Vert (- \Delta )^{- 1} {\mathrm d}{{\mathcal {Q}}}(v_1)[\psi ] \Vert _s \lesssim _s \nu \varepsilon ^{\mathtt a} \gamma ^{- 1}, \\&\Vert (- \Delta )^{- 1}{{\mathcal {Q}}}(\psi ) \Vert _s \lesssim _s \nu ^2 . \end{aligned} \end{aligned}$$

The latter bounds, together with (7.6) and having ${\mathtt a}\in (0,1)$, imply that

$$\begin{aligned} \begin{aligned} \Vert {{\mathcal {S}}}_\nu (\psi ) \Vert _s&\lesssim _s \nu ^{- 1} \big (\nu ^2 \varepsilon ^{\mathtt a} \gamma ^{- 1} + \varepsilon ^{\mathtt a + 1} \nu ^2 \gamma ^{- 1} + \varepsilon \nu ^2 \big ) \le C(s) \nu \varepsilon ^{\mathtt a} \gamma ^{- 1} \end{aligned} \end{aligned}$$

for some constant $C(s) > 0$. Therefore, by taking $C(s) \varepsilon ^{\mathtt a} \gamma ^{- 1} \le 1$, we get that ${\mathcal {S}}_\nu$ maps ${{\mathcal {B}}}_s(\nu )$ into itself. By (7.5), for any $\psi \in {{\mathcal {B}}}_s(\nu )$, one computes the Fréchet differential of ${{\mathcal {S}}}_\nu$

$$\begin{aligned} \begin{aligned} {\mathrm d}\,{{\mathcal {S}}}_\nu (\psi )&= - \varepsilon \nu \big ({{\mathcal {L}}}_\nu ^{- 1} (- \Delta ) \big ) \big ( (- \Delta )^{- 1} {\mathrm d}{{\mathcal {Q}}}(v_1) \big ) \\&\quad - \varepsilon \big ({{\mathcal {L}}}_\nu ^{- 1} (- \Delta ) \big ) \big ((- \Delta )^{- 1} {\mathrm d}{\mathcal {Q}}(\psi ) \big ) . \end{aligned} \end{aligned}$$

Arguing as above, we obtain

$$\begin{aligned} \Vert {\mathrm d}\,{{\mathcal {S}}}_\nu (\psi )\Vert _{{{\mathcal {B}}}(H^s_0)} \le C(s) \big ( \varepsilon ^{\mathtt a + 1} \gamma ^{- 1} + \varepsilon \big ) \le C(s) \varepsilon \end{aligned}$$

for some larger constant $C(s) > 0$, asking again $\varepsilon ^{\mathtt a} \gamma ^{- 1} \le \tfrac{1}{C(s)}$. By taking $C(s) \varepsilon \le 1/2$, we conclude that $\Vert {\mathrm d}\,{{\mathcal {S}}}_\nu ( \psi ) \Vert _{{{\mathcal {B}}}(H^s_0)} \le 1/2$, implying that ${{\mathcal {S}}}_\nu$ is a contraction. $\square$

## Proof of Theorem 1.2

In this final section we summarize the whole construction and we conclude the proof of Theorem 1.2. In the first place, we show that the non-resonance conditions (4.53) and (5.12) hold for most values of the parameters $(\omega ,\zeta )\in {\mathbb {R}}^d\times {\mathbb {R}}^2$.

### Measure Estimate

In this section we prove that the set

8.1 $$\begin{aligned} {{\mathcal {G}}}_\infty ^\gamma := \Lambda _\infty ^\gamma \cap \Gamma _\infty ^\gamma \end{aligned}$$

has large Lebesgue measure (recall (4.53), (5.12)). We actually show the following

#### Proposition 8.1

Let

8.2 $$\begin{aligned} \tau := {\textrm{max}}\{ d, 2 \} + 1. \end{aligned}$$

Then $|\Omega _\varepsilon {\setminus } {{\mathcal {G}}}_\infty ^\gamma | \lesssim \gamma$ (recall that $\Omega _\varepsilon$ is the set of parameters appearing in Theorem 1.1).

The rest of this section is devoted to the proof of the latter proposition. We actually estimate the measure of the set $\Omega _\varepsilon {\setminus } \Lambda _\infty ^\gamma$. The estimate for $\Omega _\varepsilon {\setminus } \Gamma _\infty ^\gamma$ follows similarly. We write

8.3 $$\begin{aligned} \Omega _\varepsilon \setminus \Lambda _\infty ^\gamma = (\Omega _\varepsilon \setminus DC(\gamma , \tau )) \cup (DC(\gamma , \tau ) \setminus \Lambda _\infty ^\gamma ). \end{aligned}$$

By a standard Diophantine estimate one shows that

8.4 $$\begin{aligned} |\Omega _\varepsilon \setminus DC(\gamma , \tau )| \lesssim \gamma . \end{aligned}$$

Define

$$\begin{aligned} {{\mathcal {I}}}:= \Big \{ (\ell , j, j') \in {\mathbb {Z}}^d \times ({\mathbb {Z}}^2 \setminus \{ 0\}) \times ({\mathbb {Z}}^2 \setminus \{ 0\}): (\ell , j, j') \ne (0, j, j) \Big \}. \end{aligned}$$

By recalling (4.53), one has that

8.5 $$\begin{aligned} \begin{aligned}&\Lambda _\infty ^\gamma \setminus DC(\gamma , \tau ) \subseteq \bigcup _{(\ell , j, j') \in {{\mathcal {I}}}} {{\mathcal {R}}}_\gamma (\ell , j, j'), \\&{{\mathcal {R}}}_\gamma (\ell , j, j'):= \Big \{ \lambda = (\omega , \zeta ) \in DC(\gamma , \tau ): |{{\textrm{i}}}\,\omega \cdot \ell + \mu _\infty (j) - \mu _\infty (j')| < \frac{2 \gamma }{\langle \ell \rangle ^\tau |j|^\tau |j'|^\tau } \Big \}. \end{aligned}\nonumber \\ \end{aligned}$$

#### Lemma 8.2

We have $|{{\mathcal {R}}}_\gamma (\ell , j, j')| \lesssim \gamma \langle \ell \rangle ^{- \tau } |j|^{- \tau } |j'|^{- \tau }$ for any $(\ell , j, j') \in {{\mathcal {I}}}$.

#### Proof

By Lemma 4.8, one gets that

$$\begin{aligned} \begin{aligned}&{{\textrm{i}}}\,\omega \cdot \ell + \mu _\infty (j) - \mu _\infty (j') = {{\textrm{i}}}\lambda \cdot k + r_{j j'}(\lambda ) =: \varphi (\lambda ), \\&\lambda = (\omega , \zeta ), \quad k = (\ell , j - j'), \quad |r_{j j'}|^{{\textrm{Lip}}(\gamma )} \lesssim \varepsilon . \end{aligned} \end{aligned}$$

Since $(\ell , j, j') \in {{\mathcal {I}}}$, one has $k \ne 0$. We write $\lambda = \frac{k}{|k|}s + \eta$, with $\eta \cdot k = 0$, and

$$\begin{aligned} \begin{aligned}&\varphi (s):= {{\textrm{i}}}\,\lambda \cdot k + r_{j j'}(\lambda ) = {{\textrm{i}}}s |k| + {{\tilde{r}}}_{j j'}(s), \quad {{\tilde{r}}}_{j j'}(s):= r_{j j'}\big (\tfrac{k}{|k|}s + \eta \big ). \end{aligned} \end{aligned}$$

One then gets

$$\begin{aligned} |\varphi (s_1) - \varphi (s_2)| \ge (|k| - |r_{j j'}|^{\textrm{lip}}) |s_1 - s_2| \ge (|k| - C \varepsilon \gamma ^{- 1}) |s_1 - s_2| \ge \tfrac{1}{2} |s_1 - s_2| \end{aligned}$$

assuming $\varepsilon \gamma ^{- 1} \le \tfrac{1}{2C}$. This implies that

$$\begin{aligned} \Big | \Big \{ s: |\varphi (s)| < \frac{2 \gamma }{\langle \ell \rangle ^\tau |j|^\tau |j'|^\tau }\Big \} \Big | \lesssim \frac{ \gamma }{\langle \ell \rangle ^\tau |j|^\tau |j'|^\tau } \end{aligned}$$

The claimed measure estimate then follows by a Fubini argument. $\square$

#### Proof of Proposition 8.1

By (8.5) and Lemma 8.2, one gets that $|DC(\gamma , \tau ) \setminus \Lambda _\infty ^\gamma |$ can be bounded by $C \gamma \sum _{\ell , j j'} \langle \ell \rangle ^{- \tau } \langle j \rangle ^{- \tau } \langle j' \rangle ^{- \tau }$. This series converges by taking $\tau$ as in (8.2). By recalling also (8.3), (8.4) one estimates $\Omega \setminus \Lambda _\infty ^\gamma$. The estimate for $\Omega \setminus \Gamma _\infty ^\gamma$ is similar. $\square$

### Proof of Theorem 1.2

We finally prove the main result of Theorem 1.2 together with Corollary 1.3. At this final stage, we link the constant $\gamma$, coming from the non-resonance conditions in the set (8.1), with the small parameter $\varepsilon >0$ by choosing the former as

8.6 $$\begin{aligned} \gamma := \varepsilon ^{\frac{\mathtt a}{2}} \end{aligned}$$

#### Proof of Theorem 1.2

Let $s\ge s_0$ be fixed and $\gamma$ as in (8.6). Then, the smallness conditions $\varepsilon ^{{\mathtt a}}\gamma ^{-1} = \varepsilon ^{\frac{\mathtt a}{2}}\le \delta < 1$ is satisfied for $\varepsilon \le \varepsilon _0< 1$ small enough. The time quasi-periodic solution of (1.4) is searched as zero of the nonlinear functional ${\mathcal {F}}_{\nu }(v)$ defined in (3.1). We look for the solution $v_{\nu }=v_{\nu }(\varphi ,x;\omega ,\zeta )$ of the form $v_{\nu }=v_{e}+ \nu v_1 +\psi _\nu$. It depends on the parameters $\lambda = (\omega , \zeta ) \in {{\mathcal {O}}}_\varepsilon := {{\mathcal {G}}}_\infty ^\gamma$, where ${{\mathcal {G}}}_\infty ^{\gamma }$ is defined in (8.1). The function $v_e(\cdot ; \lambda ) \in H_0^{S}({\mathbb {T}}^{d+2})$, $\lambda \in \Omega _\varepsilon$ is a solution of the forced Euler equation provided by Theorem 1.1 with $S = {\textrm{max}}\{s + {{\overline{\mu }}}, {{\overline{S}}} \}$, which fixes the regularity $q=q(S)$ of the forcing term $F=\nabla \times f$. It satisfies $\sup _{(\omega , \zeta ) \in {{\mathcal {G}}}_\infty ^\gamma }\Vert v_e(\,\cdot \,;\omega ,\zeta ) \Vert _{s+{{\overline{\mu }}}} \lesssim _{s} \varepsilon ^{\mathtt a}$, for some $\mathtt a \in (0,1)$ independent of *s*. The function $v_1\in H_0^{s}({\mathbb {T}}^{d+2})$ is provided by Proposition 6.1: It is defined as in (6.8) and satisfies $\sup _{(\omega , \zeta ) \in {{\mathcal {G}}}_\infty ^\gamma }\Vert v_1 (\,\cdot \,;\omega ,\zeta )\Vert _{s} \le C(s) \varepsilon ^{\mathtt a}\gamma ^{-1} \le 1$. The definition of the function $v_1$ implies that $v_e +\nu v_1$ solves the equation up to an error of order $O(\nu ^2)$, namely $\Vert {\mathcal {F}}_{\nu }(v_e+\nu v_1)\Vert _{s} \le C(s)\nu ^2 \varepsilon ^{\mathtt a}\gamma ^{-1}\le \nu ^2$, see (6.5). The function $\psi _\nu$ is then defined as the fixed point for the map ${\mathcal {S}}_\nu (\psi )$ in (7.1)–(7.2). Proposition 7.2 shows that ${\mathcal {S}}_\nu$ is a contraction map on the ball ${\mathcal {B}}_s(\nu )$, which implies the of a unique $\psi _\nu$ in the ball $\Vert \psi _\nu \Vert _{s} \le \nu$ such that $\psi _\nu ={\mathcal {S}}_\nu (\psi _\nu )$. We conclude that ${\mathcal {F}}_{\nu }(v_e + \nu v_1 + \psi _\nu )=0$ and that $\sup _{(\omega , \zeta ) \in {{\mathcal {G}}}_\infty ^\gamma } \Vert v_\nu (\,\cdot \,; \omega ,\zeta ) - v_e (\,\cdot \,; \omega ,\zeta ) \Vert _s \le 2\nu$ with $v_\nu := v_e + \nu v_1 + \psi _\nu$, as required. Finally, using that $| \Omega {\setminus } {{\mathcal {O}}}_\varepsilon | \le |\Omega {\setminus } \Omega _\varepsilon | + |\Omega {\setminus } {{\mathcal {G}}}_\infty ^{\gamma } |$, we conclude that $\lim _{\varepsilon \rightarrow 0}| \Omega {\setminus } {{\mathcal {O}}}_\varepsilon | =0$ by Theorem 1.1, Proposition 8.1 and using that we have chosen $\gamma = \varepsilon ^{\frac{\mathtt a}{2}}$ as in (8.6). $\square$

#### Proof

Let $v_e \in H^{s+{{\overline{\mu }}}}_0({\mathbb {T}}^{d + 2})$ and $v_\nu \in H^s_0({\mathbb {T}}^{d + 2})$ as in Theorem 1.2 satisfying $\Vert v_\nu (\cdot ; \lambda ) - v_e (\cdot ; \lambda ) \Vert _s \lesssim _s \nu$ for any $\lambda = (\omega , \zeta ) \in {{\mathcal {O}}}_\varepsilon$. Let

$$\begin{aligned} v_{\nu }^\omega (t, x):= v_\nu (\omega t, x), \quad v_e^\omega (t, x):= v_e(\omega t, x), \quad (t, x) \in {\mathbb {R}}\times {\mathbb {T}}^2. \end{aligned}$$

$v_\nu ^\omega$ is a global solution of the forced Navier–Stokes equation and $v_e^\omega$ is a global solution of the forced Euler equation with external force $F(\omega t, x)$, $F:= \nabla \times f$. By the latter definition, one clearly has

$$\begin{aligned} \Vert v_{\nu }^\omega - v_e^\omega \Vert _{W^{\sigma , \infty }({\mathbb {R}}\times {\mathbb {T}}^2)} \lesssim \Vert v_\nu - v_e \Vert _{W^{\sigma , \infty }({\mathbb {T}}^d \times {\mathbb {T}}^2)} \end{aligned}$$

and using that $H^{s}({\mathbb {T}}^d \times {\mathbb {T}}^2)$ is compactly embedded in $W^{\sigma , \infty }({\mathbb {T}}^d \times {\mathbb {T}}^2)$ with $0 \le \sigma \le s - \big ( \lfloor \frac{d + 2}{2} \rfloor + 1\big )$, one obtains the chain of inequalities

$$\begin{aligned} \begin{aligned} \Vert v_{\nu }^\omega - v_e^\omega \Vert _{W^{\sigma , \infty }({\mathbb {R}}\times {\mathbb {T}}^2)}&\lesssim \Vert v_\nu - v_e \Vert _{W^{\sigma , \infty }({\mathbb {T}}^d \times {\mathbb {T}}^2)} \lesssim \Vert v_\nu - v_e \Vert _s \lesssim _s \nu . \end{aligned} \end{aligned}$$

The proof of the Corollary is then concluded. $\square$
